# DNA microarray revealed and RNAi plants confirmed key genes conferring low Cd accumulation in barley grains

**DOI:** 10.1186/s12870-015-0648-5

**Published:** 2015-10-26

**Authors:** Hongyan Sun, Zhong-Hua Chen, Fei Chen, Lupeng Xie, Guoping Zhang, Eva Vincze, Feibo Wu

**Affiliations:** Department of Agronomy, College of Agriculture and Biotechnology, Zijingang Campus, Zhejiang University, Hangzhou, 310058 PR China; Jiangsu Co-Innovation Center for Modern Production Technology of Grain Crops, Yangzhou University, Yangzhou, 225009 China; Department of Molecular Biology and Genetics, Aarhus University, Slagelse, Denmark

**Keywords:** Antisense, Barley (*Hordeum vulgare* L.), Cadmium (Cd), Low-grain-Cd-accumulation, Transcriptome analysis, ZIP transporter

## Abstract

**Background:**

Understanding the mechanism of low Cd accumulation in crops is crucial for sustainable safe food production in Cd-contaminated soils.

**Results:**

Confocal microscopy, atomic absorption spectrometry, gas exchange and chlorophyll fluorescence analyses revealed a distinct difference in Cd accumulation and tolerance between the two contrasting barley genotypes: W6nk2 (a low-grain-Cd-accumulating and Cd-sensitive genotype) and Zhenong8 (a high-grain-Cd-accumulating and tolerant genotype). A DNA microarray analysis detected large-scale changes of gene expression in response to Cd stress with a substantial difference between the two genotypes. Cd stress led to higher expression of genes involved in transport, carbohydrate metabolism and signal transduction in the low-grain-Cd-accumulating genotype. Novel transporter genes such as zinc transporter genes were identified as being associated with low Cd accumulation. Quantitative RT-PCR confirmed our microarray data. Furthermore, suppression of the zinc transporter genes *HvZIP3* and *HvZIP8* by RNAi silencing showed increased Cd accumulation and reduced Zn and Mn concentrations in barley grains. Thus, *HvZIP3* and *HvZIP8* could be candidate genes related to low-grain-Cd-accumulation.

**Conclusion:**

Novel transporter genes such as *HvZIP3* and *HvZIP8* were identified as being associated with low-grain-Cd-accumulation. In addition to advancing academic knowledge, our findings may also result in potential economic benefits for molecular breeding of low Cd accumulating barley and other crops.

**Electronic supplementary material:**

The online version of this article (doi:10.1186/s12870-015-0648-5) contains supplementary material, which is available to authorized users.

## Background

As ubiquitous elements in nature, many heavy metals not only inhibit crop growth and reduce yields and quality but also pose a great threat to human health via food chain [1]. Cadmium (Cd) is non-essential for all living organisms and extremely toxic to humans in micro molar concentrations. Therefore, even healthy crops with very low level of Cd could be toxic to humans [2].

Many studies have been conducted to elucidate the underlying responses of plants to Cd stress and mechanisms conferring Cd tolerance [3]. Plants have developed two major strategies to resist Cd stress: “excluder” and “includer and tolerance” [4]. Under the “excluder” strategy, plants reduce the amount of Cd entering their roots and shoots. For instance, these plants can restrict Cd bioavailability from the soil, reduce the expression of transport proteins involve in Cd uptake, and increase the expression of membrane transporters that extrude Cd [5]. The second strategy is “includer and tolerance” strategy, which relies on compartmentation and detoxification of Cd in a controlled manner, allowing plants to accumulate metals to high concentrations (usually in vacuoles) without damaging normal functions of cells [6]. These Cd detoxification strategies rely on the combined activation of membrane transport and signal transduction pathways, which differ among plant species and among genotypes within a species [7].

Accordingly, gene identification and characterization are fundamental steps for deciphering the molecular mechanisms of plant Cd tolerance/accumulation for developing Cd-tolerant and low Cd accumulation transgenic crops [8]. Molecular approaches have been extensively applied to elucidate how plants respond to Cd toxicity [3]. DNA microarray technology has been employed to analyze the transcriptional responses of grey poplar under Cd stress [9]. Zhao et al. performed a comparative transcriptome analysis and found that Cd stress induced general and specific genes in *Arabidopsis thaliana* roots [10]. Quantitative RT-PCR is often used to validate the data obtained in microarray analyses. The gene expression profile (from DNA microarray analysis) of *Limonium bicolor* under salt stress were confirmed through qRT-PCR experiments [11].

As an RNA-dependent post-transcriptional gene-silencing technique, RNA interference (RNAi) represents an ideal alternative to gene disruption in many organisms [12]. Over the past ten years, transgene-induced RNAi had been developed as an efficient tool for the functional characterization of plant genes for crop improvement [13]. For instance, Cd accumulation was reduced by about 50 % in the rice grains of RNAi-*OsPCS1* (phytochelatin synthase gene) transgenic plants with no apparent difference of growth between RNAi and parental plants [14]. Zn- and Fe-regulated transporter-like protein (ZIP) gene family have been characterized and shown to be expressed through different growth stages of rice. *OsZIP1* and *OsZIP*3 seem to be important for Zn uptake from soil, *OsZIP4*, *OsZIP5* and *OsZIP8* for root to shoot translocation, while *OsZIP4* and *OsZIP8* could be particularly important for Zn transport to seed [15]. Cadmium translocation occur mainly via the same Ca^2+^, Zn^2+^, Fe^2+^, Mn^2+^ transporters [16], so the decrease of root-to-shoot metal translocation could be a major goal in cereals grown in Cd contaminated soils. Disruption of these transporter genes could prove their function and may suggest a strategy to limit Cd accumulation in crops.

Barley (*Hordeum vulgare* L.) is an established model crop for genetic and physiological studies on abiotic stress tolerance [17, 18]. In previous studies, we reported comprehensive investigation of physiological and cellular differences between Cd-tolerant and sensitive barley genotypes in response to Cd stress [19]. We demonstrated distinctive genotypic difference between low-grain-Cd-accumulating (W6nk2) and high-grain-Cd-accumulating (Zhenong8) genotypes in response to Cd stress [20]. However, our knowledge of candidate genes related to low grain Cd accumulation in these unique barley genotypes is still limited. Therefore, we hypothesize that there are large differences in genome-wide response to Cd stress and that the functions of some Cd-responsive genes are distinct between the two genotypes.

In this study, we employed multiple technologies, such as DNA microarray, qRT-PCR, RNAi, confocal microscopy, atomic absorption spectrometry, and gas exchange and chlorophyll fluorescence analyses to decipher the mechanisms underlying the differential expression and functions of Cd-induced genes in two barley genotypes. Our aims were to: (1) elucidate Cd distribution in different functional regions and cell types in roots; (2) identify Cd-induced differentially expressed genes responsible for low grain Cd accumulation and Cd tolerance; and (3) characterize the function of ZIP transporter genes (*HvZIP3* and *HvZIP8*) in RNAi transgenic barley lines. Our results provide significant insights into the complex mechanisms of Cd transport, accumulation, and tolerance in barley.

## Results

### Cd affects a range of physiological parameters in the two contrasting barley genotypes

Barley plants exposed to Cd treatments showed a significant decrease in plant height, root length, and shoot and root dry weight (Additional file 1: Figure S1). The symptoms of Cd toxicity differed significantly between low-grain-Cd-accumulating (W6nk2) and high-grain-Cd-accumulating (Zhenong8) genotypes with more severe growth reductions in the low-grain-Cd-accumulating genotype. In 50 μM Cd, for instance, shoot dry weight was reduced by 48.9 % in W6nk2 while it was only 14.6 % reduction in Zhenong8 (Additional file 1: Figure S1C).

After 15 days Cd exposure, maximal photochemical efficiency of PS II (*Fv/Fm*), photosynthetic quantum yield of photosystem II (ΦPSII) and coefficient of photochemical quenching (qP) had decreased significantly under increased Cd levels in W6nk2. However, the reduction of these parameters in Zhenong8 was less than in W6nk2 (Additional file 2: Figure S2A, B and C). Moreover, Cd stress induced a significant reduction in net photosynthetic rate (Pn), transpiration rate (Tr) and stomatal conductance (Gs) in both genotypes (Additional file 2: Figure S2E, F and G). In 500 μM Cd, Pn was completely inhibited in Cd-sensitive genotype W6nk2, while Zhenong8 reduced by 83.1 % compared with control (Additional file 2: Figure S2E). Leaf chlorophyll a and b contents were also significantly decreased in both genotypes under the three Cd treatments (Additional file 3: Figure S3) but the reduction of chlorophyll content was smaller in Zhenong8 (37.2 %) than in W6nk2 (48.6 %) under 50 μM Cd (Additional file 3: Figure S3A).

### The accumulation and distribution of Cd differ significantly between the two genotypes at the tissue and cellular levels

**Fig. 1:**
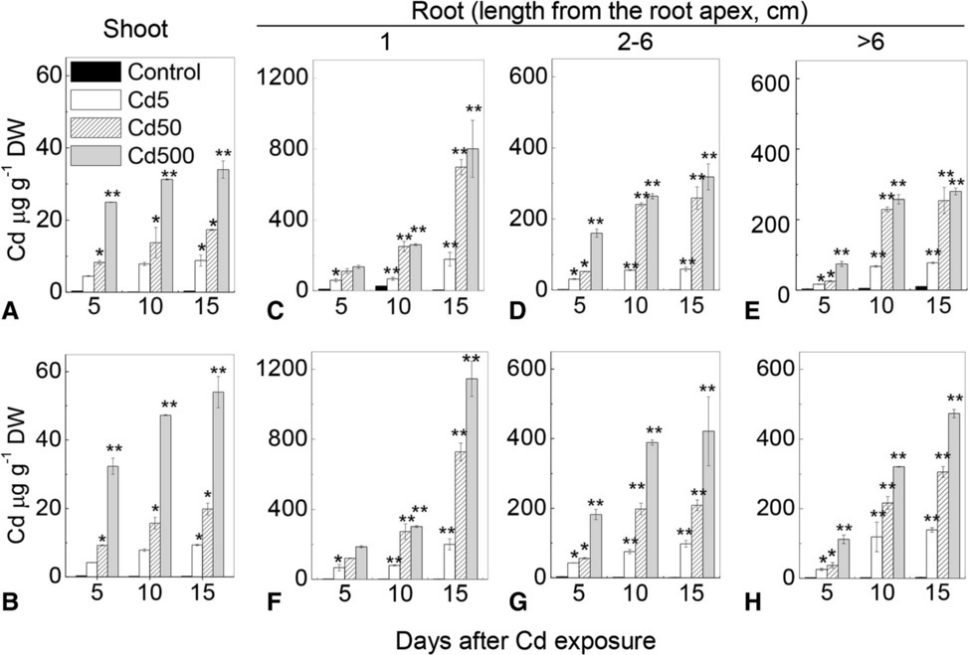
Effect of Cd levels and time of Cd exposure on Cd concentration in barley shoots and roots. The data are Cd concentration in shoots of W6nk2 (**a**) and Zhenong8 (**b**), and in different regions of roots of W6nk2 (**c**, **d**, and **e**) and Zhenong8 (**f**, **g**, and **h**). Cd concentration was measured after 5, 10 and 15 days of Cd treatments. Data are mean ± SD (*n* = 5). * and ** denote significant difference at *P* < 0.05 and *P* < 0.01 between Zhenong8 and W6nk2 at each of the four Cd levels over 15 days

Cd concentration increased proportional to the elevated external Cd levels, with up to 20-fold higher concentrations being observed in roots than the shoots (Fig. 1). There were significant genotypic differences in Cd concentrations detected in plants in relation to different Cd levels, tissues, and regions of roots, except for shoot Cd concentrations treated with 5 μM Cd after 5 and 10 days. After exposure to 50 μM and 500 μM Cd both shoot and root Cd concentrations in Zhenong8 were significantly higher than in W6nk2 (Fig. 1).

**Fig. 2:**
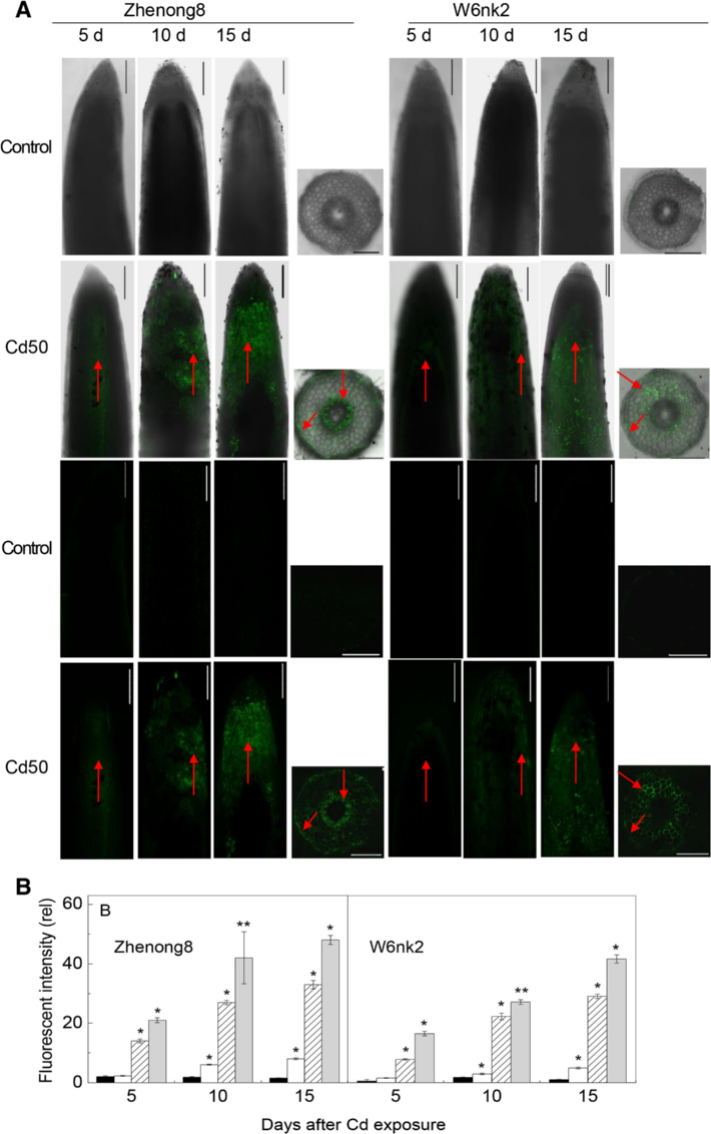
Localization of Cd^2+^ in barley roots exposed to Cd treatments. Representative micrographs show the binding of Cd^2+^ to Leadmium™ Green AM dye at longitudinal and cross sections of root tips from two barley genotypes Zhenong8 (*left panel*) and W6nk2 (*right panel*) after 15 days of Cd treatment **a** the first two lines are images from the green fluorescence indicate the fluorescent dye in Cd^2+^, the third and fourth are images of bright-field plus green flurescence. Roots were exposed to 0 and 50 μM Cd for 5, 10 and 15 days before staining. Scale bars = 250 μm. **b** Relative Cd^2+^ fluorescence density from longitudinal section of root tips. Black, white, shaded and grey bars represent 0, 5, 50, 500 μM Cd, respectively. Data are means ± SD (*n* = 5). * and ** denote significant difference at *P* < 0.05 and *P* < 0.01, respectively, between Zhenong8 and W6nk2 at each of the four Cd levels over 15 days. Arrows denote specific differences between two genotypes

Confocal imaging experiments confirmed the above results, demonstrating significantly higher Cd accumulation in the 1-cm root tips of Zhenong8 plants in comparison to W6nk2 (Fig. 2). The preferential location of Cd in roots of Zhenong8 plants treated with 50 μM Cd after 5 days was in the root apex, and this effect was more pronounced under increased Cd concentrations and exposure times (Fig. 2a and b). On the contrary, there was no Cd detected by the Cd-sensitive fluorescent probe (Leadmium™ Green) in the root tips of W6nk2 plants after 5 days of 50 μM Cd treatment. Cross-sectional images of roots revealed that most Cd accumulated in inner epidermis and endodermis. When the two genotypes were compared, there was more green fluorescence observed in endodermis and cortex of W6nk2, while there was stronger green fluorescence in inner epidermis and stellar cells of Zhenong8 (Fig. 2a).

### Cd stress results in large-scale changes in gene expression

The physiological distinctions observed in response to Cd stress (Figs. 1 and 2, Additional file 1: Figure S1, Additional file 2: Figure S2 and Additional file 3: Figure S3) between the two unique barley genotypes led to further exploitation of their differences at the transcriptome level. Overall, we found that compared to the control plants, the gene expression profiles the leaves of two genotypes were significantly altered after 15 days in 5 μM Cd. Cadmium stress induced differential expression of 812 genes. Among these genes, 382 (131) and 303 (106) were up-regulated (down-regulated) in W6nk2 and Zhenong8, respectively, with a fold change of at least 2.0 (*P* ≤ 0.05). These genes represented 2.3 and 1.8 % of the total genes in the two genotypes (data not shown). There were distinct differences in gene expression between the two genotypes (Additional file 4: Figure S4, Additional file 5: Figure S5 and Fig. 5; Table 1, Additional file 6: Table S1, Additional file 7: Table S2, Additional file 8: Table S3 and Additional file 9: Table S4), with 338 genes being up-regulated in W6nk2 (W-*up*) but unchanged in Zhenong8 (Z-*NC*), while another 13 genes were up-regulated in W6nk2 (W-*up*), but down-regulated in Zhenong8 (Z-*down*) (Additional file 4: Figure S4A).

**Table 1:** Membrane transport related genes up-regulated in W6nk2 and down-regulated or no change in Zhenong8 after 15 days exposure to 5 μM Cd

Annotation	Probe ID	Fold change (Cd treatment *vs* control)		Accession no	E-value
		W6nk2	Zhenong8		
Zinc transporter 11 [*A. thaliana*]	Contig11777_at	3.75	−1.01	AAF79317.1	7e^−40^
Putative zinc transporter protein ZIP1 [*O. sativa* (japonica)]	Contig16352_at	2.05	1.06	BAC21508.1	3e^−47^
ZIP-like zinc transporter [*T. caerulescens*]	HD12H12r_at	2.13	1.50	AAK69429.1	2e^−51^
C2H2 zinc finger protein [*O. sativa*]	Contig14114_at	2.89	−1.08	AAL76091.1	2e^−12^
ABC transporter family protein [*A. thaliana*]	Contig26036_at	2.96	1.14	NP_200978.1	6e^−31^
Putative ABC transporter [*O. sativa* (japonica)]	Contig12753_at	4.14	−1.46	BAB93292.1	4e^−75^
Putative ABC transporter [*A. thaliana*]	Contig21659_s_at	2.87	−1.33	AAF98206.1	1e^−22^
Putative MRP-like ABC transporter [*O. sativa* (japonica)]	Contig9422_at	2.38	−1.84	BAB62557.1	1e^−102^
P-type ATPase [*H. vulgare*]	Contig14075_at	2.11	−1.21	CAC40030.1	1e^−93^
P-type ATPase [*H. vulgare*]	Contig14715_at	2.03	1.28	CAC40028.1	1e^−18^
Iron-phytosiderophore transporter protein yellow stripe 1 [*Z. mays*]	HV_CEa0013E09r2_at	3.09	1.50	AAG17016.2	2e^−33^
Iron-phytosiderophore transporter protein yellow stripe 1 [*Z. mays*]	Contig16464_at	2.08	1.30	AAG17016.2	2e^−95^
Putative potasium transporter [*O. sativa* (japonica)]	Contig18758_at	4.65	1.37	CAD20994.1	1e^−94^
Putative phosphate translocator [*O. sativa* (japonica)]	Contig20673_at	2.14	−1.27	AAK21346.1	4e^−78^
Putative ammonium transporter [*O. sativa* (japonica)]	Contig22563_at	4.73	−1.49	BAB64105.1	1e^−100^
Monosaccharide transporter 3 [*O. sativa*]	Contig5537_at	2.94	−1.24	BAB19864.1	2e^−95^
Similar to hexose carrier protein [*O. sativa* (japonica)]	HV_CEa001e24r2_s_at	2.08	−1.15	BAA83554.1	2e^−36^
Sugar transport protein 13 [*A. thaliana*]	Contig9662_at	2.07	1.22	NP_198006.1	2e^−81^
Peptide transporter [*A. thaliana*]	Contig12317_at	2.47	1.52	NP_177024.1	2e^−49^
Putative phosphoribosylanthranilatetransferase [*O. sativa*]	Contig5883_s_at	2.26	−2.44	AAM19104.1	4e^−35^
Putative flavonol 3-sulfotransferase [*O. sativa* (japonica)]	Contig12075_at	2.81	−1.24	AAN04969.1	6e^−34^
Nonspecific lipid-transfer protein precursor [*Malusdomestica*]	Contig12237_at	5.33	−1.11	Q9M5X7	2e^−09^
Putative lipid transfer protein [*O. sativa* (japonica)]	Contig4414_at	2.53	1.67	AAN05565.1	3e^−25^
Putative transport protein SEC61 beta-subunit [*A. thaliana*]	HM02O03r_s_at	2.10	−1.08	NP_182033.1	7e^−05^
Fructosyltransferase [*L. perenne*]	rbah48h06_s_at	2.29	1.64	AAL92880.1	6e^−84^
Glycoprotein glucosyltransferase [*A. thaliana*]	Contig8758_at	2.20	1.09	NP_177278.1	9e^−66^
Putative anthranilate N-benzoyltransferase [*O. sativa* (japonica)]	Contig15413_at	2.73	1.48	AAM74310.1	1e^−27^
Glutathione transferase F3 [*T. aestivum*]	HW09A20u_at	2.13	−2.12	CAD29476.1	1e^−09^
Putative glutathione S-transferase [*O. sativa* (japonica)]	Contig21026_at	2.19	−1.70	BAB39941.1	1e^−66^
Glutathione transferase [*T. aestivum*]	Contig12776_at	2.53	−1.65	CAC94004.1	2e^−62^
Putative glutathione S-transferase [*O. sativa*]	Contig4044_at	2.22	−1.26	AAK38509.1	3e^−68^
Glutathione S-transferase GST 22 [*Z. mays*]	Contig9632_at	2.90	1.57	AAG34830.1	2e^−60^
Putative glutathione S-transferase [*O. sativa* (japonica)]	Contig6333_at	2.50	1.04	AAN05495.1	1e^−63^
Glutathione transferase F5 [*T. aestivum*]	Contig2456_at	2.36	1.01	CAD29478.1	1e-^108^
Calreticulin [*H. vulgare*]	rbags16g09_s_at	2.60	−1.03	T05705	2e^−18^
Putative Ras-related protein Rab [*O. sativa*]	Contig8562_at	2.05	1.02	AAM08543.1	9e^−91^
Germin-like protein [*H. vulgare*]	Contig3156_s_at	3.11	1.03	T05956	7e^−36^
Probable oxalate oxidase [*O. sativa*]	Contig10860_at	4.86	1.20	T02923	2e^−41^
Rabphilin-3A [*Bostaurus*]	Contig24950_at	2.25	1.44	Q06846	5e^−3^
Contains ESTs C74435 ~ similar to nodulin [*O. sativa* (japonica)]	Contig1402_at	3.64	−1.35	BAC20892.1	1e^−70^
Putative lipid transfer protein [*O. sativa*]	Contig3776_s_at	1.49	−2.2	AAM74427.1	2e^−18^
Putative phosphoserine aminotransferase [*O. sativa*]	Contig5879_at	1.14	−2.08	AAM51827.1	e^−115^
Putative o-methyltransferase ZRP4 [*O. sativa*]	Contig8812_x_at	−1.73	−3.47	AAL31649.1	e^−61^
Putative dolichyl-phosphate mannosyltransferase [*A. thaliana*]	Contig17479_at	−1.27	−2.2	NP_177574.1	3^e-27^
Putative alanine acetyl transferase [*A. thaliana*]	Contig15462_at	1.4	−2.44	NP_180763.1	6^e-27^

The differentially regulated Cd-responsive genes were clustered into 4 categories according to the similarities in their expression profiles (Additional file 5: Figure S5; Tables 1, Additional file 6: Table S1, Additional file 7: Table S2, Additional file 8: Table S3 and Additional file 9: Table S4). Among the genes that were up-regulated in the low-grain-Cd-accumulating genotype W6nk2 but down-regulated or unchanged in Zhenong8, or no change in W6nk2 but down-regulated in Zhenong8, 27 % were related to stress and defense responses and 11 % represented functions related to transport (Additional file 5: Figure S5A). The genes that were up-regulated in the high-grain-Cd-accumulating genotype Zhenong8 but down-regulated or unchanged in W6nk2, or unchanged in Zhenong8 but down-regulated in W6nk2, except for unknown classified and none function sections the largest portion with 15 % was related to photosynthesis, followed by transcription with 13 % (Additional file 5: Figure S5B). The genes that were up-regulated in both genotypes were mainly involved in stress and defense responses (23 %) and carbohydrate metabolism (19 %) except for category with unknown function (none) (Additional file 5: Figure S5C). Furthermore, the genes that were down-regulated in both genotypes mainly involved in unknown classified and none function sections, followed by photosynthesis (up to 9 %), stress and defense responses equal to transcription and transport (up to 5 %), meanwhile, there were a small amount genes related to protein synthesis, signal transduction and so on (Additional file 5: Figure S5D).

### Cd stress leads to higher expression of genes involved in transport, stress and defense responses, carbohydrate metabolism and signal transduction in the low-grain-Cd-accumulating genotype

**Fig. 3:**
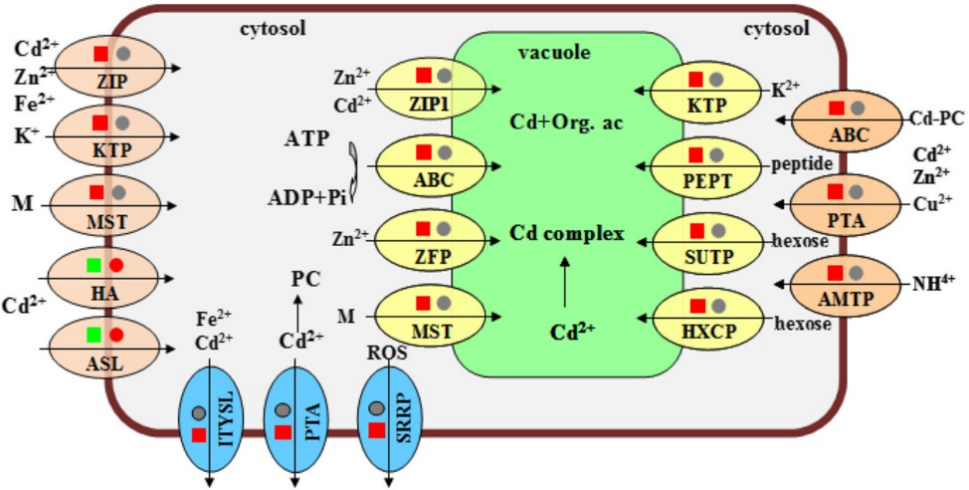
Schematic diagram of membrane transporter genes involved in low Cd accumulation in barley leaves. Some representative genes encoding membrane transport proteins transport Cd and other ions on the plasma membrane and tonoplast in W6nk2 and Zhenong8. Squares and circles represent transporter genes expressed in W6nk2 and Zhenong8, respectively. Red, grey and green symbols indicate Cd-induced up-regulation, no change and down-regulation of the transporter genes in comparison to the control, respectively. ABC, ABC transporter family protein; AMTP, Ammonium transporter; ASL, ATP sulfurylase; HA, H^+^-ATPase; HXCP, Hexose carrier protein; ITYSL, Iron transporter yellow stripe 1-like; KTP, Potassium transporter; MST, monosaccharide (M) transporter; Org. ac., organic acids; PC, phytochelatin; PEPT, peptide transporter; PTA, P-type ATPase; ROS, reactive oxygen species; SRRP, Stripe rust resistance protein; SUTP, Sugar transport protein; ZFP, Zinc finger protein; ZIP, Zinc transporter protein

**Fig. 4:**
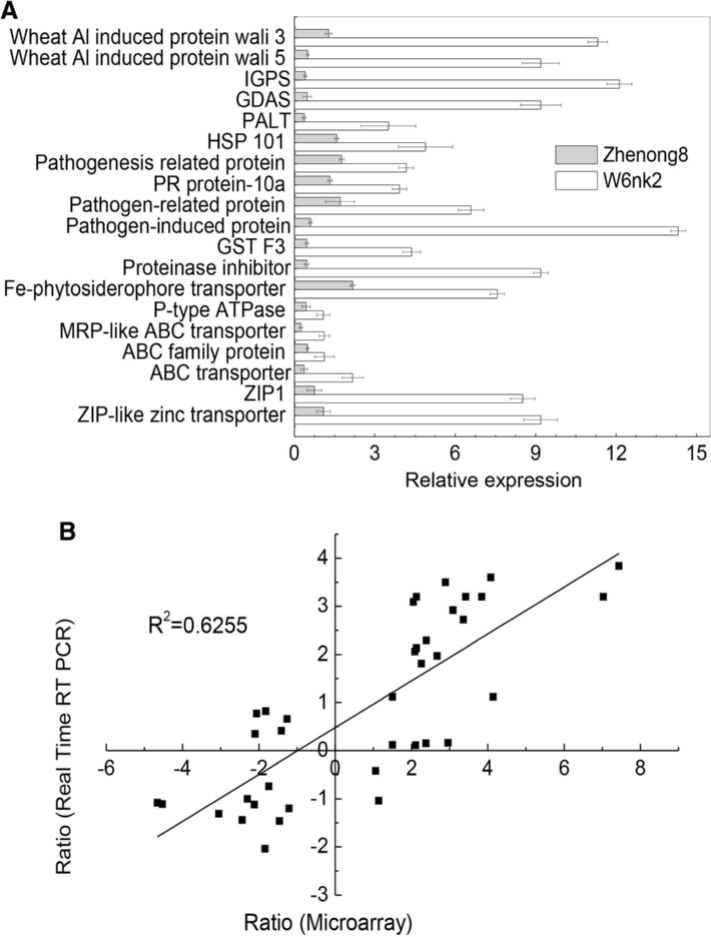
Quantitative RT-PCR validations on microarray data of Cd-responsive transporter genes in barley leaves. **a** The values represent the gene expression in 5 μM Cd over those in the control in Zhenong8 (*grey bars*) and W6nk2 (*white bars*) (*n* = 3 biological replicates). ACT2 was used as the reference gene. Data plotted are the mean ratio of gene expression in Cd treatment (5 μM Cd for 15 days) over those in the control in the two genotypes on a log_2_ scale (**b**). GDAS, Glutamine-dependent asparagine synthetise; PALT, Phosphoribosylanthranilate transferase; IGPS, Indole-3-glycerol phosphate synthase; HSP, Heat shock protein

**Fig. 5:**
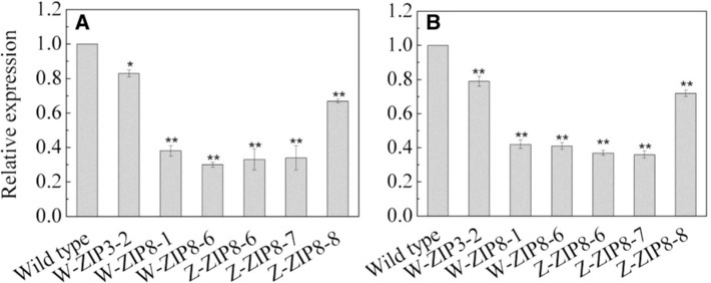
Relative expression level of ZIP in ZIP-RNAi lines compared to the Golden Promise parent. W-ZIP3, transgenic plants with *HvZIP3* RNAi in W6nk2; W-ZIP8, transgenic *HvZIP8* RNAi in W6nk2; Z-ZIP8, transgenic *HvZIP8* RNAi of Zhenong8. **a** T0 lines; **b** T1 lines. * and ** indicates significant difference between transgenic lines and the parental line at *P* < 0.05 and *P* < 0.01, respectively

The most relevant group related to low grain Cd accumulation were the genes up-regulated in Cd-sensitive W6nk2 and down-regulated or unchanged in Cd-tolerant Zhenong8 plants, or unchanged in W6nk2 but down-regulated in Zhenong8 (Figs. 3, 4 and 5, Additional file 5: Figure S5A and Additional file 10: Figure S6; Tables 1 and Additional file 6: Table S1). Among them, category 1 contained 45 transport related genes (Fig. 5, Table 1). A closer inspection of the genes involved in transport revealed that they were expressed at a significantly higher level in W6nk2 than in Zhenong8 under Cd treatment. For instance, Cd-induced fold changes in the transcript levels of *zinc transporter 11* and a *putative ABC transporter* were 3.75 and 4.14, respectively, in W6nk2, whereas they were −1.01 and −1.46 in Zhenong8. Moreover, the transcripts of two macronutrient transporters (a potassium transporter and an ammonium transporter) were highly expressed in W6nk2 under Cd stress, showing 4.65 and 4.73 fold changes, respectively (Table 1). Category 2 contained 106 stress and defense response-related genes, others were 43 carbohydrate metabolism-related genes, and 35 signal transduction-related genes (Additional file 6: Table S1 and Additional file 10: Figure S6).

### Cd stress leads to higher expression of genes involved in photosynthesis, protein synthesis and transcription in the high-grain-Cd-accumulating genotype

Interestingly, we found that genes that were higher expressed in Cd-tolerant Zhenong8 plants were very different from those in W6nk2 (Additional file 5: Figure S5B, and Additional file 11: Figure S7; Additional file 7: Table S2). They included 337 genes that were up-regulated in Zhenong8 and down-regulated or no change in W6nk2, or unchanged in Zhenong8 and down-regulated in W6nk2. The numbers of these genes involved in different processes as follows: photosynthesis (51), transcription (43), protein synthesis (39), transport (32), and stress and defense responses (26) (Additional file 5: Figure S5B). The Cd-induced expression of some key genes was many fold higher under Cd treatment *vs.* the control in Zhenong8 than in W6nk2 (Additional file 7: Table S2). For examples the changes of genes expression level encoding proteins such as plastid-specific ribosomal protein 2 precursor were 6.48 folds, ribonucleo protein 7.24 folds, rubisco large subunit-binding protein beta subunit 20.53 folds, lipid transfer protein 7.33 folds and adenosine diphosphate glucose pyrophosphatase 14.55 folds. We assumed that they could be related to Cd accumulation.

There were 31 genes up-regulated in both W6nk2 and Zhenong8 after 15 days exposure to 5 μM Cd, of which 23 % were related to stress and defense response, 19 % to carbohydrate metabolism (Additional file 5: Figure S5C, Additional file 8: Table S3). Aside, 43 genes were down-regulated in both W6nk2 and Zhenong8 after Cd exposure, however 72 % of them were unknown classified (none) (Additional file 5: Figure S5D and Additional file 9: Table S4).

### The differential expression of transporter genes are verified via qRT-PCR

To verify the microarray data, we selected 30 representative genes from different gene categories. The primers targeting these genes are listed in Additional file 12: Table S5. Expression analysis of selected genes *via* quantitative real time PCR (qRT-PCR) confirmed the transcript levels observed in DNA microarray (Fig. 4). Nineteen representative genes (e.g., genes encoding an ABC transporter, chitinase, and glutathione transferase) showed consistently higher expression levels in W6nk2 compared to Zhenong8. For instance, the average relative expression of two *ZIP* genes in W6nk2 was approximately 10 times higher than in Zhenong8 after Cd treatment (Fig. 4a).

### Silencing *HvZIPs* leads to significantly higher accumulation of Cd and a reduction of Zn and Mn in barley grains

As *ZIP* genes are key transporters of toxic Cd and the essential minerals Zn and Mn, we utilized RNAi technology to further investigate whether disruption of these genes at mRNA level affects Cd and mineral accumulation in barley grains. In this experiment, a highly conserved portion of the *HvZIP* gene was cloned into the pSTARGATE vector (supplied by CSIRO Plant Industry, Australia; http://www.pi.csiro.au/RNAi/vectors.htm) (Additional file 13: Figure S8). We made three RNAi constructs, harboring *HvZIP3* and *HvZIP8* isolated from W6nk2 and *HvZIP8* isolated from Zhenong8 (Additional file 13: Figure S8, Additional file 14: Figure S9) and transgenic lines were generated. Out of 400 transformed immature barley embryos, 28 transgenic lines were regenerated and 6 transgenic barley lines showed the presence of *HvZIP3* and *HvZIP8* antisense sequences detected *via* PCR.

The expression of the antisense transcripts from the *HvZIP* RNAi constructs in the transgenic barley primary transformants (T0) and the next generation (T1) lines were evaluated and compared to that in the parental *cv.* Golden Promise using qRT-PCR (Fig. 5). The relative expression in *cv.* Golden Promise was set to 1 as a reference for estimation of the relative expression level of *HvZIP3* or *HvZIP8* in the RNAi lines. The relative expression levels in the RNAi lines were 1.2 to 3.3-fold lower than in the parental plants, and the W6nk2 ZIP3-2 and Zhenong8 ZIP8-8 lines showed the highest transcript levels of *HvZIP3*and *HvZIP8*, respectively (Fig. 5a). The relative expression of *ZIP* in next generation (T1) lines revealed similar results as was observed in the primary transformants (Fig. 5b).

**Fig. 6:**
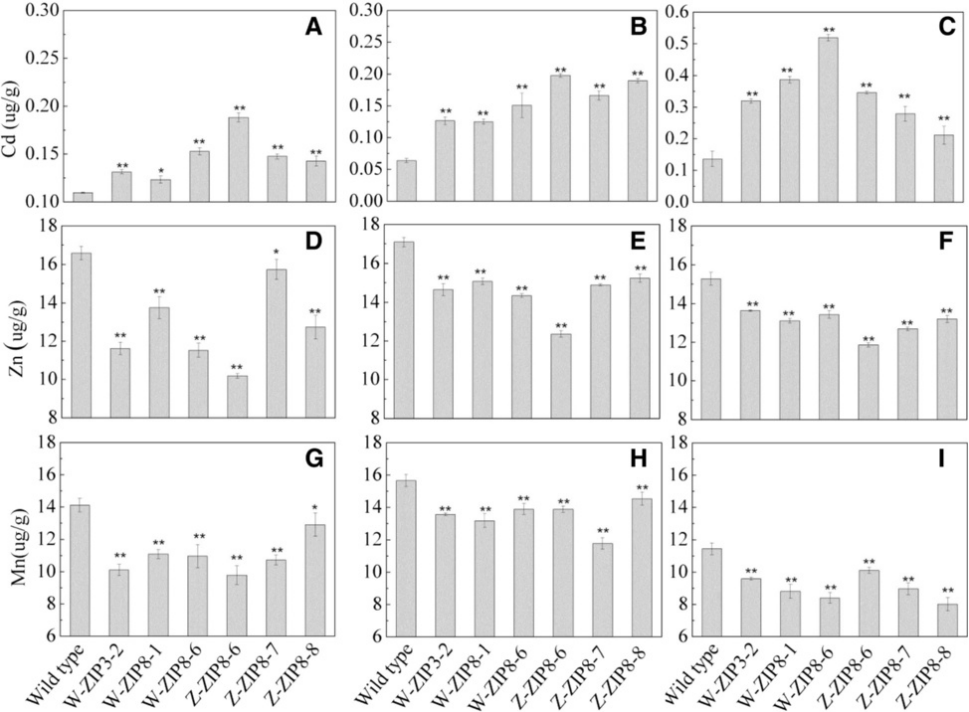
Concentrations of Cd, Zn and Mn in grains of different ZIP-RNAi lines and the parental barley under normal condition and Cd treatment. *W-ZIP3*, transgenic plants with *HvZIP3* RNAi in W6nk2; *W-ZIP8*, transgenic *HvZIP8* RNAi in W6nk2; *Z-ZIP8*, transgenic *HvZIP8* RNAi of Zhenong8. Different number represents transgenic lines. Data are mean ± SD (*n* = 3). * and ** indicates significant difference between transgenic lines and the parental line at *P* < 0.05 and *P* < 0.01, respectively. The ZIP-RNAi lines in left panel (**a**, **d** and **g**) are T1 grains without Cd; the ones in middle panel (**b**, **e** and **h**) are T2 grains under normal condition; the ones in right panel (**c**, **f** and **i**) are T2 grains under Cd treatment with artificial contamination of 5 mg kg^−1^ Cd in soil

The levels of Cd and two related microelements, Zn and Mn, were then determined in parental barley and the T1 RNAi grains (Fig. 6a, d and g). The Cd concentrations in all of the RNAi grains were significantly increased, by up to 70 % compared to parental *cv.* Golden Promise plants. In addition, the Cd level in the ZIP3 line was much lower than in the six ZIP8 lines excepted for W6nk2-ZIP8-1 line. In contrast, the silencing of *HvZIP*s resulted in a significant decrease in Zn and Mn concentrations in all of the T1 RNAi grains, with the exception of the Zn concentration in the Zhenong ZIP8-7 line (Fig. 6d and g). Examination of Cd concentration in T2 transgenic grains (Fig. 6b, c, e, f, h and i) have conformed this results. Both under control and Cd added conditions, concentrations of Cd in grains of transgenic lines were significantly higher than that in parental line. Similarly, the concentrations of Zn and Mn in grains of transgenic lines showed a significant reduction compared to the parental line. In addition, the Cd concentration in all of the T2 transgenic grains under Cd treatment was significantly higher than that under normal condition (Fig. 6b and c). However, the Zn and Mn concentrations under Cd treatment were lower than under normal condition (Fig. 6e, f, h and i).

### Silencing *HvZIPs* leads to change of grain ultrastructure

**Fig. 7:**
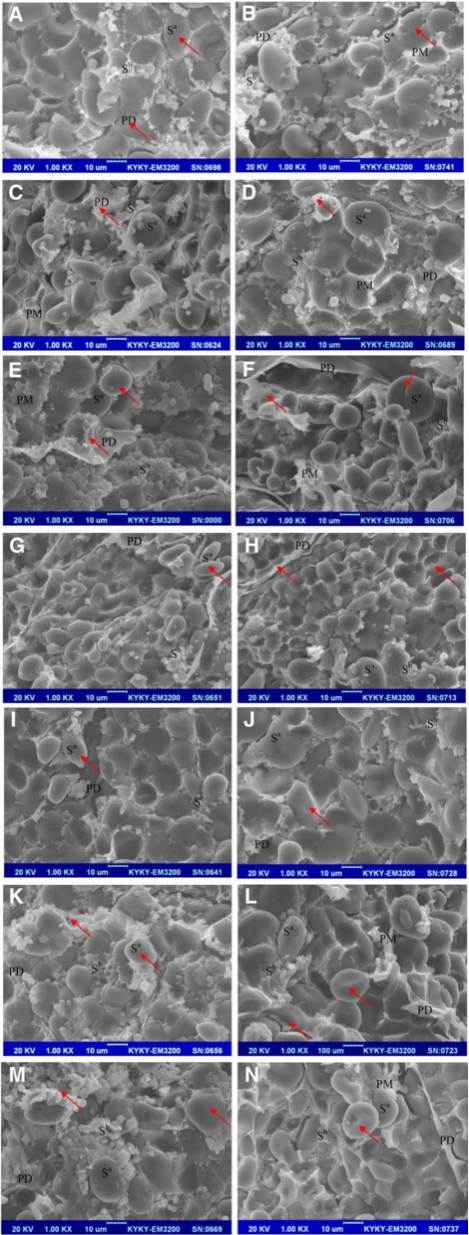
Scanning electron microscopy (SEM) image of matured grains. The SEM images illustrate the starch–protein interface in the parental line (**a** and **b**) compared to the T2 transgenic grains (**a** and **b**, WT Golden Promise; **c** and **d**, W-ZIP3-2; **e** and **f**, W-ZIP8-1; **g** and **h**, W-ZIP8-6; **i** and **j**, Z-ZIP8-6; **k** and **l**, Z-ZIP8-7; **m** and **n**, Z-ZIP8-8). The ones in left panel (**a**, **c**, **e**, **g**, **i**, **k** and **m**) are grains under normal condition, and the ones in right panel (**b**, **d**, **f**, **h**, **j**, **l** and **n**) are grains under Cd treatment with artificial contamination of 5 mg kg^−1^ Cd in soil. Note the higher amount of associated protein in transgenic lines, which surrounds the large A type starch granules (S^a^) and engulfs the smaller B-type starch granules (S^b^). PD, protein deposits; PM, protein matrix among large and small starch granules. Arrows denote specific differences between parental line and transgenic grains or between control and Cd treatment

Scanning electron microscopy (SEM) observations showed that the central endosperm of barley grains that were exposed to Cd was packed with A- and B-type starch granules with some protein matrix that surrounded the large A-type starch granules and engulfed the smaller B-type starch granules (Fig. 7). Compared with the parental cultivar, when exposed to Cd, A-type granules in transgenic lines exhibited much more serious surface erosion or deformation. Furthermore, the proportion of B-type starch granule was lower in transgenic lines than that in parental cultivar. It was noted that some large starch-associated proteins accumulated around the starch granules of transgenic lines, while very few were observed in wild type. Additionally, it was noted that some large protein-rich deposits accumulated around the starch granules of transgenic lines. In contrast, very few deposits were observed in Golden Promise.

## Discussion

### Can we identify low-grain-Cd-accumulating and Cd-tolerant barley genotypes?

Cadmium contamination remains a potential threat to human health due to its long biological half-life and easy uptake by plants. Overall Cd tolerance but low Cd accumulation in grains are important characteristics in cereals and considered as two key traits related to Cd toxicity [7]. However, as it was reported previously and reinforced in the present study, these two traits do not always co-exist in a single species or genotype [20, 21].

Cd stress caused a significant decrease in growth, photosynthesis, pigment levels and yields in both studied genotypes, with W6nk2 being more strongly affected (Additional file 1: Figure S1, Additional file 2: Figure S2 and Additional file 3: Figure S3). In addition, Cd-tolerant Zhenong8 showed significantly higher Cd accumulation in shoots and various regions of roots than W6nk2 (Figs. 1 and 2). These results were in accord with our previous studies [20]. Therefore, our comprehensive transcriptome analysis of these contrasting genotypes provided insights into the molecular mechanisms and genes responsible for the Cd tolerance but low Cd accumulation traits in barley.

Compared to other crop species (e.g. rice and wheat), barley (especially wild barley) possesses high genetic diversity [22] and shows higher tolerance to many abiotic stresses, such as drought [23], salinity [24], and acidic soils [25]. The work on Tibetan wild barley has shown some promising results regarding the identification of low-grain-Cd-accumulating and Cd-tolerant genotypes [24]. Seventy percent of 188 examined Tibetan wild barley lines are more salt-tolerant than the well-known salt tolerant elite cultivar CM72. Most of these highly salt-tolerant lines accumulate significantly less Na^+^ than the cultivated barley genotypes [24]. If similar mechanisms also apply to Cd tolerance, it is our interest to further investigate these wild lines for potential use in breeding to obtain low-grain-Cd-accumulating and Cd-tolerant genotypes.

### Comparative transcriptome analysis reveals key transporter genes associated with low Cd accumulation

To our knowledge, no detailed molecular evaluation of low Cd accumulation has previously been conducted in barley. In this study, many genes were differentially induced in W6nk2 and Zhenong8 in response to Cd stress (Additional file 10: Figure S6; Table 1, Additional file 6: Table S1, Additional file 7: Table S2, Additional file 8: Table S3 and Additional file 9: Table S4). Based on the identified Cd-responsive transporter genes, we were able to produce an integrated schematic diagram of the potential mechanisms involved in low Cd accumulation (Fig. 3). This has provided some novel clues to the molecular mechanisms underlying low Cd accumulation in barley.

Our previous studies demonstrated that Zn interferes with Cd uptake and translocation and significantly alleviates Cd stress in barley [21]. At the molecular level, the toxicity of Cd to plants is generally finely regulated by many membrane transporters [7, 26]. In this study, genes encoding more than 20 of these membrane transporters, including three members of the ZIP transporter family: zinc transporter 11, the zinc transporter ZIP1, and a ZIP-like zinc transporter, were found to be highly expressed in W6nk2 plants under Cd stress (Table 1). Hence, the ZIP transporters are crucial for the transport of both essential micronutrients (Zn, Fe, Mn and Cu) and non-essential heavy metals such as Cd [27–31]. Recently, Milner et al. found that 6 out of the 11 Arabidopsis ZIP family members complement a yeast Zn uptake-deficient mutant. *AtZIP1* and *AtZIP2* are Zn and Mn transporters present at the root stele tonoplast and plasma membrane, respectively [31]. Our results in agreement with these findings and showed that the levels of Cd, Zn and Mn are significantly affected in ZIP-RNAi barley lines (Fig. 6). In addition, ATP-binding cassette (ABC) transporter family is responsible for the transport of heavy metals, xenobiotics and lipids, and its members are involved in the vacuolar sequestration, distribution and homeostasis of Cd in plants [32, 33]. For example, disruption of *AtMRP5* leads to changes in guard cell anion levels and calcium channel activity in Arabidopsis [34]. In this study, the transcripts encoding four ABC transporters were significantly highly expressed in W6nk2 than in Zhenong8 (Table 1). We therefore propose that W6nk2 might be more efficient in vacuolar Cd sequestration and Cd extrusion from cells in the shoots and roots, resulting in lower Cd accumulation in barley grain compared to Zhenong8.

Tightly regulated transporters of essential nutrients such as ammonium, K, Fe and phosphate and pH homeostasis are essential for plant performance under Cd stress. Among the eight P-type ATPases (or *HMAs*) found in Arabidopsis, *HMA2* and *HMA4* are essential for the transport and root-to-shoot translocation of Zn and Cd [35, 36]. In this study, the observed Cd-induced up-regulation of genes encoding two P-type ATPases in W6nk2 may indicate its efficiency in reducing grain Cd concentration (Table 1). Moreover, we detected Cd-induced up-regulation of genes encoding iron transporter yellow stripe 1-like (ITYSL), a potassium transporter (KTP), an ammonium transporter (AMTP) and a phosphate translocator only in W6nk2 (Table 1). Under Cd stress, nitrogen can be recycled and translocated from the shoots to roots as a Cd protection and storage strategy, with ammonium representing the preferred form of nitrogen [37]. A recent publication shows that the Arabidopsis nitrate transporter NRT1.8 functions in the removal of nitrate from the xylem sap and mediates cadmium tolerance [38]. Plasma membrane *KTPs* contribute to cellular homeostasis and excitability in higher plants [39]. A significantly negative relationship has been discovered between Fe and Cd concentrations [1, 40], and iron deficiency stimulates heavy metal transport in plants [40, 41]. It has been suggested that *yellow stripe 1* (*HvYS1*) and *ZmYS1* play unique roles in delivering a broad range of essential metals in barley and maize [42, 43].

In summary, the Cd-induced up-regulation of these transporters may contribute to the low grain Cd accumulation observed in W6nk2 through the direct transport of Cd *via* ZIP and ABC transporters and through indirect regulation of ammonium, K, Fe, phosphate and pH homeostasis. In the low-grain-Cd-accumulating genotype (W6nk2) hence the up-regulation of these transporters may contribute to the low grain Cd accumulation perhaps by limiting the export to of Cd being accumulated in vegetative tissues and less in the grains. From the other hand the down-regulation of the same transporters in the high-grain-Cd-accumulating genotype (Zhenong8) could have led to the Cd to be exported to the grains.

### Cd-tolerant genotype utilizes different strategies at the transcriptome level for combating Cd stress

In the present work, many unique genes were found to be up-regulated in Cd-tolerant genotype Zhenong8 in response to Cd stress (Additional file 10: Figure S6; Additional file 7: Table S2). Based on the identified Cd-responsive genes, an integrated schematic diagram of the mechanisms related to Cd tolerance was proposed (Additional file 11: Figure S7). This integrated schematic diagram of Cd-tolerance-related genes may provide novel insights into the molecular mechanisms underlying Cd tolerance in barley, in contrast to the mechanisms that are relevant to low grain Cd accumulation. The Cd-tolerant genotype Zhenong8 showed several strategies that were very different from those observed in W6nk2 but were effective in altering its transcriptome in response to Cd stress.

Twenty-nine photosynthesis-related genes were found to be up-regulated in Zhenong8 but unchanged or down-regulated in W6nk2 under Cd treatment (Additional file 7: Table S2) and the up-regulation of these genes was translated into 6-fold higher net CO_2_ assimilation (Additional file 2: Figure S2). The negative impact of Cd on photosynthesis is well documented [44]. As the most abundant protein in plants, rubisco catalyzes the first step in CO_2_ assimilation and photo respiratory carbon oxidation [45]. Here, we identified candidate genes encoding large and small subunits of rubisco that were up-regulated, showing up to 20-fold changes, in Cd-tolerant Zhenong8 plants under Cd treatment. Ferredoxin NADP(H) oxidoreductases catalyze electron transfer between NADP(H) and ferredoxin. Knockout of *AtLFNR1* decreases the contents of chlorophyll and light-harvesting complex proteins and markedly reduces the PSI/PSII ratio in the mutant compared to wild-type plants [46]. Additionally, other genes, encoding NADPH-protochlorophyllide oxidoreductase B, PsbP and PsbP-related thylakoid lumenal proteins, may also play key roles in the improvement of photosynthesis in Zhenong8 under Cd treatment.

Many stress defense signalling related genes encoding jasmonate-(JA) and ethylene-(ET) forming-enzyme-like proteins and calcium-dependent protein kinases were found to be up-regulated in Zhenong8. These genes have been well characterized regarding the roles in abiotic stress tolerance [29, 47]. JA, ET and Ca signals play significant roles in the adaption of plants under various stresses, both independently and through synergistic and antagonistic cross-talk [47]. Cd stress triggers the accumulation of JA and ET in Arabidopsis and beans, respectively [48]. Fuhrer et al. reported that under Cd-induced stress, ethylene restricts the water and Cd flux into bean leaves [48]. Additionally, the Ca^2+^-binding protein calmodulin (CAM) transduces second messengers in cellular responses [49]. Cd may enter cells via Ca^2+^-selective channels or specific transmembrane transporters to compete with Ca^2+^ for CAM-binding sites, resulting in a reduced ability of plants to sense stress signals [50]. Our results indicated that Cd induced a greater increase in the expression of genes related to stress defense signaling in Zhenong8 compared to W6nk2, which could contribute the Cd tolerance of Zhenong8.

Among the 23 protein synthesis genes that were up-regulated in Zhenong8 and down-regulated in W6nk2 in response to Cd 5 encoded ribosomal proteins and their precursors. Protein synthesis is critical for abiotic stress tolerance in plants [51]. Pytharopouloua et al. suggested that under Cd^2+^-mediated stress, protein-synthesizing activity is reorganized both quantitatively and qualitatively, and Cu^2+^ causes a progressive loss of the ability of 40S-ribosomal subunits to form the 48S pre-initiation complex, decreasing to 34 % of the control level at the end of exposure [52]. Plastid ribosomal proteins (PRPs) are essential for ribosome biogenesis, plastid protein biosynthesis, chloroplast differentiation, and early chloroplast development, and nuclear-encoded PRPS20 plays an important role in chloroplast development in rice [53]. PRPs are crucial for the establishment of the transcription/translation apparatus during the build-up step in chloroplast differentiation in maize [54]. A lack of PRPs has diverse phenotypic effects in plants, including decreased photosynthetic capacity, reduced plant height or even lethality [55]. Our finding suggested that ribosomal proteins are crucial for Cd tolerance in barley as well.

Finally, in this study, H^+^-ATPase, ATP synthase and ATP-sulfurylase encoded by detoxification-related genes were found to be up-regulated in Zhenong8 but down-regulated in W6nk2 (Additional file 7: Table S2). The Cd-induced high expression of these genes may be one of the important components of Cd tolerance in Zhenong8. Heavy metals could alter membrane permeability properties and inhibit both proton transport and H^+^-ATPase activity, and the regulation of this enzyme may therefore be relevant to Cd tolerance [56]. The cation antiporter activity driven by the vacuolar ATPase-dependent proton motive force contributes significantly to the detoxification of Cd via vacuolar compartmentalization in plants [57]. In addition, Cd has a high affinity for metabolic processes related to sulfur metabolism via ATP- sulfurylase and adenosine 5′-phosphosulfate sulfotransferase [58]. Once Cd enters the cytosol, another system strictly related to sulfur metabolism is promptly activated to produce phytochelatins for Cd detoxification [56, 59].

### Suppression of *HvZIP* expression using antisense constructs confirms the role of *HvZIPs* in high accumulation of Cd in barley grains

Our microarray data provided strong evidence that up-regulation of genes encoding ZIP and many other membrane transporters was crucial for reducing Cd accumulation in W6nk2, both directly and indirectly (Fig. 4; Table 1). We investigated whether RNAi-mediated silencing of *HvZIPs* could lead to high Cd accumulation, constituting an opposite effect. Indeed, *HvZIP3* and *HvZIP8* antisense lines showed a large increase in Cd accumulation and a significant decrease in the Zn and Mn concentrations in barley grain (Fig. 6). ZIP transporters are involved in Zn, Fe, Mn and Cd homeostasis [26]. The rice ZIP transporter family has been shown to have diverse functions in Zn uptake in different tissues and cell types [60, 61]. Additionally, over-expression of Arabidopsis *AtZIP1* Zn transporter in barley results in increases in short-term Zn uptake and grain Zn contents [62]. The results with *HvZIP3* and *HvZIP8* RNAi lines suggested that up-regulation or over-expression of *HvZIP3* and *HvZIP8* genes may result in lower Cd transport and accumulation. Further efforts will be devoted to comprehensively characterizing each of these transporter genes and their potential roles in both low Cd accumulation and Cd tolerance.

## Conclusion

Together with comprehensive physiological analysis of the two contrasting barley genotypes, microarray analysis of the complex Cd-induced gene expression pattern and *ZIP* RNAi transgenic analysis provided new insights contributing to a more in-depth understanding of the low Cd accumulation and Cd tolerance observed in two contrasting barley genotypes. Cd stress resulted in large-scale changes in gene expression, which differed substantially between the two genotypes. Cd stress led to higher expression of genes related to transport, carbohydrate metabolism and signal transduction in the low-grain-Cd-accumulating genotype W6nk2, whereas it up-regulated genes involved in photosynthesis, protein synthesis, stress anddefense responses and transcription in the high-grain-Cd-accumulating, Cd-tolerant genotype Zhenong8. In addition, it was suggested that the Cd-induced high expression of genes encoding ZIP and ABC transporters is associated with low Cd accumulation in barley. RNAi analysis further validated the functions of *HvZIP3* and *HvZIP8* related to low grain Cd accumulation. The results illustrated that many of these transporters could be applied in biotechnology to decrease Cd accumulation in grains and improve Cd tolerance in barley. However, elucidating the mechanisms underlying Cd stress accumulation and tolerance in barley remains an ambitious endeavor for future research.

## Methods

### Plant materials and growth conditions

A hydroponic experiment using the barley genotypes Zhenong8 (high grain Cd accumulating) and W6nk2 (low grain Cd accumulating) identified by Chen et al., which was carried out on Huajiachi Campus, Zhejiang University, Hangzhou, China [20]. Healthy seeds were surface sterilized with 2 % H_2_O_2_ for 30 min and then rinsed with deionized water and soaked for 4 h. The seeds were germinated and grown hydroponically as described by Chen et al. [19]. The composition of basic nutrient solution (BNS) was the same as Chen et al. [19]. There were 4 treatments: control (BNS without Cd), 5 μM Cd, 50 μM Cd, and 500 μM Cd. The experiment was laid in a split-plot design with treatment as the main plot, and there were 6 replicates for each treatment. The solution pH was adjusted to 5.8 ± 0.1 with NaOH or HCl as required. The nutrient solution was continuously aerated with pumps and renewed every 5 days.

After 5, 10, and 15 days of Cd treatment, shoot and root samples were collected to determine metal concentrations according to Chen et al. [20]. Root tips were used for the imaging of Cd fluorescence. After 15 days of Cd treatment, the second fully expanded leaves were selected for chlorophyll content determination and photosynthetic measurements. These plants were harvested and separated into roots and shoots (stems and leaves), and plant height, root length, and the shoot and root dry weight were subsequently determined. Microarray analysis was performed using the leaf samples (fresh leaves were sampled and immediately frozen in liquid nitrogen) after 15 days of exposure to 0 (control) or 5 μM Cd.

Barley plants (*cv*. Golden Promise) for producing immature embryos for plant transformation were grown in climate chamber in pots with 50:50 peat and perlite mix under a cycle of 16 h light period (light intensity of 350 l E m-^2^ s^−1^) at 15 °C and 8 h darkness at 10 °C. After successful transformation, 6 proven transgenic primary regenerants were grown to maturity in a greenhouse under a cycle of 16 h illumination and 8 h of darkness at 23 °C and 18 °C, respectively till grain maturity and for further analysis.

The pot experiments were performed using T1 grains of the 6 transgenic lines and parental control (Golden Promise). Healthy T1 seeds were sowed in pot with 5 kg soil irrigated with nutrient solution 10 day before. Every pot was planted with 5 plants. There were two treatments for these experiments: control with no Cd addition and Cd treatment with artificial contamination of 5 mg kg^−1^ Cd in soil. The matured grains (T2) were harvested to use for elements analyses and grain ultrastructure examination.

### Measurement of chlorophyll contents, chlorophyll fluorescence and photosynthetic parameters

Chlorophyll content of the 2nd fully expanded leaves was determined according to Wellburn [63]. Chlorophyll fluorescence parameters including the initial fluorescence (Fo), light energy conversion efficiency of PSII (Fv/Fm), photosynthetic quantum yield of photosystem II (ΦPSII) and coefficient of photochemical quenching (qP) were measured using a pulse-modulated fluorometer and ImagingWin software (IMAGING-PAM; Walz, Effeltrich, Germany) according to Cai et al. [64]. Net photosynthetic rate (Pn), stomatal conductance (Gs), transpiration rate (Tr) and intercellular CO_2_ concentration (Ci) in the 2nd fully expanded leaves were monitored using an LI-6400 portable photosynthesis system (Li-COR, Lincoln, NE, USA).

### Confocal imaging of Cd in roots

Cd imaging of 1 cm fresh root tips were observed after immersed in Cd probe solution named Leadmium™ Green AM dye (Molecular Probes, Invitrogen, USA) according to Cao et al. method using a laser scanning confocal microscope (TCS SP5, Wetzlar, Germany) with excitation and emission wavelengths of 488 and 515 nm, respectively [3]. And the fluorescence density of Cd was calculated by selecting the root tips sections in the figures and measuring the total Integrated Density using “Analyse and Measure” function of the Image J software (NIH, Bethesda, MD, USA).

### Affymetrix GeneChip analysis

After 15 days of Cd exposure, the first fully expanded leaves were used for Affymetrix GeneChip analysis. RNA was isolated using TRIzol reagent protocol (Life Technologies, Carlsbad, CA, USA). Sample processing, cDNA synthesis, biotin-labeled cRNA synthesis, hybridization, washing, staining and scanning of the Affymetrix Barley 1.0 GeneChips were performed following the standard Affymetrix protocol. GeneChip data analysis was conducted using Affymetrix GeneChip Operating Software Version 1.4. Detection signal condensation and normalization were conducted *via* robust multichip analysis [65]. To detect barley transcripts, differences in the abundance of transcript signals in W6nk2 and Zhenong8 were tested against the background signal in CS *via t*-tests (*P*-value <0.001). Only those barley transcripts showing significantly higher levels in *cv*. W6nk2 and Zhenong8 compared to CS were subjected to quantitative analysis (presence/absence test with a *P*-value < 0.001, using MAS 5.0). All of the transcriptome data have been deposited at Gene Expression Omnibus (GSE73817). A two-fold change in gene expression was considered significant. To perform hierarchical clustering, the differentially regulated genes were clustered according to similarities in expression profiles. The following 4 categories were used: up-regulated in W6nk2 but down-regulated or unchanged in Zhenong8, or no change in W6nk2 but down-regulated in Zhenong8; up-regulated in Zhenong8 but down-regulated or unchanged in W6nk2, or unchanged in Zhenong8 but down-regulated in W6nk2; up-regulated in both genotypes; down-regulated in both genotypes.

### *qRT-PCR* of targeting genes

The hydroponic experiment was carried out again using W6nk2 and Zhenong8 under control and 5 μM Cd treatment with four replicates. Total RNA was isolated from leaves of barley plants after 15 days of Cd treatment using TRIzol reagent (Invitrogen, Karlsruhe, Germany). cDNA synthesis and qRT-PCR were carried out according to Cao et al. [3]. The PCR conditions consisted of denaturation at 95 °C for 3 min, followed by 40 cycles of denaturation at 94 °C for 1 min, annealing at 58 °C for 30 s and extension at 72 °C for 30 s, with a final extension at 72 °C for 5 min.

### Binary vector construction and barley transformation

Fragments (~317 bp) of *HvZIP* genes [Gene Bank Accession Numbers: FJ208991.1 (*ZIP3)* and FJ208993.1 (*ZIP8*)] were amplified from barley genomic DNA with the primers GTGCATTCAGTGATAATTGGCG (5′ to 3′) and AGGTCCACTAGGGACATGTAGA (3′ to 5′) for *HvZIP3* and CGCCAAGCTCATCCGTCACCGC (5′ to 3′) and TCCATTGTTCTGCACCCTAGGA (3′ to 5′) for *HvZIP8*. The fragments were then cloned into the XmnI and EcoRV digested entry vector pENTR4 (Invitrogen, Karlsruhe, Germany) by standard gene cloning methods. The constructs were transformed into MAX Efficiency® DH5α™ Competent Cells according the manufacturer instruction (Life Technologies, Ltd). DNA sequencing (Eurofins MWG GmbH, Germany) verified the positive clones. To create RNAi constructs, the inserts from the pENTR4 clones were introduced to the binary destination vector, pSTARGATE (the map of the pSTARGATE vector can be found at http://www.pi.csiro.au/RNAi/vectors.htm) using Gateway technology according to the manufacturer protocol (Invitrogen Inc.) and transformed into MAX Efficiency® DH5α™ Competent Cells. The resulting selected positive clones of both constructs were verified by sequencing (Eurofins MWG GmbH, Germany). The confirmed constructs was transformed into *Agrobacterium tumefaciens* strain AGL0 by the freeze-thaw method.

The *Agrobacterium tumefaciens* mediated transformation of immature barley zygotic embryos (approximately 14 days after pollination, with an embryo length of 1–2 mm) was carried out using hygromycin selection, as described by Mathews et al. [66]. The primers for screening barley RNAi transgenic lines were designed from ubiquitin promoter region: Forward 5′-CGA CGA GTC TAA CGG ACA CC-3′ and Reverse primer 5′- AAA CCA AAC CCT ATG CAA CG 3′.

### qRT-PCR of ZIP in the transgenic lines

Healthy grains of T0and T1 transgenic lines and Golden Promise parentwere surface sterilized with 2 % H_2_O_2_ for 30 min and then rinsed with deionized water and soaked for 4 h and germinated in sterilized moist vermiculite in a growth room at 22–25 °C. Seven days after germinating, fresh leaves were gathered and stored in −70 °C. The procedure of qRT-PCR was described as before. The primers of *HvZIP* were same with the amplified primers described above. And reference gene *actin* are shown as forward-5′-TGGCTGACGGTGAGGACA-3′, reverse-5′-CGAGGGCGACCAACTATG-3′.

### Analyses of grain metal concentrations and examination of ultrastructure of grains

The concentrations of Cd, Zn and Mn in the grains of the transgenic lines were analyzed according to Chen et al. [20]. The scanning electron microscopy (SEM) analysis of endosperm, central part of the transverse section was carried out according to Sun et al. [67].

### Statistical analysis

All data are the average of at least three independent replicates. Statistical analyses were performed with Data Processing System (DPS) (Beijing, China) statistical software package using ANOVA followed by Duncan’s Multiple Range Test (SSR) to evaluate the treatment effects at significance level of *P* ≤ 0.05.

### Availability of supporting data

The raw transcriptome data are available in the Gene Expression Omnibus (GEO) with the GEO accession number GSE73817 (http://www.ncbi.nlm.nih.gov/geo/query/acc.cgi?acc=GSE73817).

## One-sentence summary

Zn- and iron-regulated transporters (ZIP) genes, *HvZIP3* and *HvZIP8* could facilitate low Cd accumulation in barley grains.

## Additional files

Additional file 1: Figure S1. Plant height, root length, shoot and root dry weight of two barley genotypes exposure to Cd for 15 days. (PDF 86 kb) Additional file 2: Figure S2. The potosynthetic parameters and the chlorophyll fluorescence of two barley genotypes exposure to Cd for 15 days. (DOC 408 kb) Additional file 3: Figure S3. Chlorophyll content in leaves of two barley genotypes. (DOC 150 kb) Additional file 4: Figure S4. Leaf transcriptome profiles of Cd stress-responsive genes in barley. (DOC 132 kb) Additional file 5: Figure S5. Functional categorization differential expression of Cd-regulated genes in barley leaves. (DOC 181 kb) Additional file 6: Table S1. List of genes up-regulated in W6nk2 and down-regulated or non-change in Zhenong8 after 15 days exposure to 5 μM Cd except for transport related genes. (PDF 146 kb) Additional file 7: Table S2. List of genes up-regulated in Zhenong8 and down-regulated or no change in W6nk2, or no change in Zhenong8 and down-regulated in W6nk2 after 15 days exposure to 5 μM Cd. (PDF 152 kb) Additional file 8: Table S3. List of genes up-regulated in both W6nk2 and Zhenong8 after 15 days exposure to 5 μM Cd. (DOC 109 kb) Additional file 9: Table S4. List of genes down-regulated in both W6nk2 and Zhenong8 after 15 days exposure to 5 μM Cd. (DOC 130 kb) Additional file 10: Figure S6. Cd-induced differential genes expression except for transport related in leaves of two barley genotypes. (DOC 407 kb) Additional file 11: Figure S7. Integrated schematic of the mechanisms involved in Cd high accumulation and tolerance. (PDF 183 kb) Additional file 12: Table S5. The sequence of primers for RT-PCR. (DOC 73 kb) Additional file 13: Figure S8. Molecular cloning and RNAi analysis of *HvZIP3* and *HvZIP8*. (DOC 153 kb) Additional file 14: Figure S9. Sequence aligments of ZIP3 and ZIP8. (DOC 64 kb)
