# Supplementary material for: DNA microarray revealed and RNAi plants confirmed key genes conferring low Cd accumulation in barley grains
Source: BMC Plant Biol. 2015 Oct 26;15:259. doi: 10.1186/s12870-015-0648-5 (PMC4623906; doi:10.1186/s12870-015-0648-5)
Supplement: Additional file 1: Figure S1. — Plant height, root length, shoot and root dry weight of two barley genotypes exposure to Cd for 15 days. (PDF 86 kb) [file 12870_2015_648_MOESM1_ESM.pdf]

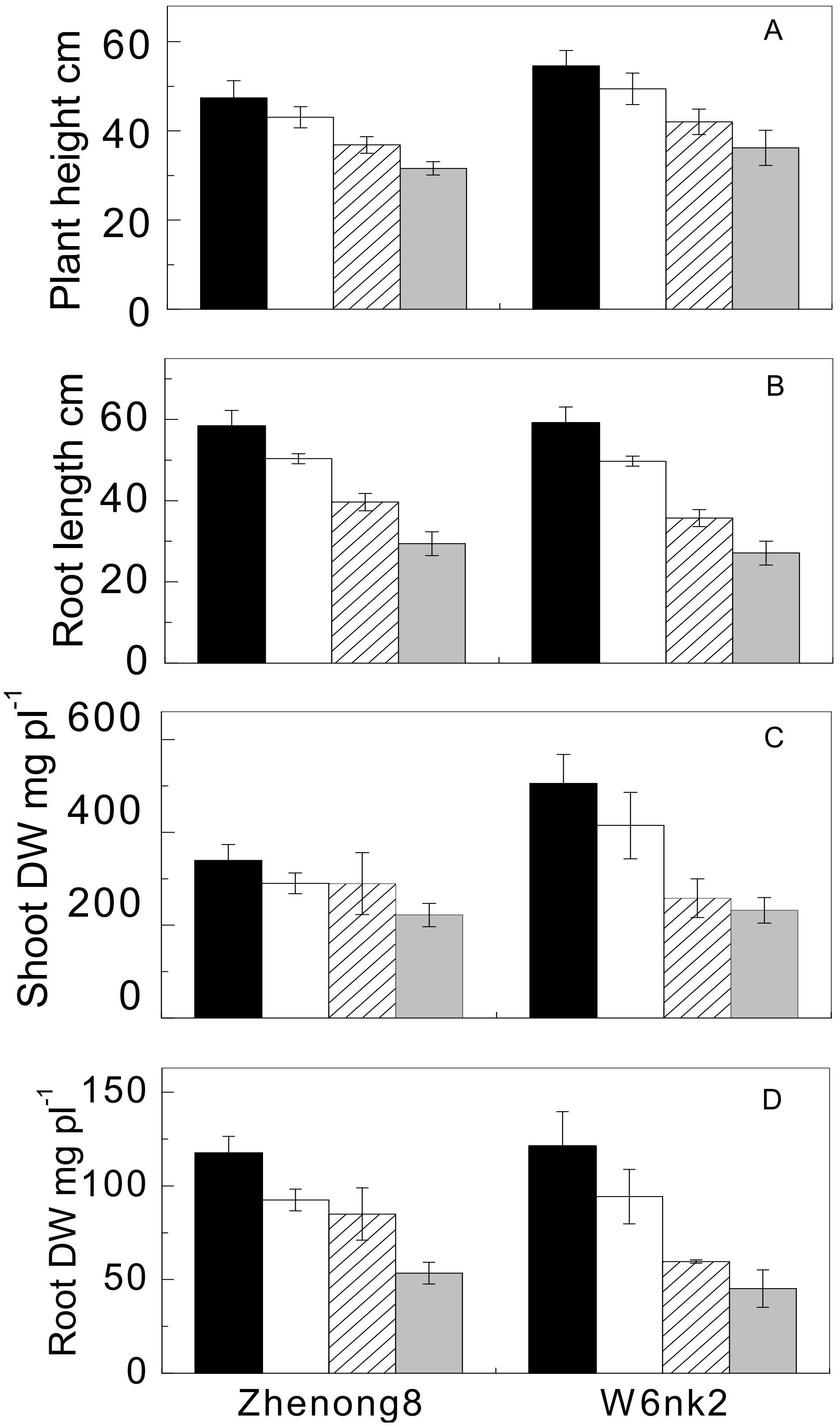

Fig. S1 Plant height, root length, shoot and root dry weight of two barley genotypes exposure to Cd for 15 days. Error bars represent SD values (n=3). (black, white, shaded and grey *bars* represent control, 5, 50, 500 μM Cd respectively, error bars represent SD values (n=3). DW represents dry weight.
