# Supplementary material for: DNA microarray revealed and RNAi plants confirmed key genes conferring low Cd accumulation in barley grains
Source: BMC Plant Biol. 2015 Oct 26;15:259. doi: 10.1186/s12870-015-0648-5 (PMC4623906; doi:10.1186/s12870-015-0648-5)
Supplement: Additional file 3: Figure S3. — Chlorophyll content in leaves of two barley genotypes. (DOC 150 kb) [file 12870_2015_648_MOESM3_ESM.doc]

**Additional file 3**

A

B

**Fig. S3** Chlorophyll content in leaves of two barley genotypes (Zhenong 8 and W6nk 2) exposed to Cd for 15 days. Error bars represent SD values (n=3). (black, white, shaded and grey *bars* represent control, 5, 50, 500 µM Cd respectively, error bars represent SD values (n=3). DW represents dry weight.
