# Supplementary material for: DNA microarray revealed and RNAi plants confirmed key genes conferring low Cd accumulation in barley grains
Source: BMC Plant Biol. 2015 Oct 26;15:259. doi: 10.1186/s12870-015-0648-5 (PMC4623906; doi:10.1186/s12870-015-0648-5)
Supplement: Additional file 4: Figure S4. — Leaf transcriptome profiles of Cd stress-responsive genes in barley. (DOC 132 kb) [file 12870_2015_648_MOESM4_ESM.doc]

**Additional file 4**

W-*up*

Z-*up*

272

31

Z-*down*

93

13

Z-*NC*

65

338

A. up-regulated in W6nk2

W-*NC*

Z-*NC*

403

Z-*up*

54

249

Z-*down*

56

50

B. no change in W6nk2

B

C

W-*down*

Z-*down*

63

43

Z-*NC*

338

65

Z-*up*

280

23

C. down-regulated in W6nk2

B

C

**Fig. S4** Leaf transcriptome profiles of Cd stress-responsive genes in barley. Venn diagrams show the number of genes regulated by Cd treatment (5 µM Cd stress for 15 days) and overlap between Zhenong 8 (Z) and W6nk2 (W). The data are genes, which were up-regulated (up) (A), no change (NC) (B) and down-regulated (down) (C) in W6nk2, while in Zhenong8 which are down-regulated or up-regulated or no change. Within each genotype, transcript abundances of the genes showing a Cd treatment to controls ratio greater than ±2 (P<0.05) are used in the analysis.
