# Supplementary material for: DNA microarray revealed and RNAi plants confirmed key genes conferring low Cd accumulation in barley grains
Source: BMC Plant Biol. 2015 Oct 26;15:259. doi: 10.1186/s12870-015-0648-5 (PMC4623906; doi:10.1186/s12870-015-0648-5)
Supplement: Additional file 5: Figure S5. — Functional categorization differential expression of Cd-regulated genes in barley leaves. (DOC 181 kb) [file 12870_2015_648_MOESM5_ESM.doc]

**Additional file 5**

C. up-regulated in both D. down-regulated in both


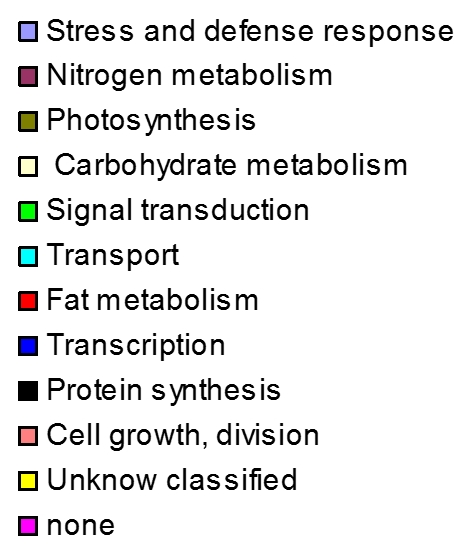


A. higher expression in

W6nk2 than Zhenong8

B. lower expression in

W6nk2 than Zhenong8

**Fig. S5** Functional categorization differential expression of Cd-regulated genes in barley leaves**.** Functional categorization was performed using the agriGO methods. Pie charts show the distribution of different functional transcripts after exposing the plants to 5 µM Cd for 15 days. (A) category1, up-regulated in W6nk2 and down-regulated or no change in Zheong8, or no change in W6nk2 and down-regulated in Zhenong8; (B) category2, up-regulated in Zhenong8 and down-regulated in W6nk2, or no change in Zheong8 and down-regulated in W6nk2; (C) category 3, up-regulated in both genotypes, (D) category 4, down-regulated in both genotypes.
