# Supplementary material for: DNA microarray revealed and RNAi plants confirmed key genes conferring low Cd accumulation in barley grains
Source: BMC Plant Biol. 2015 Oct 26;15:259. doi: 10.1186/s12870-015-0648-5 (PMC4623906; doi:10.1186/s12870-015-0648-5)
Supplement: Additional file 6: Table S1. — List of genes up-regulated in W6nk2 and down-regulated or non-change in Zhenong8 after 15 days exposure to 5 μM Cd except for transport related genes. (PDF 146 kb) [file 12870_2015_648_MOESM6_ESM.pdf]

Table S1 List of genes except for transport related whose expression were up-regulated in W6nk2 and down-regulated or no change in Zhenong8, and no change in W6nk2 and down regulated in Zhenong8 after 15 days exposure to 5 μM Cd.

Annotation	Probe ID	Fold change* (Cd treatment *vs* control)		Accession No	E-value
		W6nk2	Zhenong8		
**Stress and defense response**					
Wheat aluminum induced protein wali 5 [*Triticum aestivum*]	Contig2243_s_at	3.84	-2.30	JQ2361	8e-40
Wheat aluminum induced protein wali 3 [*T. aestivum*]	HVSMEb0009H14r2_s_at	2.89	-2.10	JQ2360	4e-40
Pathogen-related protein [*Hordeum vulgare*]	Contig5607_s_at	3.36	-2.06	P16273	e-128
Proteinase inhibitor-related protein [*H. vulgare*]	HD07M22r_s_at	3.42	-4.53	S53102	2e-29
Probenazole-inducible protein PBZ1 [*Oryza. sativa* (japonica)]	HD08F14r_x_at	7.62	-1.95	T02973	e-19
Pathogenesis-related protein [*H. vulgare*]	Contig4056_s_at	13.42	-1.90	P32937	7e-46
Peroxidase [*O. a sativa*]	Contig2115_at	2.31	-1.82	AAC49819.1	e-79
T06168 pathogenesis related protein [*H. vulgare*]	Contig5605_at	2.09	-1.82	P16273	e-88
Pathogenesis-related protein PR-10a [*O. sativa*]	Contig4405_x_at	3.36	-1.77	AAF85972.1	6e-36
Pathogenesis-related protein 1a precursor [*H. vulgare*]	Contig4056_at	14.80	-1.76	P32937	7e-46
Wheat aluminum induced protein wali 3 [*T. aestivum*]	Contig4750_at	2.44	-1.76	JQ2360	e-43
Pathogen-induced protein WIR1A [*T. aestivum*]	Contig6519_at	7.44	-1.74	Q01482	8e-12
Pathogenesis-related protein 1c precursor [*H. vulgare*]	Contig4054_s_at	12.93	-1.69	P32938	3e-84
Similar to human dimethylaniline monooxygenase [*O. sativa* (japonica)]	Contig11792_at	4.27	-1.68	BAA88198.1	4e-30
Class III chitinase [*O. sativa subsp.* japonica]	Contig5023_at	4.39	-1.65	AAG02504.1	2e-96
Pathogenesis-related protein PR-10a [*O. sativa*]	Contig4402_s_at	10.46	-1.55	AAF85972.1	6e-29
Putative stripe rust resistance protein Yr10 [*Sorghum bicolor*]	Contig18459_at	3.73	-1.55	AAM94294.1	4e-09
Permatin homolog PR5 [*H. vulgare subsp.*]	Contig2787_s_at	22.69	-1.52	T05973	e-133
Peroxidase (EC 1.11.1.7) [*H. vulgare*]	HVSMEm0005P05r2_at	18.67	-1.51	S14611	2e-23
Subtilisin-chymotrypsin inhibitor 2 [*H. vulgare subsp.*]	Contig3380_s_at	4.82	-1.50	T06181	9e-19
Wheat aluminum induced protein wali 3 [*T.aestivum*]	Contig4751_at	3.10	-1.49	JQ2360	6e-07
Putative stripe rust resistance protein Yr10 [*S. bicolor*]	baak13l10_s_at	3.61	-1.41	AAM94294.1	6e-05
Pathogenesis-related protein PR-10a [*O. sativa*]	Contig4406_x_at	2.67	-1.41	AAF85972.1	2e-38
Putative cytochrome P450 [*O. sativa* (japonica)]	Contig17284_at	3.43	-1.37	BAB19121.1	4e-62
Heat shock protein 101 [*T. aestivum*]	Contig21775_at	2.39	-1.26	AAF01280.1	2e-35
Pathogenesis-related protein type [*Sambucus nigra*]	Contig14498_at	2.47	-1.25	S51678	2e-57
OSJNBb0086G13.5 [*O. sativa* (japonica)]	Contig4131_at	2.10	-1.24	CAD41021.1	2e-95
Glutamate dehydrogenase [*Brassica napus*]	Contig4928_at	2.19	-1.23	BAB62170.1	e-105
Pathogenesis related protein-1 [*Zea mays*]	Contig12046_at	29.26	-1.20	T02054	5e-57
Chitinase IV precursor [*T. aestivum*]	Contig4326_s_at	3.97	-1.19	AAD28733.1	4e-69
Putative wall-associated kinase 1 [*O. sativa*]	HT06F11u_s_at	2.92	-1.19	P55308	e-32

Annotation	Probe ID	Fold change* (Cd treatment vs control)		Accession No	E-value
		W6nk2	Zhenong8		
Pathogenesis-related protein 4 [*H. vulgare*]	Contig2550_x_at	14.31	-1.18	T06169	8e-77
Chitinase [*H. vulgare subsp. vulgare*]	Contig2992_s_at	3.43	-1.17	S48848	e-133
Pathogen-induced protein WIR1A [*T. aestivum*]	Contig23878_x_at	9.00	-1.16	Q01482	0.001
Heat shock protein 90 homolog precursor [*H. vulgare*]	Contig91_at	2.64	-1.16	P36183	e-121
Pathogen-induced protein WIR1A [*T. aestivum*]	Contig813_at	2.62	-1.16	Q01482	3e-07
Peroxidase precursor, pathogen-induced [*H. vulgare*]	Contig2118_at	3.55	-1.15	T06172	e-154
Chitinase IV precursor [*T. aestivum*]	Contig4326_at	5.17	-1.13	AAD28733.1	4e-69
Putative stripe rust resistance protein [*O. sativa* (japonica)]	Contig17047_at	3.67	-1.12	BAB64601.1	4e-44
pathogenesis-related protein 4 [*H. vulgare*]	Contig639_at	20.33	-1.08	T06171	4e-12
CI2E [*H. vulgare*]	HP0e21w_s_at	2.61	-1.06	AAM22827.1	2e-14
Pathogenisis-related protein 1.2 [*T. aestivum*]	Contig2208_at	4.27	-1.01	CAA07474.1	9e-88
Pathogenesis-related protein 1 precursor [*H. vulgare*]	Contig2210_at	16.29	1.00	Q05968	2e-80
Pathogenesis related protein [*H. vulgare subsp.*]	Contig2214_s_at	20.40	1.02	S37166	5e-81
Pathogenesis-related protein PRB1-2 precursor [*H. vulgare*]	Contig2211_at	6.92	1.03	P35792	e-80
Pathogen-induced protein WIR1A [*T. aestivum*]	Contig5974_s_at	11.32	1.04	Q01482	e-24
Peroxidase [*T. aestivum*]	rbah13p07_s_at	8.32	1.04	AAM76682.1	2e-24
Pathogenesis-related protein 1a [*H. vulgare subsp.*]	Contig2209_at	14.26	1.06	S37166	5e-81
Defensin [*T. aestivum*]	Contig3216_at	2.73	1.07	BAC10287.1	e-20
Putative peroxidase [*O. sativa* (japonica)]	Contig11361_at	5.07	1.08	AAL34125.1	2e-73
Pathogenesis-related protein PRB1-3 precursor [*H. vulgare*]	Contig2212_s_at	35.35	1.09	P35793	8e-88
Chitinase [*H. vulgare subsp. vulgare*]	Contig2990_at	7.63	1.09	S48847	e-134
Putative cytochrome P450 [*O. sativa* (japonica)]	Contig18990_at	2.41	1.09	BAB39252.1	2e-40
Peroxidase [*T. aestivum*]	Contig2112_at	3.54	1.17	S61406	e-103
Disease resistance response protein-related [*Arabidopsis thaliana*]	Contig10245_at	5.62	1.20	NP_176113.1	9e-40
Type-1 pathogenesis-related protein [*H. vulgare*]	Contig2213_s_at	6.31	1.22	S53101	e-100
DnaJ protein homolog - kidney bean [*Phaseolus vulgaris*]	Contig7255_at	2.21	1.25	T11855	3e-19
Globulin-like protein [*A. thaliana*]	Contig10263_at	2.16	1.26	NP_172255.1	8e-11
Chitinase [*O. sativa* (japonica)]	Contig23540_at	3.35	1.27	JC5846	e-40
Chitinase II precursor [*T. aestivum*]	Contig4324_s_at	6.71	1.31	AAD28730.1	5e-73
Argininosuccinate lyase (AtArgH) [*A. thaliana*]	Contig6113_at	3.00	1.32	NP_196653.1	4e-97
Barwin homolog wheatwin2 precursor [*T. aestivum*]	Contig2546_at	2.65	1.32	T06486	3e-64
Chitinase II precursor [*T. aestivum*]	Contig4324_at	9.07	1.36	AAD28730.1	5e-73
Hypersensitive-induced reaction protein 3 [*H. vulgare subsp.*]	Contig3626_s_at	4.62	1.36	AAN17464.1	e-126
Putative lipoxygenase [*O. sativa* (japonica)]	Contig12574_at	2.55	1.36	AAL83618.1	e-43

Annotation	Probe ID	Fold change* (Cd treatment vs control)		Accession No	E-value
		W6nk2	Zhenong8		
Putative peroxidase [*O. sativa* (japonica)]	Contig19929_at	3.20	1.41	BAB89258.1	e-17
Pathogen-related protein [*O. sativa*]	Contig5368_at	2.80	1.44	AAL27005.1	e-68
Stem rust resistance protein Rpg1 [*H. vulgare subsp. vulgare*]	Contig6547_at	2.54	1.46	AAM76922.1	4e-50
Senescence-associated protein 5 [*Hemerocallis hybrid*]	Contig3054_s_at	2.42	1.48	AAC34855.1	4e-66
Allene oxide synthase [*H. vulgare subsp. vulgare*]	Contig3096_s_at	3.61	1.52	CAB86384.1	e-121
Putative hypersensitivity-related protein [*O. sativa* (japonica)]	Contig19684_at	3.48	1.53	AAG13627.1	5e-35
Germin-like 12 [*H. vulgare*]	Contig3155_s_at	2.21	1.53	T05956	5e-98
Ribosomal protein L17.1, cytosolic [*H. vulgare*]	rbags1i23_s_at	2.00	1.53	S32578	2e-43
Putative protein, F-box protein PP2-A13 [*A. thaliana*]	Contig11328_at	2.28	1.60	NP_567108.1	5e-15
Germin A [*H. vulgare*]	Contig3151_at	3.68	1.62	AAG00425.1	e-118
Xylanase inhibitor protein I [*T. aestivum*]	Contig8905_at	2.10	1.62	CAD19479.1	e-99
Ribosomal protein S15 [*A. thaliana*]	Contig2523_at	2.00	1.62	NP_172256.1	6e-69
Germin E [*H. vulgare*]	AF250937_s_at	4.18	1.67	AAG00429.1	e-104
Oxalate oxidase-like protein or germin-like protein [*H. vulgare*]	Contig3157_at	3.17	1.69	T05956	e-45
Oxalate oxidase [*T. aestivum*]	Contig1518_at	5.16	1.73	AAF34811.1	e-117
Pathogen-related protein [*O. sativa*]	Contig5369_at	4.68	1.73	AAL27005.1	9e-79
Putative iron/ascorbate-dependent oxidoreductase [*O. sativa* (japonica)]	Contig4273_at	7.06	1.13	BAA95828.1	6e-52
putative iron/ascorbate-dependent oxidoreductase [*O. sativa* (japonica)]	Contig3568_at	2.41	-1.41	BAA95828.1	7e-86
Putative iron/ascorbate-dependent oxidoreductase [*O.sativa* (japonica)]	Contig3563_at	2.42	-1.61	BAA95828.1	2e-47
Similar to Lycopersicon pimpinellifolium Cf-2 gene [*O. sativa* (japonica)]	Contig23814_at	2.16	-1.18	BAA99381.1	4e-50
Thionin [*H. vulgare*]	Contig1579_s_at	3.26	1.60	AAB21531.1	2e-67
Thaumatin-like protein TLP8 [*H. vulgare*]	EBem10_SQ002_I10_s_at	12.51	-1.37	AAK55326.1	8e-04
Thaumatin-like protein TLP7 [*H. vulgare*]	Contig2789_at	24.03	-1.35	AAK55325.1	e-117
WIR1 protein [*T. aestivum*]	Contig9917_at	8.15	-1.33	S55368	e-04
Thaumatin-like protein TLP7 [*H. vulgare*]	Contig2790_s_at	10.94	-1.31	AAK55325.1	5e-74
Thaumatin-like protein TLP4 [*H. vulgare*]	Contig3947_s_at	2.26	-1.29	AAK55323.1	5e-71
Osmotin-like protein [*O. sativa* (japonica)]	Contig9094_at	2.50	-1.28	BAB67891.1	2e-63
GRAB2 protein [*T. aestivum sp.*]	Contig9031_at	3.56	1.58	CAA09372.1	5e-75
Harpin induced gene 1 homolog [*O. sativa*]	Contig3744_s_at	2.56	1.23	T02662	3e-60
Harpin induced gene 1 homolog [*O. sativa*]	Contig3746_at	2.40	1.24	T02662	e-72
T06988 pathogen-induced protein WIR1A [*T. aestivum*]	Contig939_s_at	2.18	1.07	Q01482	8e-10
T06988 pathogen-induced protein WIR1A [*T. aestivum*]	Contig2163_at	7.43	1.08	Q01482	e-07
T06988 pathogen-induced protein WIR1A [*T.*	Contig2170_at	2.61	1.09	Q01482	3e-08

Annotation	Probe ID	Fold change* (Cd treatment vs control)		Accession No	E-value
		W6nk2	Zhenong8		
aestivum]					
Hemolysin [*Acanthamoeba polyphaga*]	HS08O16u_s_at	-1.6	-2.11	AAA58585.2	2e-07
Subtilisin-chymotrypsin inhibitor 2 [*H. vulgare subsp.*]	Contig3381_s_at	1.75	-2.32	T06181	5e-33
Physical impedance induced protein [*Z. mays*]	Contig3783_at	1.2	-3.14	AAC31615.1	2e-17
Physical impedance induced protein [*Z. mays*]	Contig3783_s_at	1.33	-2.84	AAC31615.1	2e-17
Fatty acid alpha-oxidase [*O. sativa*]	Contig15882_s_at	1.39	-2.78	AAF64042.1	6e-79
Putative heat shock protein [*O. sativa*]	Contig17190_at	-1.86	-2.47	AAL83988.1	2e-36
Phenylalanine ammonia-lyase [*H. vulgare*]	HVSMEm0015M15r2_s_at	-1.15	-4.1	T05968	3e-13
Putative peroxidase [*O. sativa* (japonica)]	EBro03_SQ003_J21_at	1.18	-2.13	BAB63623.1	2e-19
**Carbohydrate metabolism**					
Putative indole-3-glycerol phosphate synthase [*A. thaliana*]	Contig6407_s_at	6.05	-4.26	AAM64536.1	3e-75
Putative indole-3-glycerol phosphate synthase [*A. thaliana*]	Contig6407_at	4.08	-3.05	AAM64536.1	3e-75
Putative cyanase [*O. sativa*]	Contig13114_at	2.74	-1.65	AAG21913.1	7e-08
Endo-1,3-beta-glucanase [*O. sativa*]	Contig11289_at	23.97	-1.46	AAL35900.1	2e-59
Ceta-1,3-glucanase precursor [*T. aestivum*]	Contig13350_at	14.33	-1.45	AAD28734.1	7e-65
Cinnamyl alcohol dehydrogenase 1a [*Festuca arundinacea*]	Contig4346_at	2.65	-1.24	AAK97808.1	e-90
Glucan endo-1,3-beta-D-glucosidase [*H. vulgare*]	Contig1637_s_at	32.33	-1.23	D38664	e-162
Glycosyl hydrolase family 17 [*A. thaliana*]	Contig18116_at	2.90	-1.15	NP_181494.1	e-30
Glucan endo-1,3-beta-D-glucosidase [*H. vulgare*]	Contig1637_at	31.65	-1.14	D38664	e-162
Palmitoyl-protein thioesterase precursor [*A. thaliana*]	Contig26597_at	2.47	-1.11	NP_191593.1	4e-66
Glucan endo-1,3-beta-D-glucosidase [*H. vulgare var.* distichum]	HVSMEm0003C15r2_x_at	2.14	-1.08	A31800	2e-48
Putative glucosyltransferase [*O. sativa* (japonica)]	Contig14830_at	2.93	-1.02	AAM01107.1	3e-87
Glucan endo-1,3-beta-glucosidase GIII [*H. vulgare*]	Contig1636_at	4.19	1.01	Q02126	e-164
Putative phospholipase [*A. thaliana*]	Contig19569_at	2.10	1.03	AAL87258.1	3e-49
Beta-1,3 glucanase-like protein [*O. sativa* (japonica)]	Contig13846_s_at	3.09	1.04	BAB90413.1	2e-24
Cinnamoyl-CoA reductase [*Z. mays*]	Contig8527_at	2.56	1.08	CAA75352.1	2e-92
Ubiquitin-specific protease 5 (UBP5) [*A. thaliana*]	Contig6229_s_at	2.89	1.13	NP_565944.1	2e-76
Beta-1,3 glucanase-like protein [*O. sativa* (japonica)]	Contig13846_at	4.41	1.18	BAB90413.1	2e-24
Glucan endo-1,3-beta-D-glucosidase [*H. vulgare var.* distichum]	HVSMEm0003C15r2_s_a	37.55	1.21	A31800	2e-48
Alcohol dehydrogenase ADH [*Lycopersicon esculentum*]	Contig13799_at	2.61	1.21	AAB33480.2	3e-45
Tyrosine phosphatase 1 [*G. max*]	Contig12732_at	2.10	1.21	CAA06975.1	5e-38
Putative flavanone 3-hydroxylase [*O. sativa* (japonica)]	Contig12724_at	2.18	1.25	AAL58118.1	e-100

Annotation	Probe ID	Fold change* (Cd treatment vs control)		Accession No	E-value
		W6nk2	Zhenong8		
Soluble inorganic pyrophosphatase [*Populus tremula x Populus tremuloides*]	Contig2021_at	2.58	1.28	AAD46520.1	2e-99
Cytosolic aldehyde dehydrogenase RF2C [*Z. mays*]	Contig6381_at	3.55	1.29	AAL99608.1	4e-97
Probable cinnamyl alcohol dehydrogenase 9 [*A. thaliana*]	Contig20411_at	2.45	1.34	NP_195643.1	9e-38
Putative xylanase inhibitor protein [*O. sativa* (japonica)]	Contig5996_s_at	2.10	1.34	BAC10141.1	e-30
Putative phospholipase [*O. sativa*]	Contig4805_at	2.35	1.35	AAK82449.1	e-120
Sec14 like protein [*O. sativa* (japonica)]	Contig10529_at	2.79	1.38	BAB89672.1	4e-85
Cytosolic aldehyde dehydrogenase RF2C [*Z. mays*]	Contig6382_s_at	2.16	1.40	AAL99608.1	e-106
Glycosyl hydrolase family 17 [*A. thaliana*]	Contig15553_at	2.14	1.54	NP_201128.1	5e-31
Alpha/beta hydrolase [*A. thaliana*]	Contig21945_at	2.68	1.56	NP_189622.1	2e-06
Putative protein phosphatase 2C [*O. sativa* (japonica)]	Contig13376_at	2.26	1.63	AAM08826.1	5e-66
Putative beta-glucosidase [*O. sativa* (japonica)]	rbaal5f06_at	2.07	1.72	BAB90397.1	3e-47
Apoplastic invertase [*O. sativa subsp.* indica]	Contig4470_s_at	2.90	1.88	AAD38399.1	e-94
Putative glucan 1,3-beta-glucosidase [*O. sativa* (japonica)]	Contig9266_at	7.46	1.89	AAM08620.1	3e-43
Putative PrMC3 [*O. sativa subsp.* japonica]	Contig20431_at	2.56	-1.16	BAB44059.1	2e-39
Putative uncharacterized protein At5g13260 [*A. thaliana*]	Contig15613_at	2.43	-1.19	NP_196830.2	3e-62
Delta-type tonoplast intrinsic protein [*T. aestivum*]	Contig1315_s_at	-1.53	-2.27	AAD10495.1	e-52
Putative HGA6 [*O. sativa*]	Contig4690_at	-1.84	-2.58	BAB17150.1	5e-68
Putative phosphoglycerate dehydrogenase [*O. sativa* (indica)]	Contig5494_at	-1.31	-3.27	CAC09348.1	3e-91
Laccase [*Pinus taeda*]	HV_CEb0017C08r2_at	1.17	-11.75	AAK37826.1	e-19
Formate dehydrogenase, mitochondrial precursor [*H. vulgare*]	HVSMEa0019P15r2_at	1.7	-2.28	Q9ZRI8	e-41
Cinnamyl alcohol dehydrogenase [*Eucalyptus saligna*]	HVSMEh0081I20r2_s_at	-1.07	-2.36	AAG15553.1	3e-10
**Transcription**					
Alanyl-tRNA synthetase (alaS) [*Sulfolobus solfataricus*]	HV_CEa0008J10r2_s_at	3.51	-1.34	NP_341881.1	0.12
Putative glycerophosphoryl diester phosphodiesterase [*O. sativa*]	Contig9141_at	2.13	-1.24	BAB92381.1	2e-66
Histone H2B.2 [*T. aestivum*]	Contig1179_at	2.54	-1.23	P05621	5e-44
Myb-related protein - barley [*H. vulgare subsp.* vulgare]	Contig3667_s_at	12.45	-1.13	T06179	e-164
F22O13.10 [*A. thaliana*]	Contig22204_at	2.08	-1.12	AAF99757.1	2e-15
Quinone-oxidoreductase QR2 [*Triphysaria versicolor*]	Contig5217_at	2.31	-1.11	AAG53945.1	2e-67
Putative WRKY DNA binding protein [*O. sativa* (japonica)]	Contig7517_at	2.00	-1.02	AAD38283.1	9e-26
RGA-like [*A. thaliana*]	Contig14853_at	2.18	-1.01	CAA12242.1	4e-15
Barwin homolog wheatwin2 precursor [*T.*	HT07J20u_x_at	6.90	1.02	T06486	2e-06

Annotation	Probe ID	Fold change* (Cd treatment *vs* control)		Accession No	E-value
		W6nk2	Zhenong8		
aestivum]					
DNA-binding protein 4 [*Nicotiana tabacum*]	Contig20450_at	3.18	1.02	T50861	e-15
Endonuclease [*H. vulgare subsp.* vulgare]	Contig4113_at	3.56	1.03	T04401	e-158
DNA-binding protein 3 [*N. tabacum*]	Contig15957_at	4.38	1.06	AAF61863.1	3e-12
APETALA2 protein homolog HAP2 [*Hyacinthus orientalis*]	Contig8369_at	2.10	1.07	AAD22495.3	4e-20
Putative chloroplast nucleoid DNA-binding protein [*O. sativa*]	Contig13091_s_at	2.55	1.08	AAL79734.1	3e-15
Similar to chloroplast nucleoid DNA binding protein [*O. sativa* (japonica)]	Contig25725_at	2.21	1.08	BAC15912.1	2e-29
Putative steroid membrane binding protein [*O. sativa* (japonica)]	Contig10724_at	2.48	1.09	AAG13623.1	e-58
DNA-binding protein RAV2-like [*O. sativa* (japonica)]	Contig7481_at	2.06	1.12	BAB84620.1	9e-24
Putative GDP dissociation inhibitor [*O. sativa* (japonica)]	HT01N03w_at	2.41	1.23	BAC10071.1	8e-48
Protein H2B153 [*T. aestivum*]	Contig1127_at	-1.94	-2.09	S56687	e-54
Gene prediction of OSJNBa0042L16.13 [*O.sativa*]	Contig7464_at	1.12	-3.21	CAD41015.1	4e-57
rRNA promoter binding protein [*Rattus norvegicus*]	HV11C08u_x_at	-1.85	-2.22	NP_671477.1	7e-08
**Nitrogen metabolism**					
Glutamine-dependent asparagine synthetase [*H. vulgare*]	HV11O04r_at	7.03	-4.66	AAK49456.1	5e-53
Putative tryptophan synthase alpha [*Z. mays*]	Contig5542_at	2.96	-3.34	AAG42689.1	e-65
Aromatic-L-amino-acid decarboxylase [*Catharanthus roseus*]	Contig11623_at	2.44	-1.46	P17770	2e-69
N-methyltransferase [*Coffea canephora*]	Contig26053_at	3.19	-1.41	AAM18506.1	3e-07
Putative glutamate carboxylase [*O. sativa* (japonica)]	Contig1385_at	2.22	-1.06	AAM47304.1	e-120
Bowman-birk type trypsin inhibitor (WTI) [*T.aestivum*]	Contig17082_at	8.89	1.20	P81713	5e-20
Putative thiolase [*O. sativa* (japonica)]	Contig5922_at	2.97	1.31	AAK54299.1	2e-99
Putative copper amine oxidase[*A. thaliana*]	Contig6001_at	2.05	1.31	NP_181777.1	e-132
Asparaginase [*H. vulgare subsp. vulgare*]	Contig8740_at	-1.24	-5.89	AAG28786.1	2e-71
Putative proline-rich protein [*O. sativa*]	Contig704_at	-1.92	-3.04	AAK63900.1	7e-40
**Fat metabolism**					
Putative glycerophosphodiester phosphodiesterase [*A. thaliana*]	HVSMEm0001J08r2_s_at	2.45	-1.08	NP_177561.1	8e-23
Allene oxide synthase [*A. thaliana*]	Contig11904_at	2.22	1.04	CAA63266.1	5e-48
F23N19.16 [*A. thaliana*]	Contig3699_s_at	2.65	1.09	AAF19539.1	4e-11
Probable lipoxygenase [*H. vulgare*]	Contig1737_at	2.24	1.13	T05943	e-141
GDSL-motif lipase/hydrolase-like protein [*A. thaliana*]	Contig15_s_at	2.69	1.33	NP_200316.1	3e-15
Putative phospholipase [*O. sativa*]	Contig7525_at	-1.44	-2.43	AAK50122.1	4e-50
Patatin-like protein [*A. thaliana*]	Contig20326_at	1.31	-2.3	NP_195422.1	4e-07

Annotation	Probe ID	Fold change* (Cd treatment vs control)		Accession No	E-value
		W6nk2	Zhenong8		
**Signal transduction**					
Secretory protein [*T. aestivum*]	Contig358_at	4.10	-1.94	AAD46133.1	e-100
Spot 3 protein and vacuolar sorting receptor homolog [*A. thaliana*]	Contig16182_at	2.27	-1.23	NP_174375.1	e-26
Putative seven transmembrane protein [*O. sativa* (japonica)]	Contig13968_at	4.92	-1.11	BAB92639.1	2e-56
Putative steroid membrane binding protein [*O. sativa* (japonica)]	HVSMEg0015I15r2_at	2.08	-1.11	AAG13629.1	3e-11
Secretory carrier membrane protein [*A. thaliana*]	Contig12788_at	2.71	-1.03	NP_174485.1	3e-78
Putative protein kinase [*O. sativa*]	Contig14572_at	3.88	-1.60	AAK02024.2	7e-86
Serine/threonine-protein kinase TAK14 [*T.aestivum*]	Contig4999_at	2.38	-1.40	AAK20744.1	4e-61
Putative protein kinase; protein id: At3g21630.1 [*A. thaliana*]	Contig9408_at	5.32	-1.36	NP_566689.1	5e-45
Wall-associated kinase 4. [*O. sativa* (japonica)]	Contig16619_at	7.91	-1.33	BAA95893.1	6e-62
Serine/ threonine and tyrosine protein kinases SERK2 protein [*Z. mays*]	Contig3635_s_at	14.51	-1.32	CAC37639.1	3e-59
SERK2 protein [*Z. mays*]	Contig3636_at	17.28	-1.30	CAC37639.1	8e-60
Similar to A. thaliana wak4 gene [*O. sativa* (japonica)]	Contig12770_at	2.98	-1.26	BAA95893.1	7e-88
Elicitor-responsive gene 3 [*O. sativa*]	Contig5942_at	2.18	-1.15	T50649	9e-64
Mitogen-activated protein kinase 1 [*A. sativa*]	Contig5531_at	2.02	-1.15	S56638	e-127
Putative receptor serine/threonine kinase [*O. sativa* (japonica)]	HG01J06u_at	2.76	-1.09	BAB64138.1	e-36
Serine/threonine kinase-like protein [*O. sativa* (japonica)]	Contig25448_at	2.00	-1.08	BAC20673.1	3e-38
Putative wall-associated kinase 1 [*O. sativa*]	Contig11886_at	4.11	-1.06	AAL76192.1	2e-60
Root phototropism protein 2 RPT2 [*A. thaliana*]	Contig24168_at	2.24	-1.03	AAF33112.1	5e-09
S-receptor kinase (EC 2.7.1.-) KIK1 precursor [*Z. mays*]	Contig13217_at	2.07	-1.03	T02053	2e-85
Putative protein kinase [*A. thaliana*]	Contig15719_at	2.13	-1.01	NP_180081.1	5e-31
Putative receptor-type protein kinase LRK1 [*O. sativa* (japonica)]	Contig16179_s_at	2.10	1.00	BAC06926.1	6e-44
Elicitor-responsive gene 3 [imported] [*O.sativa*]	Contig5943_s_at	2.15	1.03	T50649	e-63
Putative receptor-like protein kinase [*O. sativa* (japonica)]	Contig7061_s_at	2.09	1.03	AAN16323.1	2e-19
Receptor-like kinase ARK1AS [*T. aestivum*]	Contig4997_s_at	2.36	1.05	AAD43962.1	4e-90
Putative protein kinase Xa21, receptor type precursor [*O. sativa* (japonica)]	Contig4666_at	2.75	1.09	BAC10827.1	4e-78
Cysteine-rich repeat secretory protein 55 [*A. thaliana*]	Contig8557_at	2.04	1.20	NP_199665.1	e-35
Putative serine/threonine protein kinase [*O. sativa* (japonica)]	Contig15156_at	5.68	1.21	AAM22740.1	7e-39
Receptor serine/threonine kinase like protein [*O. sativa* (japonica)]	Contig21786_at	2.00	1.21	BAB84596.1	2e-65

Annotation	Probe ID	Fold change* (Cd treatment *vs* control)		Accession No	E-value
		W6nk2	Zhenong8		
Putative receptor-protein kinase [*O. sativa* (japonica)]	Contig14350_at	2.33	1.22	BAB56062.1	4e-92
Diacylglycerol kinase [*L. esculentum*]	Contig5427_at	3.34	1.38	AAG23129.1	3e-97
Putative wall-associated kinase 1 [*O. sativa*]	Contig11886_s_at	9.25	1.43	AAL76192.1	2e-60
Putative diacylglycerol kinase [*O. sativa* (japonica)]	Contig20753_at	2.10	1.61	BAB92552.1	e-104
Leucine-rich repeat transmembrane protein kinase [*A. thaliana*]	Contig22980_at	2.13	1.84	NP_177451.1	6e-17
Heat stress transcription factor Spl7 [*O. sativa* (japonica)]	Contig18961_at	2.12	-1.32	BAB71737.1	3e-26
Putative receptor protein kinase-like protein [*O. sativa* (japonica)]	Contig24190_at	1.08	-18.7	BAB63567.1	2e-33
**Cell growth, division**					
Similar to Prunus armeniaca ethylene–forming -enzyme -like dioxygenase. [*O. sativa*]	Contig10361_at	3.29	1.46	BAA95829.1	2e-78
Embryogenesis transmembrane protein-like [*O. sativa* (japonica)]	Contig17563_at	4.42	1.47	BAA84620.1	e-30
Auxin-induced protein [*Mesembryanthemum crystallinum*]	Contig21246_at	2.13	1.49	T12211	8e-17
AT4g17280/dl4675c [*A. thaliana*]	HV_CEb0020C01r2_at	4.27	-1.91	AAL57706.1	9e-06
**Protein synthesis**					
Ribosomal protein L17 [*Castanea sativa*]	Contig1908_s_at	2.10	1.34	AAK25758.1	7e-76
40S ribosomal protein S15a-1 [*A. thaliana*]	Contig2522_at	2.00	1.36	NP_172256.1	6e-69
Probable 60S ribosomal protein L9 [*O. sativa subsp.* japonica]	Contig2103_at	2.31	1.51	P49210	5e-85
Anthranilate synthase alpha 2 subunit [*O. sativa* (japonica)]	HY07P02u_at	1.93	-3.93	BAA82095.1	e-67
**Unknown classified**					
Hypothetical protein [*O. sativa*]	HVSMEb0010O13f2_at	8.29	-5.10	CAD39838.1	7e-30
P0470A12.5 [*O. sativa* (japonica)]	Contig5075_at	2.65	-1.65	BAB90280.1	7e-13
Hypothetical protein [*O. sativa* (japonica)]	Contig11664_at	4.23	-1.42	BAB17148.1	6e-36
Unknown protein [*O. sativa*]	Contig12084_at	2.15	-1.42	AAF34415.1	9e-06
Unnamed protein product [*O. sativa* (japonica)]	HV_CEb0017D17f_at	2.50	-1.35	BAA94238.1	e-39
Hypothetical protein [*O. sativa* (japonica)]	Contig13562_at	2.49	-1.34	BAB90026.1	e-49
Hypothetical protein [*A. thaliana*]	Contig7751_at	2.30	-1.32	NP_683570.1	e-57
Hypothetical protein [*H. vulgare*]	Contig634_at	7.66	-1.28	T06204	e-120
Expressed protein [*A. thaliana*]	Contig8464_at	2.13	-1.25	NP_565843.1	2e-83
Hypothetical protein [*H. vulgare*]	Contig590_s_at	9.69	-1.24	T06205	4e-80
Hypothetical protein [*O. sativa* (japonica)]	Contig590_at	7.58	-1.17	T06205	4e-80
Unknown protein [*A. thaliana*]	baak20j05_s_at	2.13	1.05	NP_177760.1	2e-22
Unknown protein [*A. thaliana*]	Contig15548_at	2.36	1.08	NP_181336.1	6e-59
Hypothetical protein [*O. sativa* (japonica)]	Contig16143_at	2.24	1.12	AAM19029.1	9e-85
Putative subtilase [*O. sativa* (japonica)]	Contig8307_s_at	4.01	1.16	BAB89882.1	6e-57

Annotation	Probe ID	Fold change* (Cd treatment *vs* control)		Accession No	E-value
		W6nk2	Zhenong8		
Hypothetical protein [*O.sativa*]	Contig11332_at	2.31	1.36	BAA88541.1	5e-85
Hypothetical protein [*O. sativa* (japonica)]	HO14C15S_at	4.40	1.37	BAC03293.1	3e-17
Putative protein [*A. thaliana*]	HVSMEa0004N20r2_at	2.16	1.47	NP_197387.1	6e-14
Putative uncharacterized protein [*O.sativa* (japonica)]	Contig16375_at	2.17	1.64	BAC16424.1	5e-22
Unknown protein [*Anopheles gambiae str.* PEST]	Contig8492_at	2.66	1.67	EAA12569.1	9e-07
Putative uncharacterized protein [*O. sativa* (japonica)]	Contig25762_at	2.84	1.69	BAC16424.1	4e-22
Unknown protein [*O. sativa* (japonica)]	Contig5075_s_at	3.59	1.70	BAB90280.1	7e-13
Unknown protein [*A. thaliana*]	Contig10480_at	2.80	1.75	NP_177728.1	3e-46
Uncharacterized protein At5g20100.1 [*A.thaliana*]	Contig16397_at	2.30	1.80	NP_197510.1	4e-11
Hypothetical protein [*O. sativa* (japonica)]	Contig11927_at	3.15	1.81	BAB92864.1	9e-11
Hypothetical protein [*O. sativa* (japonica)]	Contig9057_at	3.67	1.86	BAB86120.1	7e-66
Putative uncharacterized protein At5g48370 [*A. thaliana*]	Contig9679_at	2.33	-1.2	NP_199648.1	9e-75
Unknown protein [*O. sativa* (japonica)]	Contig6169_at	2.19	1.00	BAB78620.1	2e-35
Unknown protein [*O. sativa* (japonica)]	Contig11615_s_at	2.56	1.15	BAB63815.1	2e-24
Putative uncharacterized protein [*A.thaliana*]	Contig10152_at	2.32	-1.11	NP_193034.1	3e-59
Unknown protein [*O. sativa* (japonica)]	Contig8851_at	2.24	-1.01	BAB16483.1	e-52
Hypothetical protein [*O. sativa* (japonica)]	HV_CEb0001D02r2_at	3.66	1.18	BAB86123.1	5e-20
Unknown protein [*O. sativa* (japonica)]	Contig11615_at	2.00	1.43	BAB63815.1	2e-24
Putative MAWD binding protein [*O. sativa subsp.* japonica]	Contig9255_at	2.07	-1.07	BAA88529.1	4e-74
Putative protein [*O. sativa* (japonica)]	Contig8722_at	4.54	1.25	BAA90634.1	8e-83
Unknown protein [*O.sativa* (japonica)]	Contig4691_at	2.26	-1.02	BAB64678.1	6e-18
Putative protein OSJNBb0115I21.2 [*O. sativa* (japonica)]	HI02L18u_at	2.33	-1.01	CAD39695.1	4e-48
Hypothetical protein B1146B04.15 [*O. sativa* (japonica)]	Contig2710_s_at	2.03	1.21	BAB64584.1	8e-13
Unknown protein [*A. thaliana*]	Contig10439_at	2.07	1.22	NP_199598.1	2e-39
Putative protein [*A. thaliana*]	Contig7415_at	1.26	-2.2	NP_193723.1	e-103
Hypothetical protein P0003E08.5 [*O. sativa* (japonica)]	HVSMEi0002B05r2_at	-1.21	-2.04	BAB63539.1	3e-07
Putative uncharacterized protein At4g13400 [*A. thaliana*]	Contig7503_at	-1.91	-2.33	AAM97033.1	5e-57
Hypothetical protein [*O. sativa* (japonica)]	Contig7736_at	-1.83	-2.3	BAB62552.1	2e-51
B1131B07.13 [*O. sativa* (japonica)]	Contig10168_at	1.23	-2.38	BAB93351.1	e-23
Expressed protein	Contig15344_at	-1.62	-3.46	NP_564716.1	4e-63
Expressed protein [*A. thaliana*]	Contig15773_at	1.25	-2.3	NP_565890.1	3e-06
Putative uncharacterized protein At4g21930 [*A. thaliana*]	Contig16113_at	-1.85	-2.07	AAM53278.1	4e-11
Unknown protein [*O.sativa* (japonica)]	Contig10905_at	-1.36	-2.37	AAK71559.1	4e-25
Hypothetical protein [*Oenothera elata subsp.* hookeri]	HVSMEc0001M13f_x_at	-1.79	-2.79	NP_084748.1	3e-20
OSJNBa0052O21.28 [*O. sativa* (japonica)]	Contig13248_at	1.92	-2.03	CAD40043.1	e-82

Annotation	Probe ID	Fold change* (Cd treatment vs control)		Accession No	E-value
		W6nk2	Zhenong8		
**None**					
none	HD04G07u_s_at	2.96	-3.51	none	none
none	EBem05_SQ002_D05_s_at	2.97	-2.69	none	none
none	Contig11773_at	8.97	-1.96	none	none
none	Contig17960_at	2.05	-1.91	none	none
none	HB20H10r_at	2.16	-1.88	none	none
none	EBpi07_SQ002_J15_at	3.67	-1.54	none	none
none	Contig13632_at	3.97	-1.50	none	none
none	Contig2499_s_at	3.77	-1.38	none	none
none	Contig8178_at	2.21	-1.38	none	none
none	EBpi01_SQ001_B04_s_at	2.04	-1.36	none	none
none	HVSMEm0001I19r2_at	4.09	-1.35	none	none
none	rbaal31o11_x_at	3.47	-1.34	none	none
none	Contig13218_at	3.19	-1.26	none	none
none	HV_CEa0014D10r2_s_at	2.96	-1.20	none	none
none	Contig16529_at	3.95	-1.09	none	none
none	Contig26496_at	16.49	-1.07	none	none
none	HW01K06u_s_at	2.23	-1.06	none	none
none	HP01B09w_at	6.25	-1.05	none	none
none	Contig17926_at	2.06	-1.04	none	none
none	HVSMEm0003C21r2_at	2.00	-1.03	none	none
none	rbaal30e02_s_at	2.06	-1.01	none	none
none	Contig12794_at	2.51	1.01	none	none
none	Contig2704_s_at	2.98	1.02	none	none
none	Contig12124_at	2.97	1.03	none	none
none	HV14K06u_x_at	3.13	1.04	none	none
none	baak33c23_at	19.55	1.05	none	none
none	EBro08_SQ008_K12_at	3.39	1.05	none	none
none	HVSMEl0005L10f_s_at	2.07	1.05	none	none
none	rbaal20n01_s_at	2.03	1.05	none	none
none	HVSMEf0020A12r2_s_at	2.08	1.08	none	none
none	Contig1185_at	3.44	1.09	none	none
none	Contig14685_at	2.98	1.11	none	none
none	HVCEa0009C05r2_s_at	2.53	1.11	none	none
none	Contig9663_at	3.51	1.12	none	none
none	Contig18427_s_at	3.70	1.14	none	none
none	HVSMEl0025L16f_at	3.27	1.14	none	none
none	EBpi01_SQ004_I24_s_at	2.35	1.14	none	none
none	HZ42B19r_at	2.02	1.14	none	none
none	Contig9663_s_at	2.33	1.15	none	none
none	EBro02_SQ004_C14_at	2.59	1.16	none	none
none	Contig8558_s_at	2.23	1.16	none	none
none	Contig12336_at	2.47	1.17	none	none

Annotation	Probe ID	Fold change* (Cd treatment *vs* control)		Accession No	E-value
		W6nk2	Zhenong8		
none	Contig1159_s_at	2.35	1.20	none	none
none	Contig12700_at	2.05	1.20	none	none
none	Contig7315_at	2.33	1.21	none	none
none	Contig6310_at	4.15	1.24	none	none
none	HO14I22S_s_at	2.60	1.25	none	none
none	HVSMEb0005E07r2_at	2.10	1.26	none	none
none	EBem10_SQ003_N11_at	3.24	1.27	none	none
none	S0001000055P18F1_s_at	5.01	1.29	none	none
none	Contig19265_at	4.26	1.33	none	none
none	HY08G17u_s_at	2.40	1.33	none	none
none	baak4a13_at	5.36	1.34	none	none
none	EBpi01_SQ002_L02_x_at	2.03	1.35	none	none
none	Contig4413_s_at	2.02	1.44	none	none
none	Contig5303_at	3.75	1.45	none	none
none	Contig17275_at	3.79	1.49	none	none
none	Contig14134_at	2.14	1.53	none	none
none	HY09L01u_s_at	2.12	1.53	none	none
none	Contig13615_at	2.54	1.55	none	none
none	EBem10_SQ002_L14_s_at	2.73	1.59	none	none
none	HK06M02r_at	2.36	1.59	none	none
none	EBpi01_SQ002_L02_at	2.14	1.59	none	none
none	HV_CEb0009D09r2_at	14.69	1.74	none	none
none	Contig4031_x_at	2.14	1.91	none	none
none	Contig9222_at	1.06	-3.06	none	none
none	Contig11968_at	1.01	-2.57	none	none
none	Contig14528_at	1.28	-3.37	none	none
none	Contig14915_at	1.94	-2.05	none	none
none	Contig12195_at	1.86	-2.23	none	none
none	Contig19960_s_at	-1.51	-3.71	none	none
none	HVSMEc0001A15f_at	-1.52	-2.33	none	none
none	baak4c06_at	-1.57	-3.08	none	none
none	HVSMEi0020G22r2_at	-1.84	-2.1	none	none
none	HX02B15u_s_at	-1.18	-2.85	none	none
none	rbaal38f16_at	-1.43	-2.05	none	none
