# Supplementary material for: DNA microarray revealed and RNAi plants confirmed key genes conferring low Cd accumulation in barley grains
Source: BMC Plant Biol. 2015 Oct 26;15:259. doi: 10.1186/s12870-015-0648-5 (PMC4623906; doi:10.1186/s12870-015-0648-5)
Supplement: Additional file 7: Table S2. — List of genes up-regulated in Zhenong8 and down-regulated or no change in W6nk2, or no change in Zhenong8 and down-regulated in W6nk2 after 15 days exposure to 5 μM Cd. (PDF 152 kb) [file 12870_2015_648_MOESM7_ESM.pdf]

Table S2 List of genes down- and up-regulated, no change and up- regulated, down-regulated and no change in W6nk2 and Zhenong8 respectively after 15 days exposure to 5 µM Cd.

Annotation	Probe ID	Fold change (Cd treatment *vs* control) W6nk2	Fold change (Cd treatment *vs* control) Zhenong8	Accession No	E-value
**Stress and defense response**					
Similar to Babesia aldo-keto reductase [*A. thaliana*]	HVSMEm0020M20r2_s_at	-1.52	2.49	AAB70433.1	4e-05
Quinone oxidoreductase-like protein [*A. thaliana*]	Contig5658_at	-1.47	2.19	Q9ZUC1	9e-90
FKBP-type peptidyl-prolyl cis-trans isomerase 6 [*A. thaliana*]	Contig10930_s_at	-1.42	2.04	NP_567098.1	2e-34
Putative heat shock protein [*A. thaliana*]	Contig4554_at	-1.39	2.14	AAM65596.1	3e-46
Putative sugar-starvation induced protein [*O. sativa* (japonica)]	Contig4954_s_at	1.30	2.15	AAL83638.1	7e-05
Cadmium-induced protein-like [*O. sativa* (japonica)]	Contig6664_at	1.69	2.12	BAC19956.1	6e-58
Putative L-ascorbate peroxidase, chloroplast precursor [*Solanum lycopersicum*]	Contig8515_s_at	-1.44	2.06	Q9THX6	3e-64
Similar to Babesia aldo-keto reductase [*A. thaliana*]	Contig9187_at	-1.39	2.51	AAB70433.1	5e-94
Bundle sheath defective protein 2 [*Z. mays*]	Contig9809_at	-1.42	2.29	AAD28599.1	3e-39
Putative stress inducible protein [*O. sativa* (japonica)]	Contig11554_at	1.52	2.35	AAM93720.1	9e-84
Dehydrin 7 [*H. vulgare*]	Contig1709_at	1.07	2.50	AAD02258.1	5e-28
Putative cytochrome P450 [*Lolium rigidum*]	HVSMEm0003G16r2_at	-1.22	2.68	AAK38084.1	3e-57
Putative cytochrome P450 [*O. sativa* (japonica)]	Contig12508_s_at	1.08	2.00	AAN05337.1	2e-46
Putative cytochrome P450 [*O. sativa*]	Contig14534_at	1.70	2.63	AAL73064.1	e-44
Putative cinnamoyl-CoA reductase [*A. thaliana*]	Contig8979_at	-1.16	2.05	NP_180917.1	3e-67
Putative lycopene epsilon-cyclase [*O. sativa* (japonica)]	Contig14290_at	-1.08	2.22	BAC05562.1	e-118
Avr9/Cf-9 rapidly elicited protein 65 [*N. tabacum*]	Contig19890_at	1.88	5.93	AAG43557.1	e-04
20 kDa chaperonin [*A. thaliana*]	Contig3840_at	1.03	3.12	NP_197572.1	5e-87
Chloroplast 20 kDa chaperonin [*A. thaliana*]	Contig10790_at	1.01	3.06	NP_197572.1	e-78
PRLI-interacting factor L [*A. thaliana*]	Contig14905_s_at	-1.03	2.05	NP_173025.1	3e-37
S47087 pir7b protein [*O. sativa*]	Contig10057_at	1.57	3.12	Q43360	3e-83

Annotation	Probe ID	Fold change (Cd treatment *vs* control)		Accession No	E-value
		W6nk2	Zhenong8		
Ferritin 1, chloroplast precursor [*Z. mays*]	Contig2714_at	-2.41	-1.68	P29036	3e-71
Ferritin [*O. sativa* (japonica)]	HV12A05u_s_at	-2.76	-1.56	AAM74943.1	4e-34
Ferritin [*O. sativa* (japonica)]	Contig2716_s_at	-2.32	-1.44	AAM74942.1	3e-79
DNA protein - like; protein id: At5g03030.1 [*A. thaliana*]	Contig11141_at	-2.09	-1.00	NP_195923.1	e-37
ABA-inducible protein WRAB1 [*T. aestivum*]	Contig2406_at	-2.09	1.11	AAD33850.1	e-56
**Transport**					
AT5g14910/F2G14_30 [*A. thaliana*]	Contig10306_at	-2.26	4.28	NP_568306.1	3e-23
AT5g14910/F2G14_30 [*A. thaliana*]	Contig10306_s_at	-2.39	4.09	NP_568306.1	3e-23
Vacuolar ATP synthase 16 kDa proteolipid subunit H^+-ATPase [*O. sativa* (japonica)]	HA10P21u_at	-3.19	2.08	Q40635	2e-29
Tic62 protein [*Pisum sativum*]	rbasd23b03_s_at	-1.17	2.13	CAC87810.2	0.004
Glutathione transferase [*H. vulgare subsp.*]	Contig20831_at	-1.51	3.08	AAL73394.1	5e-60
Herbicide safener binding protein SBP1 [*Z.mays*]	Contig3552_at	-1.32	3.26	T01354	3e-51
Herbicide safener binding protein 1 [*Z. mays*]	Contig4910_at	1.24	4.6	T01354	4e-62
Herbicide safener binding protein 1 [*Z. mays*]	Contig3552_s_at	-1.10	4.34	T01354	3e-51
Uracil phosphoribosyltransferase-like protein [*A. thaliana*]	Contig19273_at	-1.54	2.78	NP_190958.1	9e-26
Putative ribulose-1,5-bisphosphate carboxylase oxygenase small subunit N-methyltransferase I [*A. thaliana*]	HVSMEa0009E14r2_at	-1.03	2.17	NP_187424.1	3e-17
Nucleoside diphosphate kinase II [*Spinacia oleracea*]	Contig7963_at	-1.35	2.59	Q01402	2e-64
ATP sulfurylase, putative [*A. thaliana*]	Contig14398_s_at	1.01	2.37	AAM63185.1	3e-12
Putative endoxyloglucan transferase [*O. sativa*]	Contig7337_at	1.55	2.00	AAL58186.1	8e-86
Naringenin-chalcone synthase 1 [*H. vulgare*]	Contig7356_at	-1.10	2.35	P26018	e-88
Naringenin-chalcone synthase 1 [*Secale cereale*]	Contig7358_at	-1.09	2.23	P53414	6e-92
Glu-tRNAamidotransferase subunit A [*A. thaliana*]	Contig7597_at	-1.14	2.81	AAG29095.1	9e-95
P0529E05.24 [*O. sativa* (japonica)]	Contig9863_at	1.99	2.55	BAB84408.1	5e-79
Mitochondrial import receptor subunit TOM7-1 [*S. tuberosum*]	Contig11562_at	-1.04	2.16	T07681	7e-13
Putative lipid transfer protein [*O. sativa* (japonica)]	Contig11884_at	1.42	7.33	AAN05564.1	5e-14

Annotation	Probe ID	Fold change (Cd treatment *vs* control)		Accession No	E-value
		W6nk2	Zhenong8		
Glu-tRNA(Gln) amidotransferase subunit C [*A. thaliana*]	Contig16053_at	-1.40	3.48	AAG29097.1	3e-24
3-oxoacyl-[acyl-carrier-protein] synthase I [*H. vulgare*]	Contig18583_at	-1.71	2.00	P23902	2e-94
Aquaporin 2 [*Samanea saman*]	Contig19393_at	-1.64	2.53	AAC17529.1	7e-57
THA4 [*Z. mays*]	Contig18499_at	1.18	2.51	AAD31522.1	5e-18
Glu-tRNA(Gln) amidotransferase subunit C [*A. thaliana*]	HK0e08r_s_at	-1.60	3.59	AAG29097.1	2e-04
LL-diaminopimelate aminotransferase [*A. thaliana*]	Contig9314_at	1.45	2.33	NP_567934.1	4e-65
Calcium-dependent protein kinase 29 [*A. thaliana*]	Contig10834_at	-1.09	3.70	AAF26765.1	3e-78
Probable FKBP-type peptidyl-prolyl cis-trans isomerase 4 [*A. thaliana*]	Contig21540_at	-1.77	2.79	AAK97696.1	7e-05
Putative integral membrane protein [*A. thaliana*]	Contig17740_at	1.02	2.66	NP_178451.1	e-55
Glyoxylate aminotransferase 2 homolog [*A. thaliana*]	Contig5255_at	-2.14	-1.38	AAD48837.1	e-96
Amino acid selective channel protein [*H. vulgare subsp.*]	Contig3789_s_at	-2.15	1.58	CAA09867.1	3e-68
Phytoene synthase, chloroplast precursor [*Z. mays*]	Contig13305_at	-2.18	1.61	P49085	7e-42
Os06g0141200 protein [*O. sativa*]	Contig12472_at	-2.86	-1.51	BAA83556.1	8e-61
**Transcription**					
Cp10-like protein [*Gossypium hirsutum*]	Contig401_at	1.35	2.23	AAM77651.1	2e-78
Phenylalanine-tRNA synthetase-like protein [*A. thaliana*]	Contig8405_at	1.08	2.03	NP_567061.1	e-100
SKP1 interacting partner 1 (SKIP1) [*A. thaliana*]	Contig19249_at	1.74	2.00	NP_568870.1	4e-45
FKBP-type peptidyl-prolyl cis-trans isomerase [*A. thaliana*]	Contig19209_at	-1.37	2.01	NP_173504.1	7e-39
Chloroplast RNA-binding protein AT1G60000 [*A. thaliana*]	Contig12973_at	1.03	2.72	NP_176208.1	3e-69
Probable RNA-binding protein cp33 precursor [*H. vulgare subsp.*]	Contig7834_at	1.56	4.52	T05730	e-153
Putative ribonucleoprotein [*O. sativa*]	Contig5988_at	1.23	7.24	AAL82527.1	6e-76
Cp31AHv protein [*H. vulgare*]	rbasd3a10_s_at	-1.21	2.43	T05725	3e-46
DNA-binding protein ABF2 [*Avena fatua*]	Contig4386_at	-1.45	2.76	S61414	2e-92
Putative ribonucleoprotein [*O. sativa* (japonica)]	Contig4432_at	1.82	2.93	BAC10140.1	3e-92
Peptidyl-prolyl cis-trans isomerase CYP20-2 [*A. thaliana*]	Contig4537_at	-1.05	2.24	NP_196816.1	4e-71
Peptidyl-prolyl cis-trans isomerase CYP20-2	Contig4537_s_at	-1.05	2.28	NP_196816.1	4e-71

Annotation	Probe ID	Fold change (Cd treatment *vs* control)		Accession No	E-value
		W6nk2	Zhenong8		
[*A.thaliana*]					
Putative nucleosome assembly protein [*A. thaliana*]	Contig4668_at	1.11	2.17	NP_179538.1	e-74
Trigger factor-like protein [*A. thaliana*]	Contig7069_at	-1.63	3.65	NP_200333.2	2e-79
Putative GTP-binding protein [*O. sativa* (japonica)]	Contig7575_at	-1.53	3.28	AAK09228.1	e -106
Putative mRNA binding protein precursor [*O.sativa*]	Contig8775_at	-1.30	2.21	BAC16410.1	3e-81
DNA-binding protein p24 [*S. tuberosum*]	Contig10233_at	-1.30	2.03	AAF91282.1	5e-19
Peptidyl-prolyl cis-trans isomerase [*A. thaliana*]	Contig11815_at	-1.09	2.00	CAB06699.1	2e-79
Putative MAR-binding protein MFP1 [*O. sativa* (japonica)]	Contig13824_at	-1.30	2.81	BAB33019.1	3e-34
Transducin-like enhancer protein 4 [*Mus musculus*]	Contig13990_at	-1.27	3.12	Q62441	0.029
Peptidylprolyl isomerase [*A. thaliana*]	Contig14849_at	-1.53	2.18	NP_567750.1	2e-38
Putative sun (Fmu) protein [*A. thaliana*]	EBma01_SQ005_J20_at	-1.14	2.04	NP_187924.2	0.036
OSJNBa0072F16.15[*O. sativa*(japonica)]	HV05C10u_s_at	-1.82	2.13	CAD40991.1	e-40
Glutamyl-tRNA synthetase [*H.vulgare*]	Contig11355_at	-1.34	2.16	Q43768	e-124
Glutamyl-tRNA synthetase [*H. vulgare*]	Contig11355_s_at	-1.16	2.15	Q43768	e -124
Putative uncharacterized protein [*O. sativa* (japonica)]	Contig3291_at	1.44	2.60	BAB89768.1	e -109
Os01g0510600 protein [*O. sativa* (japonica)]	Contig7279_at	-1.87	5.21	BAB92188.1	2e-86
P0520B06.12 [*O. sativa* (japonica)]	HVSMEg0001H24r2_s_at	-1.91	6.41	BAB92188.1	6e-13
33 kDa secretory protein [*O. sativa*]	Contig20580_at	1.75	3.39	AAC36744.1	4e-40
MFL8.1/MFL8.1 [*A. thaliana*]	Contig18921_at	1.07	2.56	AAM10318.1	e-14
Sigma factor SIG2B; ZmSIG2B [*Z. mays*]	Contig11428_at	1.03	2.01	AAD17855.1	6e-75
Histone H1-like protein HON101 [*Z. mays*]	Contig2258_at	-3.21	-1.93	AAM93216.1	e-24
histone H2A.2 [*T. aestivum*]	Contig671_at	-2.23	-1.86	S53518	4e-52
Putative myb-related protein [*O. sativa* (japonica)]	Contig18051_at	-2.35	-1.77	AAL87171.1	8e-17
DNA-directed RNA polymerase subunit alpha [*H. vulgare*]	Contig22529_at	-2.29	-1.72	P92392	4e-92
Histone H2B.2 [*T. aestivum*]	Contig1161_at	-2.24	-1.66	P05621	8e-64
Histone H3 [*T. aestivum*]	Contig657_s_at	-2.45	-1.63	P02300	5e-69

Annotation	Probe ID	Fold change (Cd treatment *vs* control) W6nk2	Zhenong8	Accession No	E-value
Histone H2B-8 [*T. aestivum*]	Contig1169_at	-2.72	-1.61	S56685	9e-50
Histone H2A [*Cicer arietinum*]	Contig119_at	-2.61	-1.39	O65759	5e-41
Putative snRNP splicing factor [*A. thaliana*]	Contig5815_at	-4.49	-1.25	NP_178480.1	8e-41
Histone H2B153 [T. aestivum]	Contig1164_at	-2.54	-1.20	S56687	3e-53
Oj000126_13.17 [*O. sativa* (japonica)]	Contig17756_at	-2.04	-1.04	CAD40595.1	5e-52
AT5g51720/MIO24_14 [*A. thaliana*]	Contig14376_at	-2.76	1.03	NP_568764.1	5e-26
**Signal transduction**					
Putative 32.7 kDa jasmonate-induced protein [*H. vulgare*]	HVSMEm0015P16r2_at	1.35	2.75	T04375	5e-10
Phosphoenolpyruvate carboxykinase (ATP) [*A. Thaliana*]	Contig6435_at	-2.04	-1.39	NP_195500.1	e-117
Putative protein kinase [*S. bicolor*]	Contig10013_at	-2.16	-1.35	AAM47583.1	e-114
Invertase inhibitor homolog [*A. Thaliana*]	bags7h06_at	-2.11	-1.30	NP_201267.1	9e-06
Similar to senescence-associated family protein [*N. tabacum*]	Contig15259_at	-3.90	1.04	BAA88985.2	3e-37
**Carbohydrate metabolism**					
Alcohol dehydrogenase [*T. aestivum*]	Contig393_at	1.75	3.61	A61024	e-132
Glyceraldehyde-3-phosphate dehydrogenase B [*O. sativa* (japonica)]	Contig446_at	-1.08	2.15	BAA85402.1	e-113
Glucan endo-1,3-beta-glucosidase [*H.vulgare subsp.*]	Contig1632_at	1.01	2.07	AAA32960.1	e-169
Adenosine diphosphate glucose pyrophosphatase [*H. vulgare subsp.*]	Contig2769_s_at	-1.74	14.55	CAC32847.1	4e-72
Alpha-galactosidase [*Oryza sativa* (japonica)]	Contig4187_at	1.03	2.67	BAB12570.1	e-126
ATP-dependent Clp protease proteolytic subunit [*A. thaliana*]	Contig4497_at	1.24	2.10	NP_563907.1	2e-75
Putative 3-isopropylmalate dehydrogenase [*H. vulgare*]	Contig5555_at	-1.02	2.03	NP_178171.1	e-105
Putative 3-beta hydroxysteroid dehydrogenase/ isomerase protein [*O. sativa*]	Contig6963_at	-1.74	2.47	AAK73149.1	e-101
Putative amylase [*Oryza sativa* (japonica)]	Contig8246_at	1.24	2.37	AAK27799.1	e-93
At2g42220/T24P15.13 [*Arabidopsis thaliana*]	Contig9878_at	-1.19	2.03	AAK73974.1	e-62
Putative nodulin [*Oryza sativa* (japonica)]	Contig10919_s_at	-1.05	2.02	BAB17350.1	5e-29
Putative nascent polypeptide associated complex alpha chain [*O.*	Contig11109_at	-1.09	4.71	AAM52321.1	2e-46

Annotation	Probe ID	Fold change (Cd treatment *vs* control)		Accession No	E-value
		W6nk2	Zhenong8		
sativa]					
Putative GTP-binding protein [*Streptococcus pyogenes serotype M3*]	Contig14490_at	1.48	3.85	NP_664814.1	7e-40
Glyceraldehyde-3-phosphate dehydrogenase [*A. thaliana*]	baak1k18_s_at	-1.12	2.04	NP_174996.1	2e-05
Putative esterase D [*Oryza sativa* (japonica)]	HV12A17u_s_at	1.85	2.21	BAB90254.1	e-36
ATP-dependent Clp protease proteolytic subunit [*A. thaliana*]	Contig5768_at	1.30	2.02	NP_563836.1	2e-57
ATP-dependent Clp protease proteolytic subunit [*A. thaliana*]	Contig6692_s_at	-1.17	2.35	NP_564560.1	8e-34
Putative uncharacterized protein T8P19.210 [*A. thaliana*]	Contig5704_at	-1.32	2.74	NP_190439.1	7e-44
MRNA, complete cds, clone: RAFL22-43-L07 [*A. thaliana*]	Contig23996_at	-1.66	2.04	NP_568077.1	6e-49
Xyloglucan endo-1,4-beta-D-glucanase [*Z. mays*]	Contig2672_at	-2.07	-1.42	T02090	2e-64
Xyloglucan endo-1,4-beta-D-glucanase [*H. vulgare*]	Contig2671_at	-2.73	-1.79	T06202	e-160
Cyclic phosphodiesterase [*A. Thaliana*]	Contig14273_at	-2.06	-1.34	1JH7	9e-35
**Fat metabolism**					
Seed storage protein, 35K isoform AmA1 [*Amaranthus hypochondriacus*]	Contig3533_at	-1.09	3.43	S24263	2e-14
Fatty acyl coA reductase [*T. aestivum*]	Contig10274_at	1.18	2.63	CAD30694.1	e-59
GDSL esterase/lipase CPRD49 [*A.thaliana*]	Contig13508_at	1.03	2.35	AAM63310.1	4e-71
Lipase-like protein [*O. sativa* (japonica)]	Contig20235_s_at	-2.54	1.37	BAB89205.1	2e-19
**Nitrogen metabolism**					
Putative aspartate transaminase [*O. sativa* (japonica)]	Contig4244_at	-1.04	3.35	BAB63467.1	6e-93
F23N19.15 [*A. thaliana*]	Contig6504_s_at	-1.27	2.33	AAF19544.1	e-37
**Photosynthesis**					
Chlorophyll a/b-binding protein WCAB precursor [*T. aestivum*]	Contig422_at	-3.79	6.77	AAB18209.1	e-132
Ribulose-1,5-bisphosphate carboxylase/oxygenase small subunit [*T. aestivum*]	Contig842_x_at	-2.44	3.72	BAB19811.1	9e-97
Ribulose-bisphosphate carboxylase [*T.*	Contig997_x_at	-2.49	3.36	RKWTS	4e-88

Annotation	Probe ID	Fold change (Cd treatment *vs* control)		Accession No	E-value
		W6nk2	Zhenong8		
aestivum]					
Chlorophyll A-B binding protein 3A [*S. lycopersicum*]	Contig960_s_at	-2.66	3.23	P14276	6e-19
Chlorophyll a/b-binding protein WCAB precursor [*T. aestivum*]	Contig949_at	-2.94	3.12	AAB18209.1	e-125
Chlorophyll a/b-binding protein WCAB precursor [*T. aestivum*]	Contig347_s_at	-4.0	2.58	AAB18209.1	6e-94
Chlorophyll a/b-binding protein WCAB precursor [*T. aestivum*]	Contig418_at	-5.48	2.58	AAB18209.1	e-128
Chlorophyll a/b-binding protein WCAB precursor [*T. aestivum*]	Contig841_x_at	-2.32	2.33	AAB18209.1	e-120
Ribulose-1,5-bisphosphate carboxylase/oxygenase small subunit [*H. vulgare subsp.* vulgare]	Contig1004_x_at	-3.36	2.17	BAA35162.1	7e-80
Ribulose-1,5-bisphosphate carboxylase/ oxygenase small subunit [*T. aestivum*]	Contig497_s_at	-1.15	2.06	BAB19812.1	6e-60
Ribulose bisphosphate carboxylase small chain [*T. aestivum*]	Contig594_x_at	-1.49	2.02	P26667	5e-97
RuBisCO large subunit-binding protein subunit beta [*S. cereale*]	Contig807_at	1.60	3.86	Q43831	4e-98
RuBisCO large subunit-binding protein subunit beta [*S. cereale*]	Contig807_s_at	1.63	3.22	Q43831	4e-98
Ferredoxin-thioredoxin reductase [*Z. mays*]	Contig2399_at	-1.14	2.00	P41347	7e-15
RuBisCO large subunit-binding protein subunit alpha [*T. aestivum*]	rbags36a18_s_at	1.40	20.53	P08823	5e-08
NADPH-protochlorophyllide oxidoreductase B [*H. vulgare*]	Contig2766_s_at	-1.02	2.05	Q42850	2e-54
Mg-chelatase subunit XANTHA-F [*H. vulgare subsp.*]	Contig2985_s_at	-1.21	2.74	AAK72401.1	e-139
Protoporphyrin IX magnesium chelatase subunit [*H. vulgare*]	Contig5341_at	-1.24	2.48	S64722	e-167
Coproporphyrinogen III oxidase [*H. vulgare*]	Contig5401_s_at	-1.00	3.30	Q42840	e-110
Porphobilinogen deaminase [*T. aestivum*]	Contig5956_at	1.39	3.19	AAL12220.1	e-122
S71747 DAG protein [*Antirrhinum majus*]	Contig7509_at	-1.08	2.31	Q38732	5e-68
Putative chloroplast inner envelope protein [*O. sativa*]	Contig7645_at	1.25	2.12	AAG13554.1	e-109
Putative protoporphyrinogen IX oxidase [*O. sativa*(japonica)]	Contig7919_at	-1.01	3.48	BAB39998.1	e-115
Putative phytochrome-associated protein [*O. sativa* (japonica)]	Contig8115_s_at	1.46	2.00	BAB91924.1	e-71

Annotation	Probe ID	Fold change (Cd treatment *vs* control)		Accession No	E-value
		W6nk2	Zhenong8		
Putative uroporphyrinogen decarboxylase [*O. sativa* (japonica)]	Contig8595_at	-1.51	2.42	BAB21078.1	e-113
Thylakoid lumen 15.0-kDa protein [*A. thaliana*]	Contig9582_at	-1.38	2.44	NP_568781.1	5e-72
Mg-protoporphyrin IX [*H. vulgare*]	Contig10699_at	-1.41	2.33	CAB58179.1	e-151
Putative ribulose-1,5 bisphosphate carboxylase oxygenase large subunit N-methyltransferase [*A. thaliana*]	Contig11083_at	-1.15	2.35	NP_172856.1	3e-81
PsbP-related thylakoid lumenal protein 1 [*A. thaliana*]	Contig14611_at	-1.70	2.33	NP_567468.1	3e-56
NADPH-protochlorophyllide oxidoreductase B [*H. vulgare*]	Contig2762_at	-1.11	2.25	Q42850	e-129
Ribulose-bisphosphate carboxylase small chain precursor [*T. aestivum*]	HVSMEm0020L23r2_x_at	-1.76	2.35	RKWTS	e-36
Thylakoid lumen pentapeptide repeat family protein [*A. thaliana*]	HVSMEa0012N07r2_at	-1.32	2.17	NP_566030.1	e-51
Putative phytochrome-associated protein [*O. sativa* (japonica)]	HV_CEb0024B09r2_s_at	1.83	3.17	BAB91924.1	e-19
Ribulose-1,5-bisphosphate carboxylase/ oxygenase small subunit [*T. aestivum*]	HVSMEa0011D13r2_at	-1.59	2.28	BAB19814.1	9e-17
PsbQ domain protein family, F7A19.23 protein [*A. thaliana*]	Contig15111_at	-1.51	2.01	NP_563937.1	2e-43
PsbP-related thylakoid lumenal protein 4 [*A. thaliana*]	Contig11175_s_at	1.05	2.13	NP_196706.2	5e-60
Lil3 protein [*A. thaliana*]	Contig2314_at	-1.06	2.60	AAM63936.1	2e-49
Trehalose-6-phosphate phosphatase [*A. Thaliana*]	Contig24583_at	-2.06	-1.18	NP_199959.1	3e-35
Chlorophyll a/b-binding protein WCAB precursor [*T. aestivum*]	Contig617_x_at	-4.16	-1.06	AAB18209.1	e-88
Chlorophyll a/b-binding protein type I [*H. vulgare subsp.*]	X89023_x_at	-2.08	-1.02	T05938	e-153
Chlorophyll A-B binding protein 25, [*Petunia sp.*]	baak16l04_x_at	-2.32	1.01	P04782	3e-37
Chlorophyll a/b-binding protein WCAB precursor [*T. aestivum*]	Contig6_x_at	-2.47	1.04	AAB18209.1	e-122
Chlorophyll a/b-binding protein precursor [*H. vulgare*]	HVSMEn0020J05f_x_at	-2.10	1.22	AAF90200.1	3e-28
Ribulose-bisphosphate carboxylase [*T. aestivum*]	Contig589_x_at	-2.17	1.31	RKWTS	3e-97
Chlorophyll a/b-binding protein WCAB precursor [*T. aestivum*]	Contig1012_s_at	-2.87	1.32	AAB18209.1	4e-79
Chlorophyll a/b-binding protein WCAB precursor [*T. aestivum*]	Contig432_x_at	-4.01	1.32	AAB18209.1	e-128
Chlorophyll a/b-binding protein WCAB	Contig425_at	-6.37	1.34	AAB18209.1	e-113

Annotation	Probe ID	Fold change (Cd treatment *vs* control)		Accession No	E-value
		W6nk2	Zhenong8		
precursor [*T. aestivum*]					
Chlorophyll a/b-binding protein WCAB precursor [*T. aestivum*]	Contig837_x_at	-2.13	1.35	AAB18209.1	e-127
Chlorophyll a/b-binding protein WCAB precursor [*T. aestivum*]	Contig828_s_at	-2.4	1.69	AAB18209.1	e-138
Chlorophyll A-B binding protein 1B [*L.esculentum*]	Contig433_x_at	-5.22	1.70	1204205B	e-110
**Protein synthesis**					
30S ribosomal protein S17 [*O. sativa*]	Contig4490_s_at	-2.01	3.82	Q9ZST1	6e-45
50S ribosomal protein L24 [*A. thaliana*]	Contig6148_at	-2.05	3.25	NP_200271.1	3e-55
Putative elongation factor P[*A. thaliana*]	Contig17155_at	-2.38	3.06	NP_566333.1	5e-74
Putative 50S ribosomal protein L34 [*O. sativa* (japonica)]	Contig5102_s_at	-2.0	2.78	BAB92266.1	7e-34
30S ribosomal protein 3, chloroplastic [*H. vulgare*]	Contig6675_at	-2.21	2.16	O48609	5e-62
Putative ribosomal protein L18 [*O. sativa*]	Contig5586_at	-2.00	3.29	AAL79739.1	3e-62
30S ribosomal protein S1, chloroplast precursor [*S. oleracea*]	Contig428_at	-1.35	2.68	P29344	e-97
Ribosomal protein L1 protein [*A. thaliana*]	Contig466_at	-1.18	2.26	T51934	e-101
Putative plastid ribosomal protein L19 precursor [*O. sativa*]	Contig2941_at	-1.77	3.08	CAC39039.1	4e-60
Putative ribosomal protein L28 [*O. sativa*]	Contig4380_s_at	-1.80	3.69	AAG03094.1	7e-36
Ribosomal protein S5 [*S. oleracea*]	Contig4439_at	-1.70	2.48	CAA63650.1	2e-55
Ribosomal protein L3 precursor, chloroplast [*N. tabacum*]	Contig5240_at	-1.71	3.34	T01736	e-93
Putative plastid ribosomal protein CL9 [*T. aestivum*]	Contig5492_at	-1.86	2.27	AAM92711.1	4e-89
30S ribosomal protein S13, chloroplastic [*A. thaliana*]	Contig5526_s_at	-1.57	2.42	NP_568299.1	9e-46
Putative chloroplast 50S ribosomal protein L6 [*A. thaliana*]	Contig5573_at	-1.37	2.23	NP_172011.1	7e-82
Putative ribosomal protein L18 [*O. sativa*]	Contig5585_s_at	-1.83	3.68	AAL79739.1	5e-62
Plastid-specific ribosomal protein 6 precursor [*S. oleracea*]	Contig5659_at	-1.33	3.13	AAF64189.1	5e-20
Plastid ribosomal protein CL15 [*A. thaliana*]	Contig5680_at	-1.62	2.67	CAA77592.1	6e-53
Plastid ribosomal protein CL15 [*A. thaliana*]	Contig5680_s_at	-1.48	2.56	CAA77592.1	6e-53

Annotation	Probe ID	Fold change (Cd treatment *vs* control)		Accession No	E-value
		W6nk2	Zhenong8		
30S ribosomal protein S31 [*A. thaliana*]	Contig5708_at	-1.47	2.50	NP_181349.1	8e-10
50S ribosomal protein L5 [*O. sativa*]	Contig5775_at	-1.89	3.45	AAC64970.1	e-106
50S ribosomal protein L5 [*O. sativa*]	Contig5776_s_at	-1.31	2.16	AAC64970.1	3e-42
Putative ribosomal protein L13 [*O. sativa* (japonica)]	Contig6936_at	-1.60	3.45	BAB56046.1	e-105
Plastid ribosomal protein L11 [*O. sativa* (japonica)]	Contig8084_at	-1.96	3.82	BAB21483.1	6e-77
50S ribosomal protein L12-1, chloroplastic [*S. cereale*]	Contig8125_at	-1.59	3.42	Q06030	7e-46
50S ribosomal protein L27 [*O. sativa*(japonica)]	Contig8437_at	-1.67	2.27	O65037	6e-75
30S plastid ribosomal protein S6 [*A. thaliana*]	Contig8956_at	-1.69	2.16	NP_176632.1	2e-44
50S ribosomal protein L35 precursor [*S. oleracea*]	Contig9274_at	-1.14	3.04	P23326	e-28
50S ribosomal protein L35 precursor [*S. oleracea*]	Contig9274_s_at	-1.14	2.25	P23326	e-28
Plastid-specific ribosomal protein 2 precursor [*S. oleracea*]	Contig9436_at	-1.81	6.48	AAF64167.1	3e-50
Ribosomal protein L29 [*Z. mays*]	Contig9437_at	-1.85	3.99	AAD50383.1	2e-50
9S ribosomal protein [*Z. mays*]	Contig10093_s_at	-1.53	2.76	AAK16543.1	2e-64
Ribosomal protein L17-like protein [*A. thaliana*]	Contig10356_at	-1.60	3.40	AAM63452.1	4e-57
Ribosomal protein L12.1 precursor [*S. cereale*]	Contig12793_at	-1.68	3.95	S30199	2e-53
50S ribosomal protein L3 precursor [*N. tabacum*]	HA28J12r_s_at	-1.56	2.50	T01736	0.013
Ribosomal protein L17-like protein [*A. thaliana*]	rbags18k24_s_at	-1.62	3.33	AAM63452.1	2e-15
Ribosomal protein L17-like protein [*A. thaliana*]	rbags18k24_x_at	-1.74	3.43	AAM63452.1	2e-15
50S ribosomal protein L4 [*A. thaliana*]	Contig9938_at	-1.78	3.38	AAF79563.1	5e-78
30S ribosomal protein S20 [*O. sativa* (japonica)]	Contig4215_at	-1.62	3.11	BAB90029.1	9e-62
Ribosome recycling factor, chloroplast precursor [*S. oleracea*]	Contig5004_at	-3.39	1.80	P82231	2e-71
**Unknown classified**					
Unknown protein [*A. thaliana*]	Contig4011_at	-4.37	2.35	AAM97054.1	6e-97
Unknown protein [*O. sativa subsp. japonica*]	Contig5712_at	-1.99	2.09	BAB62639.1	e-88
Unknown protein [*O. sativa* (japonica)]	HVSMEm0004N19r2_s_at	-1.28	2.61	BAB90029.1	3e-17
OSJNBb0066J23.1 [*O. sativa*	Contig16214_at	-1.31	2.62	CAD40597.1	3e-52

Annotation	Probe ID	Fold change (Cd treatment *vs* control)		Accession No	E-value
		W6nk2	Zhenong8		
(japonica)]					
Hypothetical protein [*O. sativa* (japonica)]	HVSMEb0015P10r2_at	-1.49	2.34	BAB39880.1	6e-11
Hypothetical protein [*A. thaliana*]	Contig3396_s_at	-1.37	2.57	T12970	3e-11
Expressed protein [*A. thaliana*]	Contig3659_at	-1.00	4.58	NP_567820.1	2e-54
Expressed protein [*A. thaliana*]	Contig3659_s_at	1.08	5.51	NP_567820.1	2e-54
ESTs AU070372(S13446) [*A. thaliana*]	Contig4243_at	-1.05	3.29	BAA82377.1	7e-74
Expressed protein [*A. thaliana*]	Contig4700_at	-1.15	2.19	NP_567209.1	2e-62
Unknown protein [*O. sativa subsp.* japonica]	Contig5048_s_at	-1.07	2.02	BAB61215.1	5e-35
Unknown protein [*A. thaliana*]	Contig5364_at	1.80	2.11	NP_194537.1	2e-45
Hypothetical protein [*Nostoc sp.* PCC 7120]	Contig6063_s_at	-1.36	7.15	NP_487053.1	9e-34
Pentatricopeptide repeat-containing protein [*A. thaliana*]	Contig6600_at	1.06	3.21	AAM19786.1	2e-50
Unnamed protein [*O. sativa* (japonica)]	Contig6817_at	-1.32	2.41	BAA89561.1	9e-13
Hypothetical protein [*O. sativa* (japonica)]	Contig7338_at	-1.14	3.15	AAL58119.1	e-43
Putative uncharacterized protein [*O. sativa* (japonica)]	Contig7499_at	1.15	2.66	BAB16470.1	e-62
Unknown protein [*O.sativa* (japonica)]	Contig8064_at	1.01	2.4	BAB44030.1	2e-58
Similar to unknown protein [*A. thaliana*]	Contig8508_at	1.07	3.09	NP_200633.1	3e-51
Unknown protein [*A. thaliana*]	Contig9412_at	1.33	2.01	AAM66944.1	2e-81
Unknown protein [*A. thaliana*]	Contig9412_s_at	1.28	2.15	AAM66944.1	2e-81
Expressed protein [*A. thaliana*]	Contig9438_s_at	-1.02	2.00	NP_567420.1	5e-50
Unknown protein [*A. thaliana*]	Contig9596_at	1.16	2.12	NP_188468.1	e-10
Hypothetical protein F22K18.50 [*A. thaliana*]	Contig9660_at	-1.25	2.52	NP_194206.1	2e-48
Hypothetical protein [*O. sativa* (japonica)]	Contig9763_s_at	-1.01	3.17	BAC19990.1	2e-42
Expressed protein [*A. thaliana*]	Contig9936_at	-1.87	4.36	NP_567820.1	2e-20
Hypothetical protein [*O. sativa* (japonica)]	Contig9951_s_at	-1.36	2.68	BAB92407.1	9e-76
Hypothetical protein [*O. sativa* (japonica)]	Contig9952_at	-1.45	2.38	BAB92407.1	7e-74
P0518C01.34 [*O. sativa* (japonica)]	Contig9958_at	-1.03	2.20	BAB63695.1	3e-75
P0518C01.34 [*O. sativa* (japonica)]	Contig9958_s_at	-1.12	2.15	BAB63695.1	3e-75
Hypothetical protein T2J13.20 [*A. thaliana*]	Contig10346_at	1.18	2.30	NP_190483.1	2e-43
Unknown [*Davidia involucrata*]	Contig10655_at	-1.36	2.16	AAL47004.1	3e-25
Hypothetical protein M3E9.200 [*S.*	Contig10822_at	1.20	4.91	AAL73975.1	2e-68

Annotation	Probe ID	Fold change (Cd treatment *vs* control)		Accession No	E-value
		W6nk2	Zhenong8		
bicolor]					
Hypothetical protein [*O. sativa*]	Contig11363_at	-1.22	2.11	AAK16173.1	3e-87
Putative uncharacterized protein AT4g13500 [*A. thaliana*]	Contig11763_s_at	-1.01	2.01	NP_193086.1	6e-19
Hypothetical protein [*O. sativa* (japonica)]	Contig11963_s_at	-1.15	2.01	BAB64743.1	3e-37
AT5g23040/MYJ24_3 [*A. thaliana*]	Contig12028_at	-1.12	2.08	NP_197695.1	2e-55
Expressed protein [*A. thaliana*]	Contig12132_at	-1.14	2.00	NP_566113.1	3e-69
Expressed protein [*A. thaliana*]	Contig12132_s_at	-1.07	2.43	NP_566113.1	3e-69
Unknown protein [*O. sativa*]	Contig13271_at	-1.71	3.93	AAL58188.1	e-103
Expressed protein [*A. thaliana*]	Contig13277_at	1.68	2.22	NP_564144.1	4e-41
Expressed protein [*A. thaliana*]	Contig13409_at	-1.32	2.06	NP_563991.1	7e-35
Expressed protein [*A. thaliana*]	Contig13457_s_at	-1.78	2.11	NP_567210.1	8e-38
Unknown protein [*A. thaliana*]	Contig13672_at	1.18	2.09	NP_177177.1	2e-26
Similar to cytoskeletal protein [*O. sativa*]	Contig14220_at	1.73	2.01	BAC10806.1	3e-33
Hypothetical protein [*A. thaliana*]	Contig15715_at	1.73	2.31	NP_180777.1	3e-30
Hypothetical protein [*A. thaliana*]	Contig16125_at	1.08	2.47	NP_565301.1	5e-43
Unknown [*A. thaliana*]	Contig18643_at	1.23	3.34	AAM66952.1	3e-15
Hypothetical protein [*A. thaliana*]	Contig18925_at	-1.05	2.12	NP_195074.1	2e-26
Expressed protein [*A. thaliana*]	Contig19088_at	1.37	2.48	NP_568663.1	2e-23
Putative uncharacterized protein [*A. thaliana*]	Contig20033_at	1.06	2.83	NP_198202.1	2e-19
Hypothetical protein [*O. sativa*]	Contig24253_at	-1.40	2.88	AAM08870.1	6e-21
Unknown protein [*A. thaliana*]	Contig25351_at	-1.49	2.94	NP_180876.1	8e-33
Hypothetical protein [*O. sativa*]	Contig4815_at	-1.48	4.55	AAK98749.1	3e-20
Hypothetical protein T7H20.230 [*A. thaliana*]	Contig18853_at	-1.29	2.06	NP_195838.1	3e-17
Hypothetical protein T18B16.70 [*A. thaliana*]	Contig24302_at	1.24	2.91	NP_193645.1	e-23
Unknown protein [*A. thaliana*]	HVSMEb0001J13r2_at	1.99	2.14	AAM13860.1	4e-11
Unnamed protein product [*M. musculus*]	EBma08_SQ002_M18_at	-2.24	-1.90	BAC27870.1	0.5
Hypothetical protein [*O. sativa* (japonica)]	Contig10448_at	-2.52	-1.81	AAM88621.1	6e-34
Putative uncharacterized protein AT4g28770 [*A. thaliana*]	Contig8424_s_at	-3.22	-1.66	NP_194606.1	4e-28
Expressed protein [*A. thaliana*]	Contig8658_at	-3.48	-1.30	NP_565383.1	4e-19
Hypothetical protein [*O. sativa* (japonica)]	HW05J24u_s_at	-2.13	-1.00	BAB55683.1	5e-19
Unknown protein [*O. sativa* (japonica)]	Contig12710_at	-2.00	1.31	AAK20043.1	e-65
Hypothetical protein [*H. vulgare*]	rbags25i16_at	-2.23	1.36	S49173	6e-06
Unknown protein [*O. sativa*]	Contig15356_at	-2.00	1.5	BAB90214.1	6e-52
OSJNBb009e11.14 [*O. sativa*	HS17I17u_s_at	-2.13	-1.31	CAD41545.1	0.031

Annotation	Probe ID	Fold change (Cd treatment vs control) W6nk2	Zhenong8	Accession No	E-value
(japonica)]					
**None**					
none	Contig12421_at	-2.30	3.32	none	none
none	Contig10140_s_at	-2.97	2.13	none	none
none	Contig2279_at	-3.78	2.02	none	none
none	Contig19291_at	-2.01	2.01	none	none
none	Contig2082_x_at	-1.49	2.41	none	none
none	Contig2476_at	-1.09	2.48	none	none
none	Contig2894_s_at	1.03	2.37	none	none
none	rbaal33b21_s_at	1.20	2.31	none	none
none	Contig4670_at	-1.08	2.34	none	none
none	Contig4670_s_at	1.16	2.31	none	none
none	Contig5365_s_at	1.91	2.82	none	none
none	Contig9064_at	1.19	2.11	none	none
none	Contig11066_at	1.65	2.06	none	none
none	Contig12100_at	-1.88	2.66	none	none
none	Contig14088_s_at	-1.34	2.70	none	none
none	Contig14088_x_at	-1.06	2.06	none	none
none	Contig18518_at	-1.51	4.26	none	none
none	Contig18959_at	-1.30	2.44	none	none
none	Contig22849_at	1.28	2.46	none	none
none	Contig25667_s_at	1.31	2.26	none	none
none	basd23g06_s_at	1.06	6.02	none	none
none	EBed02_SQ003_F07_s_at	1.45	2.16	none	none
none	EBem10_SQ004_J15_at	1.06	2.18	none	none
none	EBes01_SQ002_E05_at	1.28	2.43	none	none
none	EBro07_SQ002_G23_s_at	1.37	3.49	none	none
none	HO10P07S_at	-1.27	2.09	none	none
none	HX13K24r_at	-1.09	2.13	none	none
none	HVSMEl0010I15r2_s_at	-1.13	2.19	none	none
none	HVSMEa0005E13r2_s_at	1.55	2.51	none	none
none	HY08M06u_x_at	-1.66	2.39	none	none
none	HS18B10u_s_at	1.99	2.07	none	none
none	HW01P03u_x_at	1.05	2.14	none	none
none	HB32A22r_at	-1.23	2.11	none	none
none	HVSMEa0011H12r2_x_at	1.30	2.17	none	none
none	Contig20565_at	-2.52	-1.47	none	none
none	HVSMEb0014F22f_s_at	-2.12	-1.41	none	none
none	HVSMEf0023D17f_s_at	-2.87	-1.36	none	none
none	HD12C08r_at	-2.85	-1.21	none	none
none	HB18H23r_s_at	-2.09	-1.18	none	none
none	HVSMEb0011H13r2_at	-2.16	-1.14	none	none
none	Contig21965_at	-2.12	-1.11	none	none
none	Contig8425_at	-2.28	-1.11	none	none
none	Contig5807_s_at	-2.77	-1.08	none	none

Annotation	Probe ID	Fold change (Cd treatment *vs* control)		Accession No	E-value
		W6nk2	Zhenong8		
none	HVSMEm0004L13r2_s_at	-2.20	-1.03	none	none
none	Contig26451_at	-2.21	1.10	none	none
none	Contig18687_at	-2.41	1.54	none	none
none	Contig2279_s_at	-3.03	1.76	none	none
