# Supplementary material for: DNA microarray revealed and RNAi plants confirmed key genes conferring low Cd accumulation in barley grains
Source: BMC Plant Biol. 2015 Oct 26;15:259. doi: 10.1186/s12870-015-0648-5 (PMC4623906; doi:10.1186/s12870-015-0648-5)
Supplement: Additional file 8: Table S3. — List of genes up-regulated in both W6nk2 and Zhenong8 after 15 days exposure to 5 μM Cd. (DOC 109 kb) [file 12870_2015_648_MOESM8_ESM.doc]

**Additional file 8**

**Table S3 List of genes up-regulated in both W6nk2 and Zhenong8 after 15 days exposure to 5 μM Cd.**

| Annotation | Probe ID | Fold change  (Cd treatment *vs* control) | | Accession No | E-value |
| --- | --- | --- | --- | --- | --- |
|  |  | W6nk2 | Zhenong8 |  |  |
| **Stress and defense response** |  |  |  |  |  |
| Putative flavanone 3-hydroxylase [*O. sativa* (japonica)] | Contig11212_at | 22.39 | 2.10 | AAL58118.1 | e-72 |
| Putative lipoxygenase [*A. thaliana*] | Contig23795_at | 2.49 | 2.48 | NP_177396.1 | 8e-12 |
| Methyljasmonate-inducible lipoxygenase 2 [*H. vulgare*] | Contig2306_s_at | 2.52 | 3.17 | T06190 | 5e-95 |
| Senescence-associated protein [*A. thaliana*] | Contig9398_s_at | 3.29 | 3.37 | NP_569030.1 | 9e-17 |
| Thionin precursor [*H. vulgare*] | Contig1580_x_at | 2.63 | 5.16 | S22515 | 6e-71 |
| Thionin [*H. vulgare*] | Contig1570_s_at | 2.81 | 16.06 | AAB21531.1 | e-76 |
| ABA-induced protein [*H. vulgare subsp*. vulgare] | Contig6276_s_at | 3.13 | 11.1 | T04417 | 5e-80 |
| **Transport** |  |  |  |  |  |
| Copper chaperone homolog CCH [*O. sativa*] | Contig6788_at | 3.02 | 4.54 | T50779 | 6e-29 |
| **Carbohydrate metabolism** |  |  |  |  |  |
| Putative Glucan 1,3-beta-glucosidase precursor [*O. sativa* (japonica)] | Contig9267_s_at | 6.97 | 2.26 | AAM08620.1 | 2e-53 |
| Enolase 1 (2-phosphoglycerate dehydratase 1) [*Z. mays*] | Contig1298_at | 2.64 | 2.50 | P26301 | e-116 |
| Putative Glucan 1,3-beta-glucosidase precursor [*O. sativa* (japonica)] | HU10K22u_s_at | 3.15 | 5.19 | AAM08620.1 | 5e-44 |
| Apyrase GS52 [*Glycine soja*] | Contig3332_at | 2.26 | 5.40 | AAG32960.1 | 4e-55 |
| Serine carboxypeptidase II, CP-MII [*H. vulgare*] | Contig7697_at | 3.09 | 2.35 | T05701 | e-114 |
| Putative acid phosphatase [*H. vulgare subsp*. vulgare] | Contig2433_s_at | 3.45 | 2.93 | CAB71336.1 | e-112 |
| **Signal transduction** |  |  |  |  |  |
| Protein kinase homolog [*O. sativa*] | Contig13973_at | 2.37 | 2.51 | T03444 | 3e-97 |
| **Fat metabolism** |  |  |  |  |  |
| Similar to lipases [*A. thaliana*] | Contig6611_at | 3.78 | 8.49 | AAF63138.1 | e-71 |
| **Unknown classified** |  |  |  |  |  |
| Hypothetical protein [*O. sativa* (japonica)] | Contig11926_s_at | 2.34 | 2.24 | BAB85334.1 | e-20 |
| Unnamed protein product [*O. sativa* (japonica)] | Contig6075_at | 4.82 | 33.22 | BAA94780.1 | 2e-76 |
| Unknown protein [*O. sativa* (japonica)] | Contig6170_s_at | 2.11 | 2.40 | BAB78620.1 | 4e-30 |
| OSJNBa0086B14.7 [*O. sativa* (japonica)] | Contig11993_at | 3.83 | 4.09 | CAD40835.1 | 4e-12 |
| **None** |  |  |  |  |  |
| none | Contig6699_s_at | 8.84 | 2.16 | none | none |
| none | Contig24662_at | 2.85 | 2.18 | none | none |
| none | HA10M12u_s_at | 2.06 | 2.27 | none | none |
| none | Contig18112_at | 2.27 | 2.30 | none | none |
| none | HV_CEa0009K20r2_x_at | 9.94 | 2.77 | none | none |
| none | HV_CEa0009K20r2_at | 6.92 | 3.05 | none | none |
| none | Contig6701_s_at | 12.49 | 3.21 | none | none |
| none | EBro01_SQ005_J04_at | 8.95 | 3.34 | none | none |
| none | Contig16541_at | 3.30 | 4.39 | none | none |
| none | HVSMEb0002K02r2_s_at | 4.14 | 4.80 | none | none |
| none | Contig8256_at | 2.28 | 2 | none | none |
