# Supplementary material for: DNA microarray revealed and RNAi plants confirmed key genes conferring low Cd accumulation in barley grains
Source: BMC Plant Biol. 2015 Oct 26;15:259. doi: 10.1186/s12870-015-0648-5 (PMC4623906; doi:10.1186/s12870-015-0648-5)
Supplement: Additional file 9: Table S4. — List of genes down-regulated in both W6nk2 and Zhenong8 after 15 days exposure to 5 μM Cd. (DOC 130 kb) [file 12870_2015_648_MOESM9_ESM.doc]

**Additional file 9**

**Table S4** List of genes down-regulated in both W6nk2 and Zhenong8 after 15 days exposure to 5 μM Cd.

| Annotation | Probe ID | Fold change  (Cd treatment *vs* control) | | Accession No | E-value |
| --- | --- | --- | --- | --- | --- |
|  |  | W6nk2 | Zhenong8 |  |  |
| **Stress and defense response** |  |  |  |  |  |
| Cu/Zn superoxide dismutase [*T. aestivum*] | Contig3197_at | -10.37 | -4.51 | T06800 | 9e-83 |
| Gag polyprotein [*Human immunodeficiency virus* 1] | MitoContig15_at | -8.68 | -16.0 | BAB12557.1 | 0.1 |
| **Transport** |  |  |  |  |  |
| Putative membrane protein At2g01770.1 [*A. thaliana*] | Contig25699_at | -3.23 | -2.36 | NP_178286.1 | 3e-06 |
| RAB5A protein [*O. sativa*] | Contig6921_at | -2.27 | -3.33 | CAC19792.1 | 3e-83 |
| **Transcription** |  |  |  |  |  |
| Histone H2A.9 [*T. aestivum*] | Contig286_s_at | -4.17 | -2.45 | S53519 | 7e-51 |
| Similar to nonhistone chromosomal protein [*M. musculus*] | HVSMEc0003G09f_x_at | -4.99 | -8.95 | XP_140242.1 | 0.4 |
| **Photosynthesis** |  |  |  |  |  |
| Photosystem I P700 apoprotein A1 [*T. aestivum*] | HV_CEa0013J19f_at | -7.69 | -8.80 | NP_114259.1 | 8e-75 |
| Photosystem I subunit IX [*N. tabacum*] | HVSMEa0022N20f_at | -2.92 | -5.02 | NP_054521.1 | 2e-07 |
| Photosystem I P700 chlorophyll A apoprotein A1 [*Z. mays*] | HVSMEc0016D02f_at | -15.63 | -10.71 | P04966 | 7e-52 |
| photosystem I P700 apoprotein A1 [*T. aestivum*] | HVSMEc0016J13f_at | -11.51 | -11.06 | NP_114259.1 | 2e-17 |
| **Signal transduction** |  |  |  |  |  |
| SLAM family member 7, 19A protein [*Homo sapiens*] | EBro02_SQ006_D14_s_at | -3.51 | -5.58 | CAB81950.2 | 0.6 |
| **Protein synthesis** |  |  |  |  |  |
| Ribosomal protein S14 [*T. aestivum*] | HVSMEc0009D09f_at | -8.64 | -8.20 | NP_114257.1 | 2e-38 |
| **Unknown classified** |  |  |  |  |  |
| Hypothetical protein [*O. elata subsp. hookeri*] | ChlorContig11_s_at | -4.08 | -6.11 | NP_084748.1 | 3e-13 |
| Hypothetical protein [*O. elata subsp. hookeri*] | ChlorContig11_x_at | -2.63 | -3.38 | NP_084748.1 | 3e-13 |
| Hypothetical protein [*O. sativa* (japonica)] | ChlorContig9_at | -2.44 | -3.09 | AAM08574.1 | 2e-12 |
| Putative uncharacterized protein At4g27350 [*A. thaliana*] | Contig15977_at | -2.19 | -2.13 | NP_194465.1 | 4e-28 |
| Expressed protein [*A. thaliana*] | Contig17314_at | -2.10 | -3.77 | NP_567276.1 | 7e-91 |
| Hypothetical protein [*O. sativa* (japonica)] | Contig3364_at | -2.59 | -3.66 | AAM08574.1 | 6e-32 |
| Phosphate-induced protein 1-like protein [*Pennisetum ciliare*] | Contig9813_at | -4.78 | -3.13 | AAK15505.1 | 5e-59 |
| Hypothetical protein F6E13.8 [*A. thaliana*] | HT09L15u_at | -10.63 | -3.44 | T00675 | 3e-28 |
| Uncharacterized protein ycf68 [*O. sativa* (japonica)] | HVSMEa0020P01f_at | -17.7 | -13.94 | NP_039436.1 | 8e-19 |
| Hypothetical protein [*N. tabacum*] | HVSMEc0003A01f_at | -3.64 | -3.92 | NP_054552.1 | 2e-06 |
| Putative uncharacterized protein srbc-65 [*Caenorhabditis elegans*] | HVSMEc0004C11f_x_at | -2.85 | -3.75 | NP_507189.1 | 0.3 |
| Hypothetical protein [*O. elata subsp. hookeri*] | HVSMEc0005O17f_x_at | -2.02 | -2.70 | NP_084748.1 | 2e-13 |
| Hypothetical protein [*Plasmodium yoelii yoelii*] | HVSMEc0009K16f_s_at | -2.43 | -3.26 | EAA16547.1 | 0.1 |
| Hypothetical protein [*O. elata subsp. hookeri*] | HVSMEc0011H17f_x_at | -2.57 | -3.19 | NP_084748.1 | e-13 |
| Hypothetical protein [*O. elata subsp. hookeri*] | HVSMEc0014G01f_x_at | -2.45 | -6.06 | NP_084748.1 | 2e-12 |
| Hypothetical protein ORF35 [*Picea abies*] | HVSMEc0019G06f_at | -4.52 | -3.44 | T11812 | 0.012 |
| Putative uncharacterized protein F8M21_130 [*A. thaliana*] | HVSMEg0017A08r2_at | -2.54 | -3.52 | NP_197028.1 | 9e-06 |
| Unnamed protein product [*M. musculus*] | MitoContig11_at | -2.51 | -3.07 | BAC26016.1 | 0.6 |
| ORF-98; hypothetical sterility protein 1 [*P. vulgaris*] | HVSMEc0019O14f_at | -7.20 | -6.72 | S26981 | 3e-15 |
| Uncharacterized protein | Contig10642_at | -3.94 | -5.75 | BAC10843.1 | 8e-40 |
| Unnamed protein product [*H. sapiens*] | MitoContig10_x_at | -6.16 | -20.7 | BAB71593.1 | 0.6 |
| **None** |  |  |  |  |  |
| none | Contig15194_at | -2.62 | -3.20 | none | none |
| none | Contig21096_at | -2.47 | -3.05 | none | none |
| none | Contig23342_at | -3.67 | -2.62 | none | none |
| none | Contig24866_at | -4.63 | -4.59 | none | none |
| none | Contig7914_at | -6.96 | -8.26 | none | none |
| none | HU10I18u_at | -2.63 | -4.06 | none | none |
| none | HV_CEa0012H15r2_at | -2.06 | -3.23 | none | none |
| none | HVSMEc0001D14f_x_at | -3.34 | -5.34 | none | none |
| none | HVSMEc0001F13f_at | -3.85 | -2.77 | none | none |
| none | HY02K24u_at | -3.37 | -2.46 | none | none |
