# Supplementary material for: DNA microarray revealed and RNAi plants confirmed key genes conferring low Cd accumulation in barley grains
Source: BMC Plant Biol. 2015 Oct 26;15:259. doi: 10.1186/s12870-015-0648-5 (PMC4623906; doi:10.1186/s12870-015-0648-5)
Supplement: Additional file 10: Figure S6. — Cd-induced differential genes expression except for transport related in leaves of two barley genotypes. (DOC 407 kb) [file 12870_2015_648_MOESM10_ESM.doc]

**Additional file 10**

**ID**

**W6nk2**

**Zhenong8**

**Annotation**


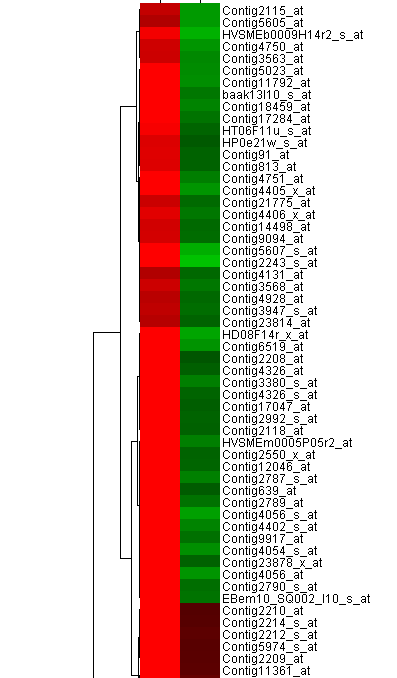

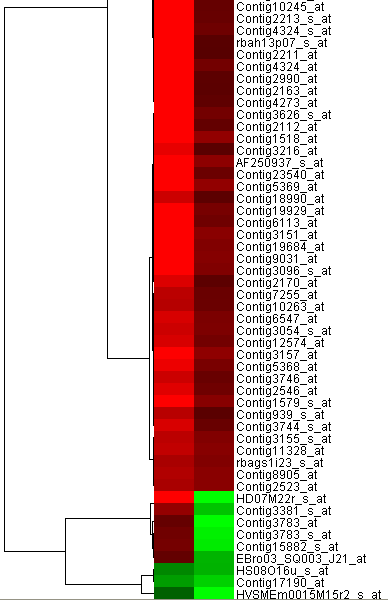

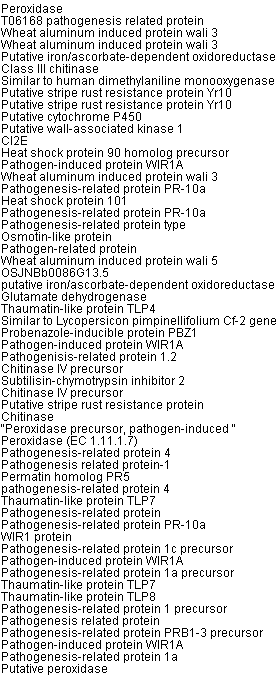

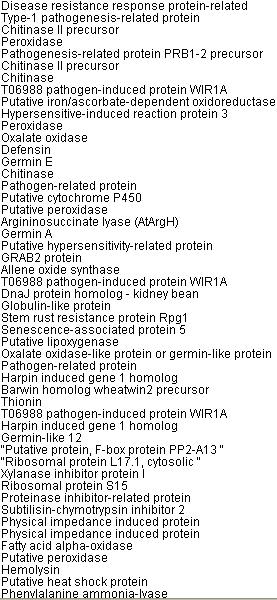

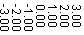

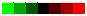

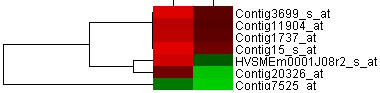

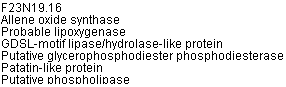

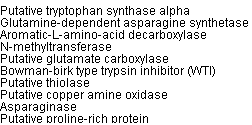

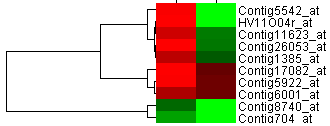

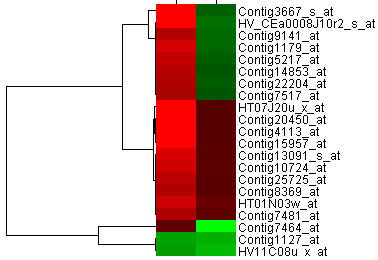

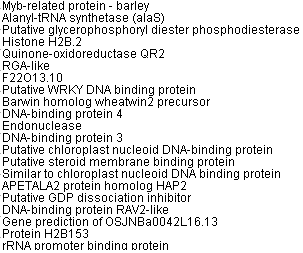

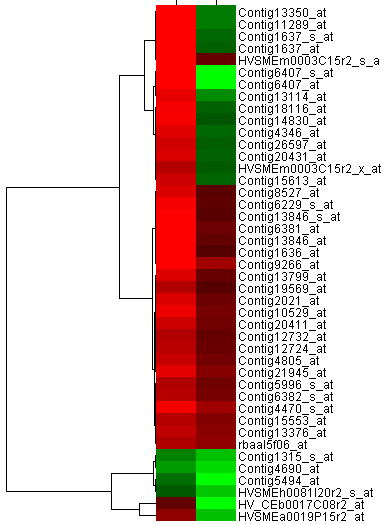

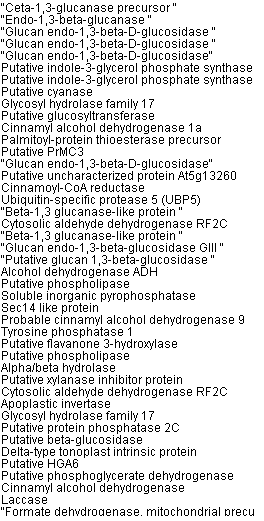

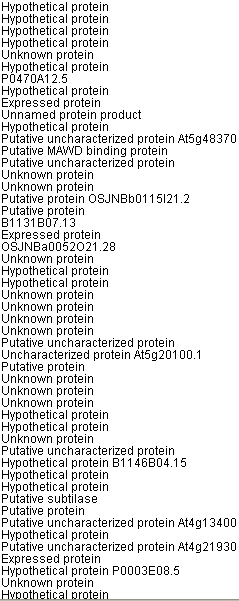

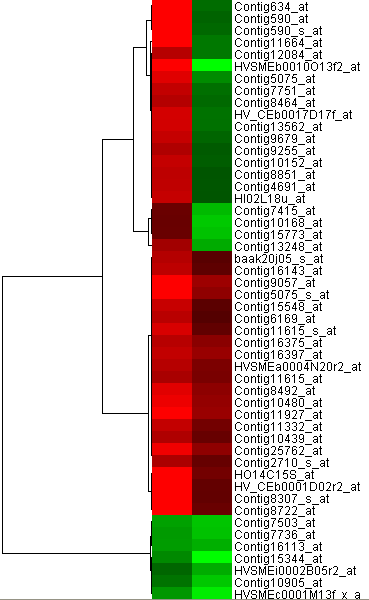

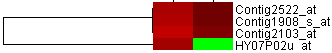

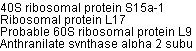

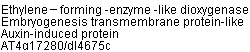

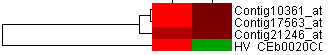

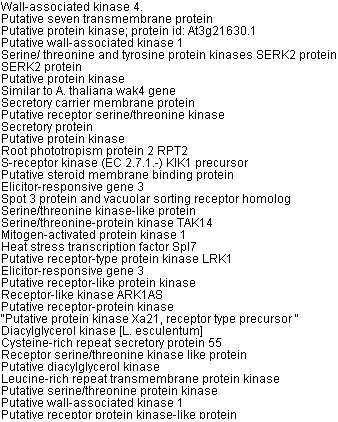

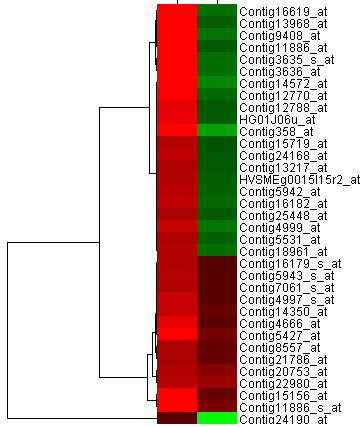

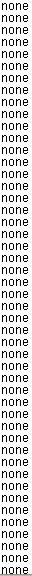

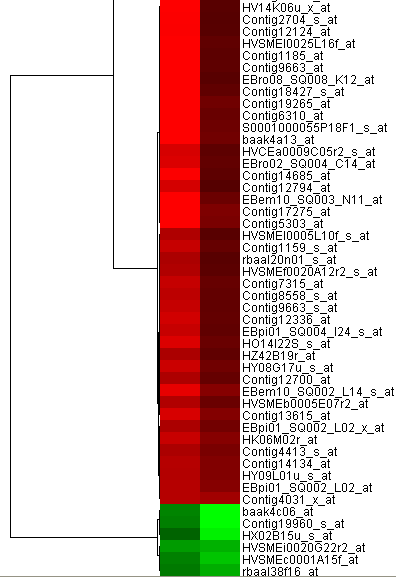

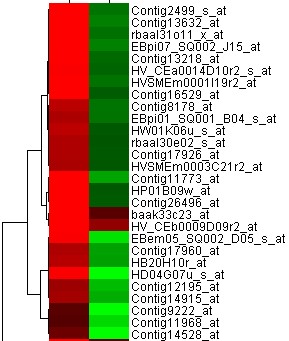

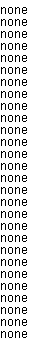


**Fig. S6** Cd-induced differential genes expression except for transport related in leaves of two barley genotypes. Heat map visualises the expression of genes up-regulated in W6nk2 and down-regulated/no-change in Zhenong8, and no change in W6nk2 and down-regulated in Zhenong8 (Cd vs control) after Cd exposure for 15 d. The contig IDs and annotations are listed on the right. Red, green and black indicate genes that increased, decreased and showed equal levels of expression, respectively, as compared to the control. The contig ID and annotation of each gene are listed on the right of the figure. The identity and accession numbers of genes are listed in Table S1.
