# Supplementary material for: DNA microarray revealed and RNAi plants confirmed key genes conferring low Cd accumulation in barley grains
Source: BMC Plant Biol. 2015 Oct 26;15:259. doi: 10.1186/s12870-015-0648-5 (PMC4623906; doi:10.1186/s12870-015-0648-5)
Supplement: Additional file 11: Figure S7. — Integrated schematic of the mechanisms involved in Cd high accumulation and tolerance. (PDF 183 kb) [file 12870_2015_648_MOESM11_ESM.pdf]

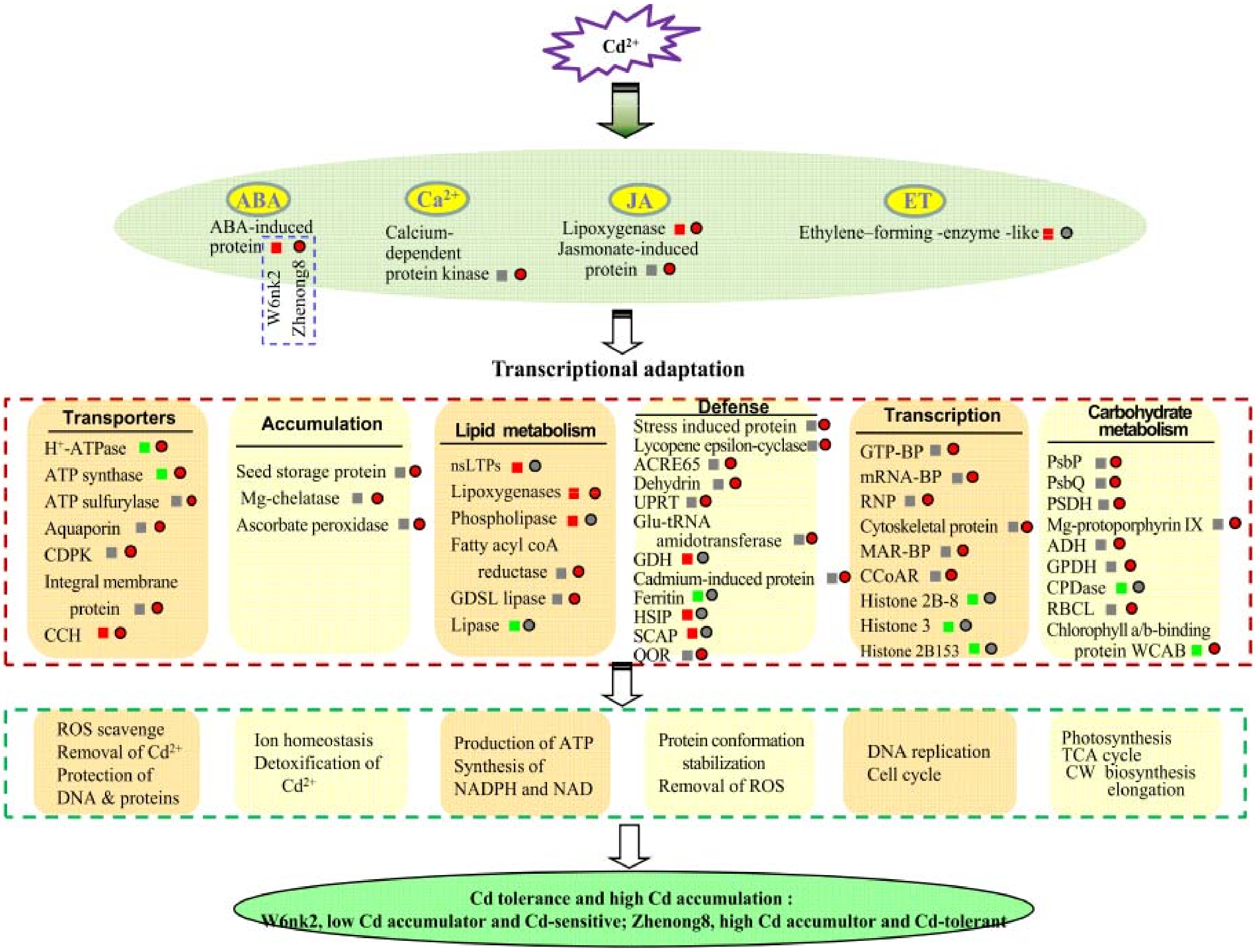

Fig. S7 Integrated schematic of the mechanisms involved in Cd high accumulation and tolerance. Red, grey, and green symbols represent genes that are up-regulated, unchanged and down-regulated by Cd (Cd vs control), respectively. Coloured squares and circles indicate barley genotypes W6nk2 and Zhenong8, respectively. ABA, Abscisic acid; ADH, Alcohol dehydrogenase; CDPK, Calcium-dependent protein kinase; CCH, Copper chaperone homolog; CCoAR, Cinnamoyl-CoA reductase; CPDase, Cyclic phosphodiesterase; GDH, Glutamate dehydrogenase; GAPDH, Glyceraldehyde-3- phosphate dehydrogenase B; GTP-BP, GTP-binding protein; HSIP, Hypersensitive-induced reaction protein; JA, jasmonate; MAR-BP, MAR-binding protein; mRNA–BP, mRNA-binding protein; nsLTPs, Nonspecific lipid-transfer protein; PSDH, phosphate dehydrogenase; QOR, Quinone oxidoreductase; RBCL, Ribulose-1,5-bisphosphate carboxylase/oxygenase; RNP, Ribonucleoprotein; SCAP, Senescence-associated protein; UPRT, Uracil phosphoribosyltransferase.
