# Supplementary material for: DNA microarray revealed and RNAi plants confirmed key genes conferring low Cd accumulation in barley grains
Source: BMC Plant Biol. 2015 Oct 26;15:259. doi: 10.1186/s12870-015-0648-5 (PMC4623906; doi:10.1186/s12870-015-0648-5)
Supplement: Additional file 12: Table S5. — The sequence of primers for RT-PCR. (DOC 73 kb) [file 12870_2015_648_MOESM12_ESM.doc]

**Additional file 11**

**Table S5** The sequence of primers for RT-PCR

| Gene nnotation | Accession No. | Forward primer（5’- 3’） | Reverse primer（5’- 3’） |
| --- | --- | --- | --- |
| ZIP-like zinc transporter | AAK69429.1 | TCAGGCCATTGGTGGCAGCG | CCAGCCCCAGCAGCGATACC |
| Zinc transporter protein ZIP1 | BAC21508.1 | AGTTCAGGCAAAGTTCAAGG | CCGTCGGGCTGTTCTCGTT |
| ABC transporter | BAB93292.1 | AGTTAGCCAGGAGCCTACACTC | TAGCCGCGTTCTCAACGTC |
| ABC transporter family protein | NP_200978.1 | CGGTGAGCAGGGTGTACCGC | CAGCAAGGCCCCACGCAAGA |
| MRP-like ABC transporter | BAB62557.1 | AGTCTGGCCGTCGATAAT | TCCGCCGTAAAGTAAGTG |
| P-type ATPase | CAC40030.1 | TGCGAACACCGTGCCGTTGA | GCTCGTACCCATGAGGCTTCGC |
| Iron-phytosiderophore transporter | AAG17016.2 | AAGCACACGGTTCCAGCTC | ACGATCAAGGTCGTAGTGGTC |
| Proteinase inhibitor-related protein | S53102 | CAGGCCGTCCTGGTCATGGG | ACTTCTTGCATGGCCCGCCG |
| Glutathione transferase F3 | CAD29476.1 | CTCATGCCGCCGGACCTTGG | ACGGAGAGTCCGCGAGTCGT |
| Pathogen-induced protein WIR1A | Q01482 | CATACCCCGGCCGGAAGTGC | CGTGCATGTCTAGGCCGCCA |
| Pathogen-related protein | P16273 | AAGCCACTGACGCGGGAGGA | GGGGAACGCCGTGAGGAACG |
| Pathogenesis-related protein PR-10a | AAF85972.1 | CAGTGACCGACGGACAAGAAC | GAGAAGACCACCTTCCACAGC |
| T06168 pathogenesis related protein | P16273 | TCTCTGGGCAGCCACCAGCA | TGTGTGGCGACGTCGTTGGG |
| Heat shock protein 101 | AAF01280.1 | TTCGCCGTCACTCCCCCAGT | TCAATGCGCCGGTTGTCCCC |
| Phosphoribosylanthranilate transferase | AAM19104.1 | CGCTCCGGAGTGGCCATCAA | TTCGCGCCATTCCTCGCGTC |
| Glutamine-dependent asparagine synthetase | AAK49456.1 | GATGATTTCTAATCCTTTTC | GCCTCTTTGAACTATTGT |
| Putative indole-3-glycerol phosphate synthase | AAM64536.1 | TAGTAGTTGCGAGACCACCG | ATATCGTTTATTATCCCG |
| Wheat aluminum induced protein wali 5 | JQ2361 | CCAAGCTGGCGGCGATCCTC | TCTTCATGTCGGCGCAGCGG |
| Wheat aluminum induced protein wali 3 | JQ2360 | CGCCAACGCCGAGTTTCCCA | TCGTCTCCACAACGCGGCAG |
| Actin (Control) | AY145451 | TGGCTGACGGTGAGGACA | CGAGGGCGACCAACTATG |
