# Supplementary material for: DNA microarray revealed and RNAi plants confirmed key genes conferring low Cd accumulation in barley grains
Source: BMC Plant Biol. 2015 Oct 26;15:259. doi: 10.1186/s12870-015-0648-5 (PMC4623906; doi:10.1186/s12870-015-0648-5)
Supplement: Additional file 13: Figure S8. — Molecular cloning and RNAi analysis of HvZIP3 and HvZIP8. (DOC 153 kb) [file 12870_2015_648_MOESM13_ESM.doc]

**Additional file 13**

D

300bp

1kb *ZIP3 ZIP8 ZIP3 ZIP8*

C

2700bp

M Vector

W6nk2 Zhenong8M*ZIP3 ZIP8 ZIP3 ZIP8*

M W6nk2 Zhenong8


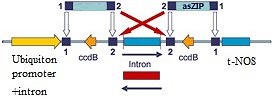


E


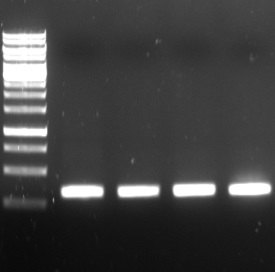


A B


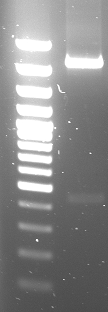


M Vector

450bp

2700bp


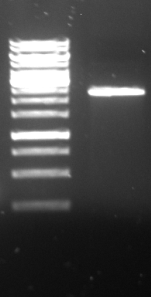

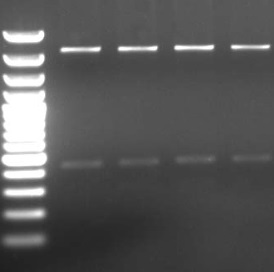


2700bp

450bp

**Fig. S8** Molecular cloning and RNAi analysis of *HvZIP3* and *HvZIP8*. pENTR4 vector was digested with XmnI and EcoRV (A) and the PCR product of gel extraction (B). PCR fragments of *HvZIP* genes (C) and pENTR4 clones digested with PstI and NcoI (D) in both Zhenong8 and W6nk2. (E) Schematic representation of the antisense expression cassette. The expression cassette was assembled in the ubiquition introns of pStargate, which contains the maize ubiquitin promoter (UbiP) for expressing the fragment in barley plants. The *ccdB* gene encodes a cytotoxic protein, which prevents growth of in the sensitive host if fragments have not recombined away.
