# Supplementary material for: DNA microarray revealed and RNAi plants confirmed key genes conferring low Cd accumulation in barley grains
Source: BMC Plant Biol. 2015 Oct 26;15:259. doi: 10.1186/s12870-015-0648-5 (PMC4623906; doi:10.1186/s12870-015-0648-5)
Supplement: Additional file 14: Figure S9. — Sequence aligments of ZIP3 and ZIP8. (DOC 64 kb) [file 12870_2015_648_MOESM14_ESM.doc]

**Additional file 14**

**A**

Score = 553 bits (612), Expect = 3e-154

Identities = 310/311 (99%), Gaps = 1/311 (0%)

Strand=Plus/Minus

Query 1.. ACTAGGGACATGTAGATTAGAATCCCTGCCGAGGCTGAGTTGAAGACTCCCTCAATAATG 60

||||||||||||||||||||||||||||||||||||||||||||||||||||||||||||

Sbjct 965 ACTAGGGACATGTAGATTAGAATCCCTGCCGAGGCTGAGTTGAAGACTCCCTCAATAATG 906

Query 61. AAGGCAGTAGAGCTATGCACATTATAGCTAGATGAAACCGCAATCCCTAGCACGATGCCC 120

||||||||||||||||||||||||||||||||||||||||||||||||||||||||||||

Sbjct 905 AAGGCAGTAGAGCTATGCACATTATAGCTAGATGAAACCGCAATCCCTAGCACGATGCCC 846

Query 121 ACTGGTGCGGTAAGGGAGAAAAACGTTGCCATGATGATGGTTGCCCTTACCTTGAAATTA 180

||||||||||||||||||||||||||||||||||||||||||||||||||||||||||||

Sbjct 845 ACTGGTGCGGTAAGGGAGAAAAACGTTGCCATGATGATGGTTGCCCTTACCTTGAAATTA 786

Query 181 GCCTGAACAATGCAACCACCCAAGCCTATGCCTTCAAAGAATTGATGGAAGCTGAGGGCA 240

||||||||||||||||||||||||||||||||||||||||||||||||||||||||||||

Sbjct 785 GCCTGAACAATGCAACCACCCAAGCCTATGCCTTCAAAGAATTGATGGAAGCTGAGGGCA 726

Query 241 CCGACCAGAGGCTTGATGGTGGATGGCCTCACAGATGCTCCTAATGACACGCCAATTATC 300

||||||||||||||||||||||||||||||||||||||||||||||||||||||||||||

Sbjct 725 CCGACCAGAGGCTTGATGGTGGATGGCCTCACAGATGCTCCTAATGACACGCCAATTATC 666

Query 301 ACT-AATGCAC 310

||| |||||||

Sbjct 665 ACTGAATGCAC 655

**B**

Score = 257 bits (284), Expect = 3e-65

Identities = 219/269 (81%), Gaps = 1/269 (0%)

Strand=Plus/Minus

Query 1.. AGGTGGACCAGCCCCTTGTACTTGAGGATCCCTGCGGCGGCCCCCCTGACGATCCCTTGT 60

|||| ||||||| || |||| |||||||||||||||||||| | |||| |||||||||

Sbjct 962 AGGTCGACCAGCGCCATGTAGTTGAGGATCCCTGCGGCGGCGGCGCTGAGGATCCCTTGG 903

Query 61. TTGATGAGGGTGTTGGGGCTGTTTTCGTTGATAACAGAGGATATCCCGATGCCGATCATT 120

|||||||||||||||||||||| |||||| ||||||||||||||||||||||||||

Sbjct 902 GTGATGAGGGTGTTGGGGCTGTTCTCGTTGTAAACAGAGGATATCCCGATGCCGATCACG 843

Query 121 ACCCCGACCAGTGTGGAGTGTGAGAAGAAGATCACCATCTGCATCCCGGACTTTGACCGG 180

||||||||| | |||| | |||||||||||| | ||||| ||| | ||||||| ||||

Sbjct 842 ACCCCGACCGGCGTGGTGAGTGAGAAGAAGAGCGCCATCAGCAGCACGGACTTCAGCCGG 783

Query 181 AACTTGTCCGGAACGATCCATCCTCCAATGCCTTTTTCTT-GGCGAACTCACGGAACGTC 239

|||||| || ||||||| |||||||| | ||| | ||| | ||||| | |||| ||

Sbjct 782 AACTTGGCCTGAACGATGCATCCTCCGAGCCCTATCCCTTCGAAGAACTGATGGAAAGTT 723

Query 240 TGCGCGAGCTCTAGAGTTCTGATCGCGCT 268

|||||| | | || | |||||||| |||

Sbjct 722 AGCGCGACCACCAGTGGTCTGATCGTGCT 694

**C**

Score = 522 bits (578), Expect = 5e-145

Identities = 306/317 (97%), Gaps = 0/317 (0%)

Strand=Plus/Plus

Query 1.. GTGCACTCGGTGATCATCGGCATGTCTCTCGGCGCATCCCAAAGCGCCAGCACGATCAGA 60

||||||||||||||||||||||||||||||||||||||||| ||||||||||||||||||

Sbjct 646 GTGCACTCGGTGATCATCGGCATGTCTCTCGGCGCATCCCAGAGCGCCAGCACGATCAGA 705

Query 61. CCACTGGTGGTCGCGCTAACTTTACATCTATTCTTCTAAGGGATAGGGCTCGGAGGATGC 120

||||||||||||||||||||||| |||| |||||| |||||||||||||||||||||||

Sbjct 706 CCACTGGTGGTCGCGCTAACTTTCCATCAGTTCTTCGAAGGGATAGGGCTCGGAGGATGC 765

Query 121 ATCGTTCAGGCCAAGTTCCGGCTGAAGTCCGTGCTGCTGATGGCGCTCTTCTTCTCACTC 180

||||||||||||||||||||||||||||||||||||||||||||||||||||||||||||

Sbjct 766 ATCGTTCAGGCCAAGTTCCGGCTGAAGTCCGTGCTGCTGATGGCGCTCTTCTTCTCACTC 825

Query 181 ACCACGCCGGTCGGGGTCGTGATCGGCATCGGGATATCCTCTGTTTACACCGAGAACAGC 240

||||||||||||||||||||||||||||||||||||||||||||||||| ||||||||||

Sbjct 826 ACCACGCCGGTCGGGGTCGTGATCGGCATCGGGATATCCTCTGTTTACAACGAGAACAGC 885

Query 241 CCCAACACCCTCATCCCCCAATGGATCCTCATCGCCGCCGCCGCAGGGATTCTCAACTAC 300

||||||||||||||| ||||| ||||||||| |||||||||||||||||| |||||||||

Sbjct 886 CCCAACACCCTCATCACCCAAGGGATCCTCAGCGCCGCCGCCGCAGGGATCCTCAACTAC 945

Query 301 ATGGCGCTGGTCAACCT 317

|||||||||||| ||||

Sbjct 946 ATGGCGCTGGTCGACCT 962

**Fig. S9** Sequence aligments of ZIP3 and ZIP8. (A) ZIP3 from W6nk2, (B) ZIP8 from W6nk2, (C) ZIP8 from Zhenong8. Query: cloning region of ZIP allele, Sbjct: coding region of ZIP cDNA RefSeq from Genbank.
